# Supplementary material for: Cost-effectiveness analysis and budget impact of rivaroxaban compared with dalteparin in patients with cancer at risk of recurrent venous thromboembolism
Source: BMJ Open. 2020 Nov 19;10(11):e039057. doi: 10.1136/bmjopen-2020-039057 (PMC7678368; doi:10.1136/bmjopen-2020-039057)
Supplement: Supplementary data [file bmjopen-2020-039057supp001.pdf]

Supplementary data file – Table S1

Manuscript title: Cost-effectiveness analysis and budget impact of rivaroxaban in cancer patients at risk of recurrent venous thromboembolism

Table S1. Transition probabilities used in the cost-effectiveness model

	Rivaroxaban (95% CI)	LMWH (95% CI)	Distribution	Reference
**Recurrent VTE**				
0–6 months	0.040 (0.020 – 0.090)	0.110 (0.070 – 0.160)	Beta	[1]
6–12 months	0.040 (0.031 – 0.050)		Beta	[2]
1–2 years	0.034 (0.027 – 0.042)		Beta	[2]
2–3 years	0.021 (0.014 – 0.029)		Beta	[2]
3–4 years	0.016 (0.009 – 0.026)		Beta	[2]
4–5 months	0.013 (0.006 – 0.024)		Beta	[2]
**Type of recurrent VTE**				
Symptomatic PE	17.4% (α = 4, β = 19)		Dirichlet	[1]
Incidental PE	30.4% (α = 7, β = 16)		Dirichlet	[1]
DVT	43.5% (α = 10, β = 13)		Dirichlet	[1]
Fatal PE	8.7% (α = 2, β = 21)		Dirichlet	[1]
**MB**				
0–6 months	0.060 (0.030 – 0.110)	0.040 (0.020 – 0.080)	Beta	[1]
Beyond 6 months treatment	0.008 (0.006 – 0.010)		Beta	[3]
**Type of MB**				
ICH	10% (α = 5, β = 45)		Dirichlet	[3]
Non-ICH MB	86% (α = 43, β = 7)		Dirichlet	[3]
Fatal MB	4% (α = 2, β = 48)		Dirichlet	[3]
**CRNMB**				
0–6 months	0.130 (0.090 – 0.190)	0.040 (0.020 – 0.090)	Beta	[1]
Beyond 6 months treatment	0.008 (0.006 – 0.010)		Beta	[3]
**PTS**				
0–6 months	0.015 (0.011 – 0.019)		Beta	[4]
6–12 months	0.012 (0.009 – 0.015)		Beta	[4]
12–18 months	0.008 (0.006 – 0.010)		Beta	[4]
18–24 months	0.025 (0.023 – 0.019)		Beta	[4]
24–30 months	0.011 (0.008 – 0.014)		Beta	[4]
30–36 months	0.006 (0.005 – 0.008)		Beta	[4]
3–4 years	0.001 (0.0008 – 0.0013)		Beta	[4]
4–5 years	0.001 (0.0008 – 0.0013)		Beta	[4]
**CTEPH (annual risk)**	0.0057 (0.0002 – 0.012)		Beta	[5]
**Mortality (annual risk)**				
0–1 years	0.230 (0.200 – 0.390)		Beta	[6]
1–2 years	0.104 (0.088 – 0.180)		Beta	[6]
2–3 years	0.058 (0.055 – 0.120)		Beta	[6]
3–4 years	0.046 (0.043 – 0.068)		Beta	[6]
4–5 years	0.032 (0.030 – 0.073)		Beta	[6]
**Relative risk of recurrent VTE, MB, and CRNMB for LMWH versus placebo, used in scenario 5**				
Recurrent VTE (any)	5.170		Fixed	[7]
MB	0.242		Fixed	[7]
CRNMB	1.000		Fixed	[7]

**Drug-specific distribution of the type of VTE, used in scenario 6**				
Symptomatic PE	28.6% (α = 2, β = 5)	12.5% (α = 2, β = 14)	Dirichlet	[1]
Incidental PE	14.3% (α = 1, β = 6)	37.5% (α = 6, β = 10)	Dirichlet	[1]
DVT	42.9% (α = 3, β = 4)	43.8% (α = 7, β = 9)	Dirichlet	[1]
Fatal PE	14.3% (α = 1, β = 6)	6.3% (α = 1, β = 15)	Dirichlet	[1]
**Drug-specific distribution of the type of MB, used in scenario 6**				
ICH	6.1% (α = 2, β = 31)	17.6% (α = 3, β = 14)	Dirichlet	[3]
Non-ICH MB	93.9% (α = 31, β = 2)	70.6% (α = 12, β = 5)	Dirichlet	[3]
Fatal MB	0% (α = 0, β = 33)	11.8% (α = 2, β = 15)	Dirichlet	[3]

Abbreviations: CI, confidence interval; CRNMB, clinically relevant non-major bleeding; CTEPH, chronic thromboembolic pulmonary hypertension; DVT, deep vein thrombosis; ICH, intracranial haemorrhage; LMWH, low-molecular weight heparin; MB, major bleeding; PE, pulmonary embolism; PTS, post-thrombotic syndrome; SE, standard error; VTE, venous thromboembolism

References

1. Young AM, Marshall A, Thirlwall J, Chapman O, Lokare A, Hill C, et al. Comparison of an Oral Factor Xa Inhibitor With Low Molecular Weight Heparin in Patients With Cancer With Venous Thromboembolism: Results of a Randomized Trial (SELECT-D). J Clin Oncol. 2018;36(20):2017–23.
2. Cohen AT, Katholing A, Rietbrock S, Bamber L, Martinez C. Epidemiology of first and recurrent venous thromboembolism in patients with active cancer. Thromb Haemost. 2017;117(01):57–65.
3. Raskob GE, van Es N, Verhamme P, Carrier M, Di Nisio M, Garcia D, et al. Edoxaban for the Treatment of Cancer-Associated Venous Thromboembolism. N Engl J Med. 2018;378(7):615–24.
4. Prandoni P, Villalta S, Bagatella P, Rossi L, Marchiori A, Piccioli A, et al. The clinical course of deep-vein thrombosis. Prospective long-term follow-up of 528 symptomatic patients. Haematologica. 1997;82(4):423–8.
5. Klok FA, van Kralingen KW, van Dijk APJ, Heyning FH, Vliegen HW, Huisman M V. Prospective cardiopulmonary screening program to detect chronic thromboembolic pulmonary hypertension in patients after acute pulmonary embolism. Haematologica. 2010;95(6):970–5.
6. The Netherlands Cancer Registry. Survival statistics - all tumours 1961-2015 [Internet].
7. Napolitano M, Saccullo G, Malato A, Sprini D, Ageno W, Imberti D, et al. Optimal duration of low molecular weight heparin for the treatment of cancer-related deep vein thrombosis: the Cancer-DACUS Study. J Clin Oncol. 2014;32(32):3607–12.

Supplementary data file – Table S2

Manuscript title: Cost-effectiveness analysis and budget impact of rivaroxaban in cancer patients at risk of recurrent venous thromboembolism

Table S2. Costs included in the cost-effectiveness model (Euros, 2019)

	Value (95% CI)	Distribution	Reference
**Event costs**			
Recurrent VTE			
Symptomatic PE	€4,717 (€2,364 – €7,868)	Gamma	[1]
Incidental PE	€0	Fixed	Assumption
DVT	€663 (€464 – €862)	Gamma	[1]
Fatal recurrent VTE [a]	€4,717 (€2,364 – €7,868)	Gamma	[1]
ICH acute care costs	€22,769 (€11,644 – €31,175)	Gamma	[2]
ICH long-term costs (monthly)	€637 (€319 – €1,063)	Gamma	[1]
Non-ICH MB	€10,685 (€5,356 – €17,824)	Gamma	[1]
Fatal MB	€10,685 (€5,356 – €17,824)	Gamma	[1]
CRNMB	€274 (€137 – €457)	Gamma	[1]
PTS	€1,431 (€717 – €2,387)	Gamma	[1]
CTEPH acute care costs	€7,843 (€3,931 – €16,433)	Gamma	[1]
CTEPH long-term costs (monthly)	€89 (€45 – €149)	Gamma	[1]
**Treatment costs**			
Drug cost (daily)			
LMWH [b]	€9.93	Fixed	[3]
Rivaroxaban 15 mg	€4.58	Fixed	[3]
Rivaroxaban 20 mg	€2.29	Fixed	[3]
Treatment duration (days)			
LMWH	183 (137 – 228)	Gamma	[4]
Rivaroxaban 15 mg	21 (16 – 26)	Gamma	[4]
Rivaroxaban 20 mg	162 (121 – 202)	Gamma	[4]
LMWH administration costs			
Costs for home caregiver (per hour)	€59.34 (€44.51 – €74.18)	Gamma	[5]
Duration of at home administration (hour)	0.25 (0.19 – 0.31)	Gamma	Assumption
Hospitalisation duration PE (days) [c]	6.6 (5.0 – 8.3)	Gamma	[6]
Renal monitoring [c]	€1.64 (€1.23 – €2.05)	Gamma	[7]
**Indirect costs**			
Travel costs			
Cost per km	€0.20 (€0.15 – €0.25)	Gamma	[8]
Distance to hospital (km)	7	Fixed	[8]
Distance to GP (km)	1.1	Fixed	[8]
Informal care costs			
PE	€1,515 (€1,136 – €1,894)	Gamma	[5,9]
DVT	€233 (€175 – €291)	Gamma	[5,9]
ICH (acute informal care costs)	€1,515 (€1,136 – €1,894)	Gamma	[5,9]
ICH (long-term informal care costs, monthly)	€626 (€470 – €783)	Gamma	[10]
Non-ICH MB	€758 (€568 – €947)	Gamma	[5,9]
CRNMB	€117 (€87 – €146)	Gamma	[5,9]

Abbreviations: CI, confidence interval; CRNMB, clinically relevant non-major bleeding; CTEPH, chronic thromboembolic pulmonary hypertension; DVT, deep vein thrombosis; GP, general practitioner; ICH, intracranial haemorrhage; LMWH, low-molecular weight heparin; MB, major bleeding; PE, pulmonary embolism; PTS, post-thrombotic syndrome; VTE, venous thromboembolism

[a] Assumed to be equal to the costs of non-fatal PE

[b] *Based on an average weight between 69 and 82 kg.*

[c] *Based on DRG code 070419 and only taken into account for rivaroxaban treated patients*

References

1. Heisen M, Treur MJ, Heemstra HE, Giesen EBW, Postma MJ. Cost-effectiveness analysis of rivaroxaban for treatment and secondary prevention of venous thromboembolism in the Netherlands. J Med Econ. 2017 Aug 3;20(8):813–24.
2. Baeten S a, van Exel NJ a, Dirks M, Koopmanschap M a, Dippel DW, Niessen LW. Lifetime health effects and medical costs of integrated stroke services - a non-randomized controlled cluster-trial based life table approach. Cost Eff Resour Alloc. 2010;8(1):21.
3. Medicijnkosten.nl. The National Health Care Institute (ZIN) [Internet].
4. Young AM, Marshall A, Thirlwall J, Chapman O, Lokare A, Hill C, et al. Comparison of an Oral Factor Xa Inhibitor With Low Molecular Weight Heparin in Patients With Cancer With Venous Thromboembolism: Results of a Randomized Trial (SELECT-D). J Clin Oncol. 2018;36(20):2017–23.
5. Hakkaart-van Roijen L, van der Linden N, Bouwmans C, Kanters T, Swan Tan S. Kostenhandleiding: Methodologie van kostenonderzoek en referentieprijzen voor economische evaluaties in de gezondheidszorg. Zorginstituut Ned. 2016;1–120.
6. van Bellen B, Bamber L, Correa de Carvalho F, Prins M, Wang M, Lensing AWA. Reduction in the length of stay with rivaroxaban as a single-drug regimen for the treatment of deep vein thrombosis and pulmonary embolism. Curr Med Res Opin. 2014;30(5):829–37.
7. Dutch Health Authorities. NZa zorgproductapplicatie. Declaration code: 070419. [Internet]. 2018.
8. Roijen LH, Linden N van der, Bouwmans C, Kanters T, Tan SS. Dutch manual for costing studies in health care. Diemen; 2015.
9. de Klerk M, de Boer A, Plaisier I, Schyns P, Kooiker S. Informele hulp: wie doet er wat? - Rapport - SCP [Internet]. 2015-35. 2015.
10. van den Berg B, Brouwer W, van Exel J, Koopmanschap M, van den Bos GAM, Rutten F. Economic valuation of informal care: lessons from the application of the opportunity costs and proxy good methods. Soc Sci Med. 2006;62(4):835–45.

Supplementary data file – Table S3

Manuscript title: Cost-effectiveness analysis and budget impact of rivaroxaban in cancer patients at risk of recurrent venous thromboembolism

Table S3. Utility values included in the cost-effectiveness model

	Value (95% CI)	Distribution	Reference
**Utilities**			
Index VTE			
0–1 month	0.565 (0.501 – 0.620)	Beta	[1]
1–2 months	0.655 (0.585 – 0.713)	Beta	[1]
2–3 months	0.674 (0.606 – 0.729)	Beta	[1]
3–4 months	0.698 (0.635 – 0.750)	Beta	[1]
4–5 months	0.707 (0.645 – 0.758)	Beta	[1]
Baseline utility 6 months after index VTE	0.715 (0.646 – 0.770)	Beta	[1]
Recurrent VTE			
DVT	0.605 (0.514 – 0.678)	Beta	[1]
Non-fatal symptomatic PE	0.621 (0.477 – 0.725)	Beta	[1]
Non-fatal incidental PE	0.664 (0.615 – 0.707)	Beta	[1]
Non-ICH MB	0.593 (0.461 – 0.693)	Beta	[1]
CRNMB	0.622 (0.568 – 0.669)	Beta	[1]
**Utility decrements**			
Recurrent VTE within first six months after index VTE			
DVT	0.040 (0.000 – 0.158)	Beta	[1]
Symptomatic PE	0.024 (0.000 – 0.195)	Beta	[1]
Incidental PE	0.189 (0.021 – 0.404)	Beta	[1]
ICH	0.380 (0.285 – 0.475)	Beta	[2]
Severe PTS (<6 months after diagnosis)	0.186 (0.090 – 0.280)	Beta	[1]
Severe PTS (>6 months after diagnosis)	0.070 (0.053 – 0.088)	Beta	[2]
CTEPH			
0-1 year	0.194 (0.071 – 0.303)	Beta	[3]
1–4 years	0.109 (0.000 – 0.244)	Beta	[3]
4–5 years	0.079 (0.000 – 0.277)	Beta	[3]

Abbreviations: CI, confidence interval; CRNMB, clinically relevant non-major bleeding; CTEPH, chronic thromboembolic pulmonary hypertension; DVT, deep vein thrombosis; ICH, intracranial haemorrhage; MB, major bleeding; PE, pulmonary embolism; PTS, post-thrombotic syndrome; VTE, venous thromboembolism

References

1. Lloyd AJ, Dewilde S, Noble S, Reimer E, Lee AYY. What Impact Does Venous Thromboembolism and Bleeding Have on Cancer Patients' Quality of Life? Value Heal. 2018;21(4):449–55.
2. Stevanović J, de Jong LA, Kappelhoff BS, Dvortsin EP, Voorhaar M, Postma MJ. Dabigatran for the Treatment and Secondary Prevention of Venous Thromboembolism; A Cost-Effectiveness Analysis for the Netherlands. PLoS One. 2016;11(10):e0163550.
3. Roman A, Barbera JA, Castillo MJ, Muñoz R, Escribano P. Health-related quality of life in a national cohort of patients with pulmonary arterial hypertension or chronic thromboembolic pulmonary hypertension. Arch Bronconeumol. 2013;49(5):181–8.

Supplementary data file – Figure S1

Manuscript title: Cost-effectiveness analysis and budget impact of rivaroxaban in cancer patients at risk of recurrent venous thromboembolism

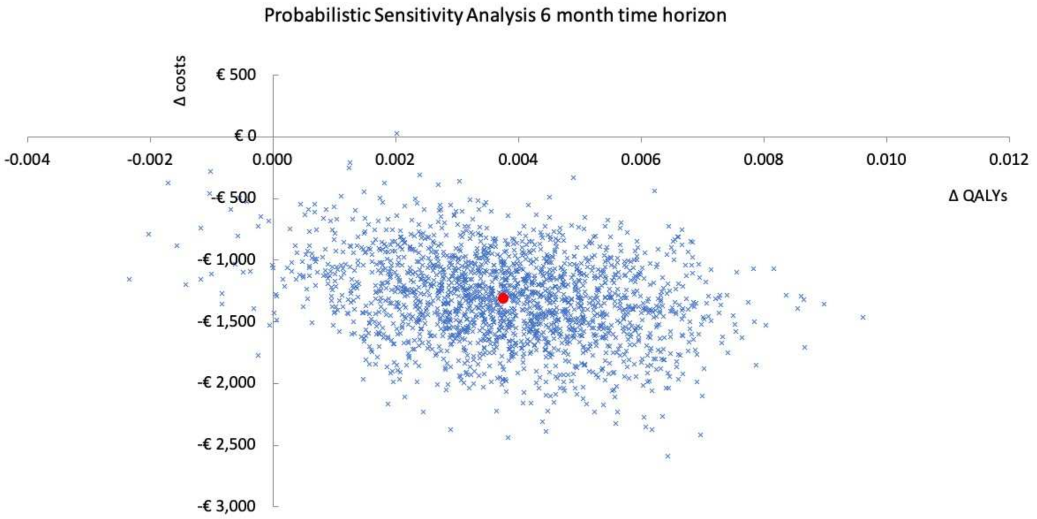

Figure S1. Probabilistic sensitivity analysis with six-month time horizon (scenario 1). *The red mark represents the deterministic incremental cost-effectiveness ratio. Abbreviation: QALY, quality adjusted life-year*

Supplementary data file – Figure S2

Manuscript title: Cost-effectiveness analysis and budget impact of rivaroxaban in cancer patients at risk of recurrent venous thromboembolism

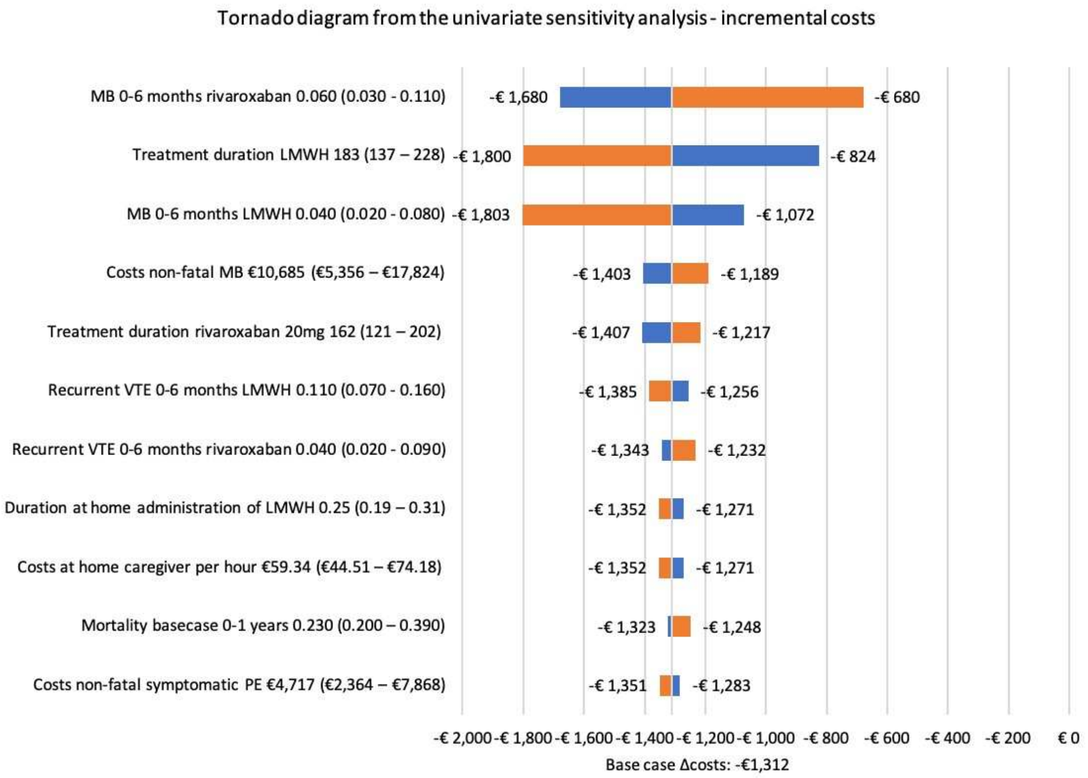

Figure S2. Tornado diagram from the univariate sensitivity analysis for scenario 1 showing the impact of parameters on the incremental costs. *Abbreviations: MB, major bleeding; PE, pulmonary embolism; VTE, venous thromboembolism*

Supplementary data file – Figure S3

Manuscript title: Cost-effectiveness analysis and budget impact of rivaroxaban in cancer patients at risk of recurrent venous thromboembolism

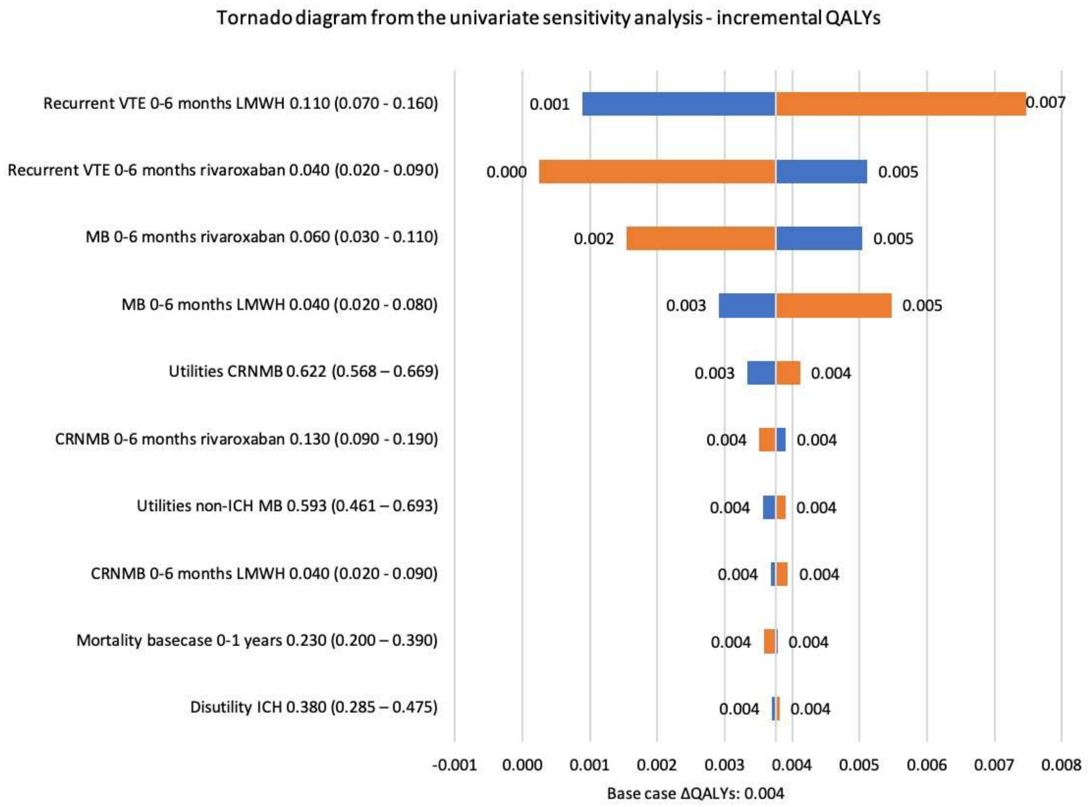

Figure S3. Tornado diagram from the univariate sensitivity analysis for scenario 1 showing the impact of parameters on the incremental QALYs. *Abbreviations: CRNMB, clinically relevant non-major bleeding; ICH, intracranial haemorrhage; MB, major bleeding; PE, pulmonary embolism; VTE, venous thromboembolism*
