# Supplementary material for: Cost-effectiveness analysis and budget impact of rivaroxaban compared with dalteparin in patients with cancer at risk of recurrent venous thromboembolism
Source: BMJ Open. 2020 Nov 19;10(11):e039057. doi: 10.1136/bmjopen-2020-039057 (PMC7678368; doi:10.1136/bmjopen-2020-039057)
Supplement: Author's manuscript [file bmjopen-2020-039057.draft_revisions.pdf]

BMJ Open is committed to open peer review. As part of this commitment we make the peer review history of every article we publish publicly available.

When an article is published we post the peer reviewers' comments and the authors' responses online. We also post the versions of the paper that were used during peer review. These are the versions that the peer review comments apply to.

The versions of the paper that follow are the versions that were submitted during the peer review process. They are not the versions of record or the final published versions. They should not be cited or distributed as the published version of this manuscript.

BMJ Open is an open access journal and the full, final, typeset and author-corrected version of record of the manuscript is available on our site with no access controls, subscription charges or pay-per-view fees (http://bmjopen.bmj.com).

If you have any questions on BMJ Open's open peer review process please email info.bmjopen@bmj.com

BMJ Open

Cost-effectiveness analysis and budget impact of rivaroxaban in cancer patients at risk of recurrent venous thromboembolism

Journal:	*BMJ Open*
Manuscript ID	bmjopen-2020-039057
Article Type:	Original research
Date Submitted by the Author:	06-Apr-2020
Complete List of Authors:	de Jong, Lisa; University of Groningen, Unit of Pharmacotherapy, -Epidemiology and -Economics<br>van der Velden, Annette; Martini Hospital, Department of Internal Medicine<br>Hulst, Marinus; Martini Hospital, Department of Clinical Pharmacy and Toxicology; University Medical Centre Groningen, Department of Health Sciences<br>Postma, Maarten; University Medical Centre Groningen, Department of Health Sciences; University of Groningen, Department of Economics, Econometrics & Finance
Keywords:	Anticoagulation < HAEMATOLOGY, ONCOLOGY, Thromboembolism < CARDIOLOGY, HEALTH ECONOMICS

SCHOLARONE™
Manuscripts

I, the Submitting Author has the right to grant and does grant on behalf of all authors of the Work (as defined in the below author licence), an exclusive licence and/or a non-exclusive licence for contributions from authors who are: i) UK Crown employees; ii) where BMJ has agreed a CC-BY licence shall apply, and/or iii) in accordance with the terms applicable for US Federal Government officers or employees acting as part of their official duties; on a worldwide, perpetual, irrevocable, royalty-free basis to BMJ Publishing Group Ltd ("BMJ") its licensees and where the relevant Journal is co-owned by BMJ to the co-owners of the Journal, to publish the Work in this journal and any other BMJ products and to exploit all rights, as set out in our licence.

The Submitting Author accepts and understands that any supply made under these terms is made by BMJ to the Submitting Author unless you are acting as an employee on behalf of your employer or a postgraduate student of an affiliated institution which is paying any applicable article publishing charge ("APC") for Open Access articles. Where the Submitting Author wishes to make the Work available on an Open Access basis (and intends to pay the relevant APC), the terms of reuse of such Open Access shall be governed by a Creative Commons licence – details of these licences and which Creative Commons licence will apply to this Work are set out in our licence referred to above.

Other than as permitted in any relevant BMJ Author's Self Archiving Policies, I confirm this Work has not been accepted for publication elsewhere, is not being considered for publication elsewhere and does not duplicate material already published. I confirm all authors consent to publication of this Work and authorise the granting of this licence.

For peer review only

Cost-effectiveness analysis and budget impact of rivaroxaban in cancer patients at risk of recurrent venous thromboembolism

Short title: Economic evaluation of rivaroxaban in cancer patients

Authors:

Lisa A. de Jong[1], Annette W.G. van der Velden[2], Marinus van Hulst[3,4]*, Maarten J. Postma[4,5]*

Affiliations:

[1] Unit of PharmacoTherapy, -Epidemiology & -Economics, University of Groningen, Groningen Research Institute of Pharmacy (GRIP), Antonius Deusinglaan 1, Groningen, the Netherlands

[2] Department of Internal Medicine, Martini Hospital, Van Swietenplein 1, Groningen, The Netherlands

[3] Department of Clinical Pharmacy and Toxicology, Martini Hospital, Van Swietenplein 1, Groningen, The Netherlands

[4] Department of Health Sciences, University of Groningen, University Medical Center Groningen, Hanzeplein 1, Groningen, The Netherlands

[5] Department of Economics, Econometrics & Finance, University of Groningen, Faculty of Economics & Business, Nettelbosje 2, Groningen, The Netherlands

*Shared last author

Corresponding author:

Lisa A de Jong

E-mail: l.a.de.jong@rug.nl

Phone: +31630048956

ORCID ID: 0000-0001-8814-0670

For peer review only - http://bmjopen.bmj.com/site/about/guidelines.xhtml

Abstract

Objectives: We aim to calculate the cost-effectiveness and budget impact of rivaroxaban compared to low molecular weight heparin (LMWH) in cancer patients at risk of recurrent venous thromboembolism (VTE).

Setting: The analyses were performed for the Dutch healthcare setting. We built a Markov model to calculate the cost-effectiveness from a societal perspective over a five-year time horizon.

Participants: A hypothetical cohort of 1,000 cancer patients with VTE entered the model with baseline characteristics based on the SELECT-D trial.

Intervention: Six months treatment with rivaroxaban (15 mg twice daily for first three weeks followed by 20 mg once daily) was compared to six months treatment with LMWH dalteparin (200 IU/kg daily during month one followed by 150 IU/kg daily).

Primary and secondary outcome measures: The primary outcome of the cost-effectiveness analysis was the incremental cost-effectiveness ratio (ICER). The robustness of the model was evaluated in probabilistic and univariate sensitivity analyses. A budget impact analysis was performed to calculate the total annual financial consequences.

Results: In the base case and all scenarios, rivaroxaban appeared to be cost-saving while also increasing the patient's health, resulting in dominant ICERs. In the probabilistic sensitivity analysis, 64.1% and 97.3% of the simulations were cost-saving and more effective for a five year and six month time horizon, respectively. Rivaroxaban can save up to €9,834,144 in approximately 8,000 cancer patients with VTE per year compared to LMWH.

Conclusions: Treatment with rivaroxaban is dominant over LMWH in cancer patients at risk for recurrent VTE in the Netherlands. The use of rivaroxaban instead of LMWH can save almost ten million euros per year, primarily driven by the difference in drug costs. Since treatment with rivaroxaban is cost-saving and less invasive, we feel that many cancer patients can benefit from direct oral anticoagulant treatment.

Strengths and limitations of this study

- This analysis includes both cost-effectiveness and budget impact analyses; this way we present the economic impact of the treatment of cancer patients with rivaroxaban on a patient level as well as on a population level.
- To reflect clinical practice, tunnel states were used to model the occurrence of time-dependent events, such as recurrent VTE and bleeding.
- Various additional scenarios were evaluated to show the effect of different assumptions and clinical situations.
- Based on the design of the SELECT-D trial, we assumed a six month treatment duration for all patients, while in clinical practice the treatment duration may vary between patients.
- Although trials have recently shown that apixaban and rivaroxaban are also effective as a primary prophylaxis of VTE in cancer patients compared to a placebo, this study focuses just on the secondary prevention of VTE in cancer patients.

Funding statement: This work was supported by Bayer Pharma.

Competing interests: LA De Jong, M van Hulst and AWG van der Velden declare that they have no competing interest with relation to subject. Postma MJ has received research grants from various

For peer review only - http://bmjopen.bmj.com/site/about/guidelines.xhtml

pharmaceutical companies, including but not limiting to Bayer, Pfizer, Bristol-Myers Squibb, GSK, Roche and Novartis.

For peer review only

For peer review only - http://bmjopen.bmj.com/site/about/guidelines.xhtml

Introduction

Venous thromboembolism (VTE), comprising both pulmonary embolism (PE) and deep vein thrombosis (DVT), is a major challenge in patients with cancer [1]. In addition to the characteristics of the cancer itself, cancer therapy (chemotherapy and cancer surgery) has effects on the patient's coagulation system and therefore increases the risk of VTE and bleeding [2–4]. VTE in cancer patients can cause unnecessary hospitalizations, interruption or postponement of cancer treatment, and increased mortality, leading to decreased quality of life and increased costs.

VTE is treated with anticoagulation therapy, and this is continued as prophylaxis over a longer period because of the high risk of recurrence during the first months after the initial VTE [5]. Vitamin K antagonists (VKAs) or direct oral anticoagulants (DOACs) are indicated for the treatment and prevention of VTE in the general population [6]. However, guidelines recommend therapeutic treatment with a daily subcutaneous injection of low molecular weight heparin (LMWH, e.g., dalteparin) in cancer patients [4,7–9].

The guidelines recommend against the use of VKAs in cancer patients because of potential drug interactions, liver dysfunction, and malnutrition, all of which lead to fluctuations of the international normalized ratio (INR) and could result in negative patient outcomes [4]. Moreover, trials in cancer patients with VTE have shown that LMWH is more effective in the prevention of recurrent VTE compared to VKA, without increasing bleeding risk [10–12].

DOACs have a more beneficial efficacy/safety ratio, do not require routine measurements of the INR, and show fewer food-drug and drug-drug interactions compared to VKAs [4,13]. Therefore, it is suggested that, unlike VKAs, DOACs can play an important role in the VTE treatment paradigm for cancer patients. However, the use of DOACs is not yet recommended by oncology guidelines because of the limited available data in cancer patients with VTE [4,9].

The SELECT-D is a multicenter, randomized, clinical pilot trial in the UK; it is a head-to-head comparison of rivaroxaban and the LMWH dalteparin in 406 patients with active cancer who had experienced a symptomatic PE, incidental PE, or symptomatic DVT [14]. Patients with upper gastrointestinal (GI) cancer were excluded because of a high bleeding risk. They found that rivaroxaban reduces the recurrence of VTE (six month cumulative VTE recurrence rate: 4% versus 11%) at the cost of an increased risk of bleeding (six month cumulative major bleeding [MB] rate: 6% versus 4%; six month cumulative clinically relevant non-major bleeding [CRNMB] rate: 13% versus 4%) compared to dalteparin. These results were comparable to those of a large retrospective study by Streiff et al. [15].

Based on the results of these studies and the fact that DOACs can be orally administered (unlike the subcutaneously injected LMWHs), an increase in the use of DOACs for VTE in cancer patients might be expected. Since the introduction of DOACs there has been an ongoing discussion about the economic impact of these drugs. By designing an economic model based on the SELECT-D trial, we aim to evaluate the cost-effectiveness and budget impact of rivaroxaban compared to LMWH in cancer patients at risk of recurrent venous thromboembolism in the Netherlands.

Methods

The economic model comparing rivaroxaban to LMWH was designed based on the SELECT-D trial [14], since this study presented the most comprehensive results reflecting recurrent VTE and bleeding complications per event type (symptomatic PE, incidental PE, and DVT) or severity (MB and CRNMB). The primary outcome of the cost-effectiveness analysis is the incremental cost-effectiveness ratio

For peer review only

For peer review only - http://bmjopen.bmj.com/site/about/guidelines.xhtml

(ICER); this is calculated by dividing the incremental costs by the incremental health effects, expressed in quality adjusted life-years (QALYs). In accordance with Dutch costing guidelines for economic evaluations in healthcare, the ICER was calculated from a societal perspective, which incorporates direct as well as indirect costs both inside and outside the healthcare sector [16]. We performed sensitivity and scenario analyses to test the robustness of the model. Additionally, we conducted a budget impact analysis to reflect the annual financial consequences of the use of rivaroxaban in the Netherlands.

Model outline

We developed a decision-tree-based Markov model to calculate the ICER. Figure 1 shows a schematic representation of the model, with the disease course being represented by separate health states. A hypothetical cohort of 1,000 cancer patients with VTE entered the model with incidental PE, symptomatic PE, or DVT, represented by the 'index VTE' health state. Incidental PEs are non-symptomatic PEs that are incidentally found during tumour imaging. According to guidelines, patients with incidental PE should be treated identically to those with symptomatic PE [8,9]. Patient characteristics were based on the SELECT-D trial (Table 1) [14]. The SELECT-D population is representative for the Dutch population, based on age, tumour type, and gender distribution [17]. Patients move through various health states in the model during the follow-up time of five years; five years was used because overall survival was assumed to be low after five years since the majority (58%) of the SELECT-D trial population had metastatic cancer [14]. We included the following health states in our model (see legend of Figure 1 for abbreviations): 'recurrent incidental PE', 'recurrent symptomatic PE', 'fatal recurrent VTE', 'recurrent DVT', 'ICH', 'non-ICH MB', 'fatal MB', 'CRNMB', 'death by any cause', and 'no event'. Patients were assumed to remain in these states for one cycle, after which they moved back to the 'index VTE' state or the chronic, debilitating 'post-ICH' state, in which they remained until death without being at risk for any further complications. Tunnel states (one month post-VTE, two months post-VTE, …, 60 months post VTE) were used to implement time-dependency, with future transitions, costs, and health-related quality of life dependent on how long the patient has gone without a recurrent VTE event [18]. The chronic complications post-thrombotic syndrome (PTS) and chronic thromboembolic pulmonary hypertension (CTEPH) were modelled in the background. Only severe PTS was modelled, since the costs of minor PTS are considered negligible. For these chronic complications we also used tunnel states since the risks of PTS and CTEPH were also time-dependent.

Figure 1. Model outline. All patients enter the model in the 'Index VTE' state and move to other states upon the occurrence of one of the following events: recurrent incidental PE, recurrent symptomatic PE, fatal recurrent VTE, recurrent DVT, ICH, non-ICH MB, fatal MB, CRNMB, or death by any cause. The triangles represent the health state a patient will enter after an event. The blue squares are permanent states, in which a patient will remain until death while not being at risk for other events. The red squares represent a transient state: the patient will re-enter the model in the 'Index VTE' state.

Abbreviations: CRNMB, clinically relevant non-major bleeding; DVT, deep vein thrombosis; ICH, intracranial haemorrhage; MB, major bleeding; PE, pulmonary embolism; VTE, venous thromboembolism

Table 1. Patient characteristics of the hypothetical cohort of 1,000 cancer patients at risk of recurrent VTE.

For peer review only - http://bmjopen.bmj.com/site/about/guidelines.xhtml

Unit	Value	Reference
Age (years)	67	[14]
Proportion male	53%	[14]
BMI (kg/m^2)	25.6	[14]
Type of cancer		
Early or locally advanced cancer	39%	[14]
Metastatic cancer	58%	[14]
Haematologic malignancy	2%	[14]
Distribution of PE and DVT		
% index VTE that is symptomatic PE	20%	[14]
% index VTE that is incidental PE	53%	[14]
% index VTE that is DVT	27%	[14]

Abbreviations: BMI, body mass index; DVT, deep vein thrombosis; LMWH, low-molecular weight heparin; PE, pulmonary embolism; VTE, venous thromboembolism

Transition probabilities

Transition probabilities were used to calculate the number of patients in each state per cycle. The cycle length was one month. Table S1 summarizes all event rates presented in six-month risks (transition probabilities). Event rates of recurrent VTE, MB, and CRNMB in the first six months of treatment were based on the SELECT-D trial [14]. If the patient did not experience a recurrent event during this period, anticoagulation treatment was discontinued. Recurrent VTE rates after treatment discontinuation were based on a retrospective study in active-cancer patients experiencing a VTE [5]. Upon the occurrence of a non-fatal recurrent VTE, patients were assigned to another six months treatment, with corresponding event rates. Bleeding risks after treatment discontinuation were based on the outcomes of the cancer population of the HOKUSAI VTE Cancer trial (which followed patients after edoxaban discontinuation for an additional six months) because this data is not reported for the SELECT-D trial [19]. The HOKUSAI VTE Cancer trial was also used to determine the distribution of ICH, non-ICH, and fatal bleeding. The distributions of the types of VTE and MB were calculated based on the total number of events and assumed to be treatment-independent, since the total number of events was low. The distributions of the types of VTE event were based on the number of recurrent VTE events in the lower extremities and pulmonary embolisms— other locations of VTE events (brachial, subclavian, jugular, renal plus inferior vena cava, or the extrahepatic vein) were excluded [14]. Mortality rates (death by any cause) were based on Dutch cancer mortality data from the Netherlands Cancer Registry [20]. In the sensitivity analysis, all transition probabilities were varied over beta distributions. For percentages of the type of recurrent VTE and MB, a Dirichlet distribution was used in the sensitivity analysis [18].

Costs

All cost parameters are presented in Table S2. Event-related healthcare costs were based on a previous Dutch cost-effectiveness study for rivaroxaban in the general VTE population [21]. Costs of fatal recurrent VTE were assumed to be similar to those of non-fatal symptomatic PE. We assumed no event-related healthcare costs for patients with incidental PE. Costs for ICH and CTEPH consisted of acute care costs during the first month after diagnosis, followed by long-term care costs until the patient moved to the 'death' state. Costs of a fatal MB were assumed to be equal to those of non-fatal non-ICH MB.

For peer review only - http://bmjopen.bmj.com/site/about/guidelines.xhtml

Drug costs were retrieved from the national medication costs database [22]. For rivaroxaban these costs were based on 15 mg twice daily for three weeks followed by 20 mg once daily. Drug costs of LMWH dalteparin were based on 200 IU/kg daily during month one followed by 150 IU/kg daily in months two to six [14,23]. Based on an average body mass index of 25.6 from the SELECT-D trial and an average height of 1.72 m for the Dutch population, we calculated that the average weight was between 69 and 82 kg, which corresponds with a dose of 15,000 IU daily during month one followed by 12,500 IU daily in months two to six [14,24]. Rivaroxaban users were assumed to require an annual check-up of their renal function [13]. We assumed no administration costs for LWMH because most patients can perform the injection themselves or through their proxies.

Informal care costs were only applied to patients with early or locally advanced cancer (39%), since patients with metastatic cancer or haematologic malignancies often already have home care or informal care. Based on a previously published report on informal care in the Netherlands, we made a distinction between intensive (26 hours per week) and non-intensive (8 hours per week) informal care [25]. This was multiplied by the average duration and tariff for informal care, obtained from the Dutch cost manual [16]. To prevent double counting, we did not include informal care costs for the chronic complications. Travel costs were taken into account for renal monitoring visits and upon the occurrence of a DVT or CRNMB. Considering the burden of cancer and the average age of 67 years (which is the Dutch retirement age), productivity losses were assumed to be negligible. Costs related to forgone leisure activity were not taken into account since there is no data available on the impact of a VTE or bleeding on leisure losses in cancer patients.

Costs were discounted at an annual rate of 4% [16]. In the sensitivity analysis, the costs were varied with gamma distributions corresponding to the 95% confidence interval (CI), as indicated in Table S2 [18].

Utilities

Utility scores were derived from a sub-analysis from the CATCH study assessing the EQ-5D scores associated with VTE and recurrent VTE in cancer patients (Table S3) [26]. The CATCH study assessed the effectiveness of six months of treatment with tinzaparin versus warfarin for the treatment of acute venous thromboembolism in patients with active cancer; It was chosen because it aligns well with our population and events of interest. Utility decrements for CTEPH were based on a study assessing EQ-5D VAS scores in CTEPH patients more than 7 years after initial diagnosis [27]. Utility decrements for ICH and long-term PTS (> six months after diagnosis) were obtained from a previous cost-effectiveness study [28]. Utilities were discounted at 1.5% according to Dutch guidelines [16]. In the sensitivity analyses, utility scores were varied over their 95% CI with a beta distribution [18].

Sensitivity analysis

Sensitivity analyses were conducted to check the robustness of the model. In the probabilistic sensitivity analysis, all input parameters were varied simultaneously over their 95% CI. If the 95% CI was unavailable and calculating the 95% CI based on the number of events was not possible, the 95% CI was calculated based on a 25% standard error. The ICER was calculated with 2,000 iterations and plotted in a cost-effectiveness plane. A univariate sensitivity analysis was conducted to show the

For peer review only - http://bmjopen.bmj.com/site/about/guidelines.xhtml

influence of an individual parameter on the ICER. The fifteen most influencing parameters were presented in a tornado diagram.

Scenario analysis

We conducted several scenario analyses to show the effect on the outcomes of different (clinical) situations. In the Netherlands, guidelines advise to calculate the ICER from a societal perspective, while in countries such as the UK or Belgium, the healthcare payer's perspective is preferred. To make results comparable to other countries we also calculated the base case ICER from a healthcare payer's perspective, by excluding the indirect costs (scenario 1). The costs of LMWH vary with the patient's weight. For the base case analysis we assumed an average weight between 69 and 82 kg. In scenarios 2 and 3 we calculated the base case ICER with the costs of dalteparin based on weight categories of 57–68 kg (12,500 IE daily during month one followed by 10,000 IE daily in month two to six) and 83–98 kg (18,000 IE daily during month one followed by 15,000 IE daily in month two to six), respectively. The follow-up period of the SELECT-D trial was six months; therefore, outcomes beyond six months had to be based on other publications. In the scenario 4 analysis we calculated the ICER with a time horizon of six months. Scenario 5 was similar to scenario 4, except for the treatment period which was based on a study of Streiff et al., who—comparable to SELECT-D—compared rivaroxaban to LMWH for the prevention of recurrent VTE in cancer patients [15]. They found an average treatment duration of one month and three months for LMWH and rivaroxaban, respectively. In scenario 6 we assessed the effect of using drug-specific distributions of the types of VTE and MB based on the results of the SELECT-D and HOKUSAI VTE Cancer trials [14,19].

Budget impact

A budget impact analysis was conducted to estimate the total financial consequences of the implementation of rivaroxaban for the treatment and prevention of VTE in cancer patients within the Dutch healthcare setting. The budget impact was calculated from a societal perspective over a one-year time horizon. Results from the first year of the cost-effectiveness analysis were multiplied by the annual number of cancer patients with VTE in the Netherlands. This incidence and the total number of Dutch cancer patients were used to calculate the yearly number of cancer patients with VTE. The Netherlands Cancer Registry estimated a total of 579,781 cancer patients in 2017 [29]. The incidence of VTE in cancer patients was 13.9 per 1,000 person-years, based on a cohort study of linked UK databases [30]. The outcome of the budget impact analysis was presented as the total budget impact per year, including a subdivision of the costs per type (event-related costs, treatment costs and indirect costs) and corresponding 95% CIs derived from PSA.

Results

Cost-effectiveness analysis

Table 2 represents the deterministic results of the base case and scenario analyses. In each scenario, rivaroxaban appeared to be cost-saving compared to LMWH where the patient's health was comparable (incremental QALYs of 0.012 in the base case analysis). In the base case analysis, rivaroxaban saved €1,310 per patient compared to LMWH. There was increased cost savings compared

For peer review only - http://bmjopen.bmj.com/site/about/guidelines.xhtml

For peer review only

to the societal perspective when calculated from a healthcare payer's perspective (scenario 1). In scenario 2 and 3 we assessed the effect of variations in the patient's weight (and thus LMWH dosing) on the ICER. Compared to the base case analysis, there was decreased cost savings with a lower LMWH dose and increased cost savings with a higher LMWH dose, both still resulting in dominant ICERs. The scenario calculating the cost-effectiveness over six months resulted in cost savings of €1,147 per patient (scenario 4). When comparing three months of rivaroxaban treatment to one month of LMWH treatment, we found incremental QALYs of 0.017 and cost savings of €327 per patient (scenario 5). We assessed the effect of using drug-specific distributions of the types of VTE and MB, resulting in cost savings of €1,652 and incremental QALYs of 0.038 (scenario 6).

The number of events and the corresponding average costs per patient are presented in Table 3. Rivaroxaban is associated with a lower number of recurrent VTE events, preventing on average €131 and €108 in costs per patient over five years and over six months, respectively. On the other hand, rivaroxaban causes more bleeding events, especially in the treatment period. ICH, non-ICH MB, and CTEPH have the highest event-related costs. Treatment costs are higher for LMWH compared to rivaroxaban, with incremental costs of €1,559 and €1,306 in the five-year and the six-month time horizon, respectively. The indirect costs for rivaroxaban were higher compared to LMWH resulting in a difference of €23 and €2 for the five-year and the six-month time horizon, respectively.

Table 2. Deterministic results per patient of the base case and scenario analyses in a cohort of 1,000 cancer patients.

	Costs	QALYs	Δ Costs	Δ QALYs	ICER
**Base case analysis** - 5 year time horizon from societal perspective					
Rivaroxaban	€3,127	2.460	-€1,310	0.012	Dominant
LMWH	€4,437	2.448			
**Scenario 1** – base case analysis from healthcare payer's perspective					
Rivaroxaban	€2,942	2.460	-€1,334	0.012	Dominant
LMWH	€4,276	2.448			
**Scenario 2** – base case analysis with LMWH dose of 12,500 IU					
Rivaroxaban	€3,127	2.460	-€913	0.012	Dominant
LMWH	€4,040	2.448			
**Scenario 3** – base case analysis with LMWH dose of 18,000 IU					
Rivaroxaban	€3,127	2.460	-€1,732	0.012	Dominant
LMWH	€4,858	2.448			
**Scenario 4** – 6 month time horizon from societal perspective					
Rivaroxaban	€1,358	0.304	-€1,147	0.004	Dominant
LMWH	€2,505	0.300			
**Scenario 5** – scenario 4 with treatment duration based on Streiff et al.					
Rivaroxaban	€1,285	0.289	-€327	0.017	Dominant
LMWH	€1,611	0.272			
**Scenario 6** – base case analysis using drug-specific distributions for the types of VTE and MB					
Rivaroxaban	€3,047	2.463	-€1,652	0.038	Dominant
LMWH	€4,699	2.426			

Abbreviations: ICER, incremental cost-effectiveness ratio; IU, international units; LMWH, low molecular weight heparin; QALY, quality adjusted life-years

Table 3. Number of events and costs per event per patient in a cohort of 1,000 cancer patients.

Base case (5 year time horizon)			
	**Rivaroxaban**	**LMWH**	**Incremental**

For peer review only - http://bmjopen.bmj.com/site/about/guidelines.xhtml

	Number of events	Costs per patient	Number of events	Costs per patient	Number of events	Costs per patient
**Event costs**						
Recurrent VTE	191	€311.85	275	€442.92	-84	-€131
Non-fatal symptomatic recurrent PE	33	€168.36	48	€239.13	-15	-€71
Non-fatal incidental recurrent PE	58	-	84	-	-26	
Non-fatal recurrent DVT	83	€59.31	120	€84.23	-37	-€25
Fatal recurrent VTE	17	€84.18	24	€119.56	-7	-€35
ICH	11	€550.70	9	€438.40	2	€112
Non-ICH MB	98	€1,106.87	79	€902.47	19	€204
Fatal MB	5	€51.48	4	€41.98	1	€10
CRNMB	197	€56.28	92	€26.93	105	€29
PTS	61	€92.72	61	€92.37	0	€0
CTEPH	20	€223.79	20	€222.83	0	€1
**Treatment costs**		€548.83		€2,108.33		-€1,559
**Indirect costs**		€184.22		€160.77		€23
**Scenario 4 (6 month time horizon)**						
	**Rivaroxaban**		**LMWH**		**Incremental**	
	Number of events	Costs per patient	Number of events	Costs per patient	Number of events	Costs per patient
**Event costs**						
Recurrent VTE	38	€58.95	109	€166.96	-70	-€108
Non-fatal symptomatic recurrent PE	7	€31.82	19	€90.14	-12	-€58
Non-fatal incidental recurrent PE	12	-	33	-	-21	-
Non-fatal recurrent DVT	17	€11.21	47	€31.75	-31	-€21
Fatal recurrent VTE	3	€15.91	9	€45.07	-6	-€29
ICH	6	€142.82	4	€94.25	2	€49
Non-ICH MB	50	€539.38	33	€355.95	17	€183
Fatal MB	2	€25.09	2	€16.56	1	€9
CRNMB	130	€35.99	38	€10.62	91	€25
PTS	14	€20.59	14	€20.56	0	€0
CTEPH	3	€21.96	3	€21.93	0	€0
**Treatment costs**		€479.40		€1,785.45		-€1,306
**Indirect costs**		€33.79		€32.22		€2

Abbreviations: CRNMB, clinically relevant non-major bleeding; CTEPH, chronic thromboembolic pulmonary hypertension; DVT, deep vein thrombosis; ICH, intracranial haemorrhage; LMWH, low molecular weight heparin; MB, major bleeding; PE, pulmonary embolism; PTS, post thrombotic syndrome; VTE, venous thromboembolism

In the probabilistic sensitivity analysis we assessed the robustness of the model over a five-year time horizon (base case) and a six-month time horizon (scenario 4). The results are presented in cost-effectiveness planes in Figure 2 and Figure 3. Of the 2,000 iterations of the base case ICER, 77.0% were located in the south-eastern quadrant and 22.8% are considered cost-saving but less effective compared to LMWH. In scenario 4, 98.7% of the calculations were located in the south-eastern quadrant and in 1.2% rivaroxaban is considered cost-saving but less effective compared to LMWH.

The influence of the individual input parameters on the base case incremental costs and QALYs are analysed in the univariate sensitivity analysis. The tornado diagrams (Figure 4 and Figure 5) present the 15 input parameters with the highest impact. The risk of MB for both rivaroxaban and LMWH, treatment duration of LMWH, and recurrent VTE risks during the first six months after a VTE had the highest influence on the incremental costs. Similarly, the risk of MB and recurrent VTE in the first six months for rivaroxaban and LMWH showed the highest influence on the incremental QALYs.

For peer review only - http://bmjopen.bmj.com/site/about/guidelines.xhtml

For peer review only

Figure 2. Probabilistic sensitivity analysis of the base case with five year time horizon (base case analysis). *Abbreviation: QALY, quality adjusted life-year*

Figure 3. Probabilistic sensitivity analysis with six month time horizon (scenario 4). *Abbreviation: QALY, quality adjusted life-year*

Figure 4. Tornado diagram from the univariate sensitivity analysis for the base case analysis showing the impact of parameters on the incremental costs. *Abbreviations: ICH, intracranial haemorrhage; LMWH, low molecular weight heparin; MB, major bleeding; PE, pulmonary embolism; VTE, venous thromboembolism*

Figure 5. Tornado diagram from the univariate sensitivity analysis for the base case analysis showing the impact of parameters on the incremental QALYs. *Abbreviations: CRNMB, clinically relevant non-major bleeding; ICH, intracranial haemorrhage; LMWH, low molecular weight heparin; MB, major bleeding; VTE, venous thromboembolism*

Budget impact

The results of the budget impact analysis are presented in Table 4. The replacement of LMWH with rivaroxaban can lead to annual cost savings of a maximum of €9,991,357 (€4,419,972–€15,407,533) over approximately 8,000 cancer patients with VTE. A reduction in treatment costs can lead to savings of up to €11.2 million. Event-related costs and indirect costs slightly increase by €1,234,467 (€-2,364,452–€5,177,816) and €27,749 (€-137,276–€189,561), respectively, when LMWHs are replaced by rivaroxaban.

Table 4. Budget impact over one year time in the Netherlands.

Event-related costs	€1,234,467 (€-2,364,452–€5,177,816)
Treatment costs	€-11,253,573 (€-15,363,435–€-7,309,562)
Indirect costs	€27,749 (€-137,276–€189,561)
**Budget impact**	**€-9,991,357 (€-15,407,533–€-4,419,972)**

Discussion

Thrombosis treatment is a challenge in cancer patients. According to the guidelines, LMWHs are the preferred treatment; however, DOACs have recently also been shown to be effective and safe in cancer patients with VTE. We have assessed the cost-effectiveness and budget impact of rivaroxaban in cancer patients based on the SELECT-D trial [14]. We conclude that, in the Netherlands, rivaroxaban is a cost-saving treatment option with a health benefit of 0.012 QALYs per patient over five years compared to LMWH. In a sensitivity analysis our model appeared to be robust.

For peer review only - http://bmjopen.bmj.com/site/about/guidelines.xhtml

For peer review only

In both the cost-effectiveness analysis and budget impact analysis we found that the event-related costs and indirect costs increase with the use of rivaroxaban compared to LMWH. In total, 42 events were prevented over five years; however, MB events were more frequent with rivaroxaban compared to LMWH (11 ICH and 98 non-ICH versus 9 ICH and 79 non-ICH, respectively). MB events are very burdensome and frequently severely disabling, causing high acute care and long-term direct and indirect costs which makes these events very expensive. This might explain why the indirect costs were higher for rivaroxaban than for LMWH. Moreover, there was no data available on leisure activity losses caused by the occurrence of a VTE event in patients who are already burdened with cancer. Therefore, the indirect costs might have been underestimated, possibly leading to lower cost savings results. The indirect costs account for €160 to €185 per patient over five years—approximately 3-5% of the total cost—however, they do not have a major influence on the differences between the two drugs (€23 and €2 for the five-year and 6-month time horizon, respectively). This suggests that, although the indirect costs might have been underestimated, rivaroxaban is still likely to be cost-saving compared to LMWH.

The main driver of the cost savings is the difference in treatment costs. In the cost-effectiveness analysis, we estimated that more than €1,500 per patient over a five-year period can be saved on treatment costs, compared to LMWH. We conservatively assumed no additional costs for training or assistance for administration of the LMWH injection. Moreover, in the scenario analysis we varied the price of dalteparin based on weight. Although the lowest dose (12,500 IU daily during month one followed by 10,000 IU in months two to six based on weight class 57–68 kg) had a lower price, €8.06 versus €9.93, the ICER remained cost-saving. Based on this same weight class, only LMWH nadroparin has a lower price compared to dalteparin. However, since the lower dose of dalteparin is still highly cost-saving, it is expected that compared to LMWHs other than dalteparin, rivaroxaban will also be a cost-saving alternative [22].

In the budget impact analysis, we calculated that rivaroxaban replacing LMWH leads to cost savings of a maximum of €9,991,357 within one year over a total of 8,000 cancer patients. This is the absolute maximum, since it is not possible to treat each patient with rivaroxaban from a clinical perspective. In practice, the market share of rivaroxaban will be lower—despite the fact that there are three other DOACs that could be prescribed—because there are some clinical considerations that should be taken into account. Firstly, although DOACs have far fewer drug interactions than VKAs, it should be noted that rivaroxaban is metabolized by CYP3A4 enzymes [1]. Cancer patients, especially those with haematological cancer, are at high risk for opportunistic and fungal infections, for which they are often treated with CYP3A4 inhibitors or inducers [31]. For this reason, prescription of rivaroxaban for the prevention of recurrent VTE in cancer patients must be done carefully [1]. This interaction does not play a role in LWMH treatment.

Secondly, the balance between the risk of thrombosis and the risk of bleeding should always be a consideration in the prescription of anticoagulants. For example, DOACs are not advised in patients with GI tumours, due to a higher risk of GI bleeding; for this reason they were not included in the SELECT-D trial [14]. Some prediction scores for primary prevention have been developed to predict thrombosis risk in cancer patients, since thrombosis prophylaxis is most effective in patients with an increased VTE risk. Unfortunately, for cancer these scores have still not been shown to reliably identify patients with the highest risk [32]. Predictive scores for bleeding, such as the HAS-BLED score used for atrial fibrillation patients, are also needed.

A third consideration is the oral administration of rivaroxaban. Although it is less burdensome than the LMWH injections, oral administration can be problematic in patients with anorexia and

For peer review only

For peer review only - http://bmjopen.bmj.com/site/about/guidelines.xhtml

vomiting, which is often seen as a side effect in cancer therapy [14]. Moreover, low food intake might influence the metabolism of rivaroxaban resulting in lower bioavailability [33]. Lastly, adherence is always a point of discussion, but since adherence to current guidelines is often low [32], we feel that adherence might even increase due to the more patient-friendly administration.

As with all cost-effectiveness models some assumptions need to be made due to lack of data. We assumed that patients were treated with anticoagulation over six months, which is in line with the guidelines of the Dutch Internist Society (NIV) [7]. Previous studies have shown that adherence to these guidelines is poor [32]. As seen in the study by Streiff et al, in practice, treatment with LMWH is often not six months, presumably due to the fact that LMWH injections are burdensome, there are concerns about the bleeding risk, and the complexity of the treatment of cancer patients [32]. However, this recommended treatment period was also not achieved in many patients treated with rivaroxaban, which resulted in an average duration of three months. We conducted a scenario analysis (scenario 5) to assess this difference in treatment duration. Incremental QALYs increased while still being cost-saving. On the other hand, there are some clinical situations in which the treatment period might be longer than six months: for example, in patients with a recurrent VTE event, patients with an active malignancy, or patients receiving treatment for their malignancy for over more than six months. Moreover, in the Netherlands anticoagulation is often continued after six months of initial treatment in case the cancer is still active. Unfortunately, we were unable to assess the effect of continued anticoagulation treatment due to lack of data. However, since rivaroxaban is associated with cost-saving results during the first six months, it is to be expected that during a longer treatment period the savings and health gains will increase even more compared to LMWH.

In the univariate sensitivity analysis we have shown that the risk of MB and VTE for both rivaroxaban and LMWH have a high influence on the incremental costs and QALYs. In the SELECT-D trial [14], the incidence of symptomatic and fatal PE events was relatively higher in patients treated with rivaroxaban. However, due to low numbers of VTE observed in the SELECT-D trial [14], we calculated the distribution of the type of VTE based on the total number of events and assumed it to be equal for both drugs. This may have led to an overestimation of the effect of rivaroxaban compared to LMWH, since symptomatic and fatal PE events have a higher impact on the costs and the patient's health compared to DVT and incidental PE. On the other hand, we used this same approach to calculate the distributions of the types of MB from the HOKUSAI VTE Cancer trial [19], in which the patients treated with LMWH had relatively more severe MB events compared to the NOAC (ICH: 17.6% versus 6.1%, respectively). This results in an overestimation of the safety of LMWH. We assessed the effect of using drug-specific distributions of the type of VTE and MB in scenario six, showing an increase in incremental cost savings and QALYs compared to the base case analysis. Therefore, we conclude that our approach of using equal distributions of the types of VTE and MB for rivaroxaban and LMWH is conservative.

This study focuses on the secondary prevention of VTE, based on the results of the SELECT-D and, partially, the HOKUSAI VTE Cancer trials. However, the AVERT and CASSINI trials have recently shown that apixaban and rivaroxaban are also effective as a primary prophylaxis of VTE in cancer patients compared to a placebo [34–36]. Based on these two studies, clinicians may consider DOAC prophylaxis in some of their cancer patients [36]. Therefore, future research is needed to assess if DOACs are also cost-effective for the primary prevention of VTE.

For peer review only - http://bmjopen.bmj.com/site/about/guidelines.xhtml

Conclusion

Treatment with rivaroxaban is dominant (cost-saving while slightly increasing the patient's health) over LMWH in cancer patients at risk for recurrent VTE in the Netherlands. The use of rivaroxaban instead of LMWH can save almost ten million euros per year, which is primarily driven by the difference in drug costs. Since treatment with rivaroxaban is cost-saving and less invasive, we feel that many cancer patients can benefit from DOAC treatment. However, with DOAC treatment interactions, oral administration and adherence should be kept in mind.

References

1. Gerotziafas GT, Mahé I, Elalamy I. Therapeutics and Clinical Risk Management Dovepress New orally active anticoagulant agents for the prevention and treatment of venous thromboembolism in cancer patients. Ther Clin Risk Manag. 2014;10:423–36.
2. Falanga A, Marchetti M, Vignoli A. Coagulation and cancer: biological and clinical aspects. J Thromb Haemost. 2013;11(2):223–33.
3. Lechner D, Weltermann A. Chemotherapy-induced thrombosis: a role for microparticles and tissue factor? Semin Thromb Hemost. 2008;34(2):199–203.
4. Mandala M, Falanga A, Roila F, ESMO Guidelines Working Group. Management of venous thromboembolism (VTE) in cancer patients: ESMO Clinical Practice Guidelines. Ann Oncol. 2011;22(Supplement 6):vi85–92.
5. Cohen AT, Katholing A, Rietbrock S, Bamber L, Martinez C. Epidemiology of first and recurrent venous thromboembolism in patients with active cancer. Thromb Haemost. 2017;117(01):57–65.
6. Minister of Health W and S. Letter to the parlement - reimbursement rivaroxaban VTE (Herbeoordeling uitbreiding nadere voorwaarden rivaroxaban (Xarelto®) bij VTE. 2015;
7. Dutch Internist Society (NIV). Guideline Antithrombotic policy [Internet]. Utrecht; 2015.
8. Kearon C, Akl EA, Ornelas J, Blaivas A, Jimenez D, Bounameaux H, et al. Antithrombotic Therapy for VTE Disease: CHEST Guideline and Expert Panel Report. Chest. 2016;149(2):315–52.
9. Lyman GH, Bohlke K, Khorana AA, Kuderer NM, Lee AY, Arcelus JI, et al. Venous Thromboembolism Prophylaxis and Treatment in Patients With Cancer: American Society of Clinical Oncology Clinical Practice Guideline Update 2014. J Clin Oncol. 2015;33(6):654–6.
10. Hull RD, Pineo GF, Brant RF, Mah AF, Burke N, Dear R, et al. Long-term low-molecular-weight heparin versus usual care in proximal-vein thrombosis patients with cancer. Am J Med. 2006 Dec;119(12):1062–72.
11. Lee AYY, Levine MN, Baker RI, Bowden C, Kakkar AK, Prins M, et al. Low-Molecular-Weight Heparin versus a Coumarin for the Prevention of Recurrent Venous Thromboembolism in Patients with Cancer. N Engl J Med. 2003;349(2):146–53.
12. Meyer G, Marjanovic Z, Valcke J, Lorcerie B, Gruel Y, Solal-Celigny P, et al. Comparison of low-molecular-weight heparin and warfarin for the secondary prevention of venous thromboembolism in patients with cancer: a randomized controlled study. Arch Intern Med. 162(15):1729–35.
13. Steffel J, Verhamme P, Potpara TS, Albaladejo P, Antz M, Desteghe L, et al. The 2018 European Heart Rhythm Association Practical Guide on the use of non-vitamin K antagonist oral anticoagulants in patients with atrial fibrillation. Eur Heart J. 2018;
14. Young AM, Marshall A, Thirlwall J, Chapman O, Lokare A, Hill C, et al. Comparison of an Oral Factor Xa Inhibitor With Low Molecular Weight Heparin in Patients With Cancer With Venous Thromboembolism: Results of a Randomized Trial (SELECT-D). J Clin Oncol. 2018;36(20):2017–23.

For peer review only - http://bmjopen.bmj.com/site/about/guidelines.xhtml

15. Streiff MB, Milentijevic D, McCrae K, Yannicelli D, Fortier J, Nelson WW, et al. Effectiveness and safety of anticoagulants for the treatment of venous thromboembolism in patients with cancer. Am J Hematol. 2018;93(5):664–71.
16. Hakkaart-van Roijen L, van der Linden N, Bouwmans C, Kanters T, Swan Tan S. Kostenhandleiding: Methodologie van kostenonderzoek en referentieprijzen voor economische evaluaties in de gezondheidszorg. Zorginstituut Ned. 2016;1–120.
17. National Institute of Public Health and the Environment (RIVM). Cancer numbers and context: current situation [Internet]. volksgezondheidenzorg.info. 2017. p. 1.
18. Briggs A, Claxton K, Sculpher M. Decision modelling for Health Economic Evaluation. 3rd ed. New York: Oxford University Press; 2011. 57–58 p.
19. Raskob GE, van Es N, Verhamme P, Carrier M, Di Nisio M, Garcia D, et al. Edoxaban for the Treatment of Cancer-Associated Venous Thromboembolism. N Engl J Med. 2018;378(7):615–24.
20. The Netherlands Cancer Registry. Survival statistics - all tumours 1961-2015 [Internet].
21. Heisen M, Treur MJ, Heemstra HE, Giesen EBW, Postma MJ. Cost-effectiveness analysis of rivaroxaban for treatment and secondary prevention of venous thromboembolism in the Netherlands. J Med Econ. 2017 Aug 3;20(8):813–24.
22. Medicijnkosten.nl. The National Health Care Institute (ZIN) [Internet].
23. Summary of Product Characteristics Fragmin. 2015;
24. Dutch Statistics. Length and weight of Dutch population [Internet]. 2017.
25. de Klerk M, de Boer A, Plaisier I, Schyns P, Kooiker S. Informele hulp: wie doet er wat? - Rapport - SCP [Internet]. 2015-35. 2015.
26. Lloyd AJ, Dewilde S, Noble S, Reimer E, Lee AYY. What Impact Does Venous Thromboembolism and Bleeding Have on Cancer Patients' Quality of Life? Value Heal. 2018;21(4):449–55.
27. Roman A, Barbera JA, Castillo MJ, Muñoz R, Escribano P. Health-related quality of life in a national cohort of patients with pulmonary arterial hypertension or chronic thromboembolic pulmonary hypertension. Arch Bronconeumol. 2013;49(5):181–8.
28. Stevanović J, de Jong LA, Kappelhoff BS, Dvortsin EP, Voorhaar M, Postma MJ. Dabigatran for the Treatment and Secondary Prevention of Venous Thromboembolism; A Cost-Effectiveness Analysis for the Netherlands. PLoS One. 2016;11(10):e0163550.
29. The Netherlands Cancer Registry. Prevalence statistics - all tumours [Internet].
30. Walker AJ, Card TR, West J, Crooks C, Grainge MJ. Incidence of venous thromboembolism in patients with cancer - a cohort study using linked United Kingdom databases. Eur J Cancer. 2013 Apr;49(6):1404–13.
31. Vedham V, Divi RL, Starks VL, Verma M. Multiple Infections and Cancer: Implications in Epidemiology. Technol Cancer Res Treat. 2014;13(2):177–94.
32. Mahé I, Chidiac J, Helfer H, Noble S. Factors influencing adherence to clinical guidelines in the management of cancer-associated thrombosis. J Thromb Haemost. 2016;14(11):2107–13.
33. Committee for Medicinal Products for Human Use (CHMP). Summary of product characteristics - rivaroxaban 20 mg [Internet].
34. Khorana AA, Soff GA, Kakkar AK, Vadhan-Raj S, Riess H, Wun T, et al. Rivaroxaban for Thromboprophylaxis in High-Risk Ambulatory Patients with Cancer. N Engl J Med. 2019 Feb;380(8):720–8.
35. Carrier M, Abou-Nassar K, Mallick R, Tagalakis V, Shivakumar S, Schattner A, et al. Apixaban to Prevent Venous Thromboembolism in Patients with Cancer. N Engl J Med. 2019;380(8):711–9.
36. Agnelli G. Direct Oral Anticoagulants for Thromboprophylaxis in Ambulatory Patients with Cancer. N Engl J Med. 2019;380(8):781–3.

Author contributions

For peer review only

Lisa A. de Jong built the economic model, performed the analyses, and contributed to the design of the work, interpretation of the results, writing of the manuscript.

Annette W.G. van der Velden contributed to the interpretation of the results, writing of the manuscript, and critical revision for important intellectual content.

Marinus van Hulst contributed to the design of the work, interpretation of the results, writing of the manuscript, and critical revision for important intellectual content.

Maarten J. Postma contributed to the design of the work, interpretation of the results, writing of the manuscript, and critical revision for important intellectual content.

All authors approved of the version to be published.

Checklist for the appropriate reporting statement: This manuscript was written in accordance with the CHEERS checklist for reporting economic evaluations of health interventions.

Data sharing statement: All relevant data are included in the manuscript. The analyses were conducted based on publicly available information which is presented and referenced in the manuscript.

Word count: 4,508 (excluding table and figure descriptions and references)

Patient consent and ethical approval: The analyses were conducted based on publicly available information which is presented and referenced in the article and Supporting Information files, and did therefore not require any patient consent forms or approval from an ethical review board.

Patient and public involvement statement: It was not appropriate to involve patients or the public in the design, or conduct, or reporting, or dissemination plans of our research, because this health economic analysis was based on publicly available data and solely concentrated on the analysis of the economics consequence of treating cancer patients with rivaroxaban instead of the current standard of care.

For peer review only

For peer review only - http://bmjopen.bmj.com/site/about/guidelines.xhtml

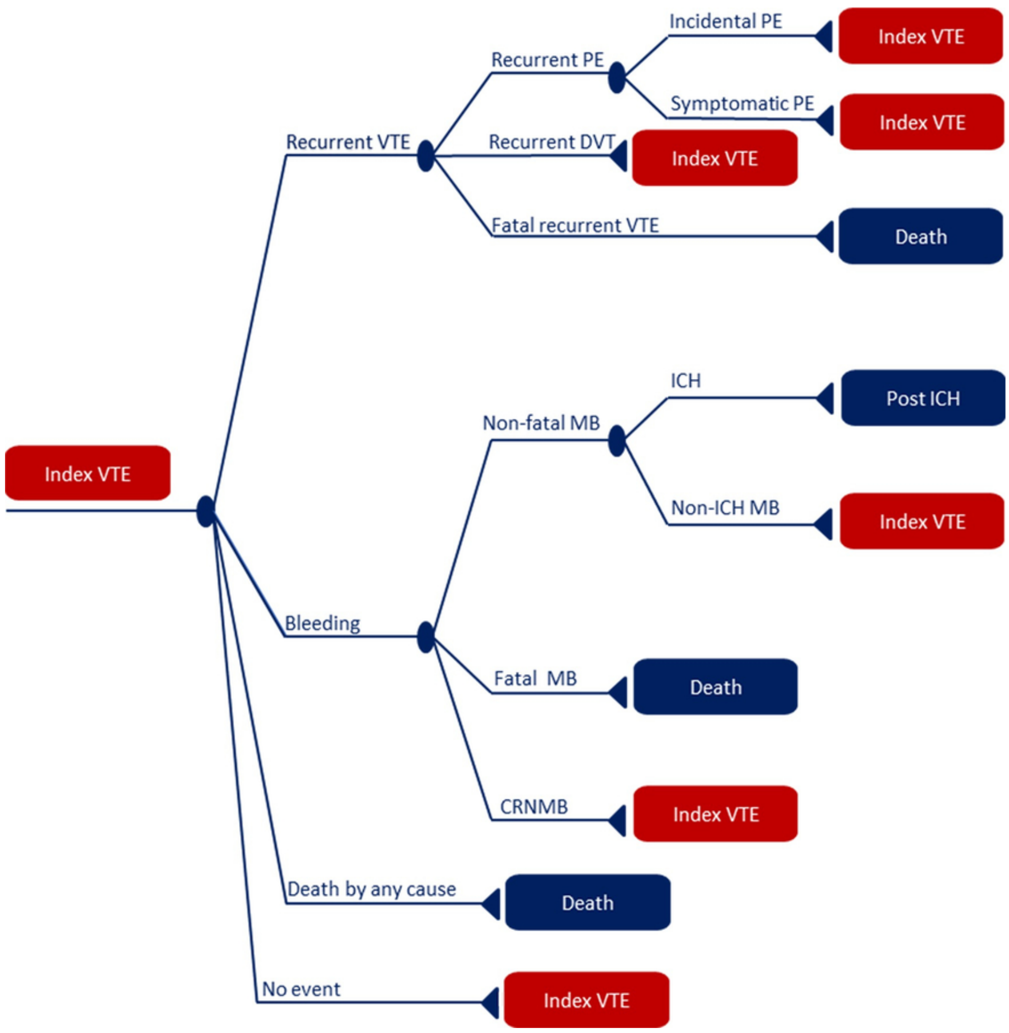

Figure 1. Model outline. All patients enter the model in the 'Index VTE' state and move to other states upon the occurrence of one of the following events: recurrent incidental PE, recurrent symptomatic PE, fatal recurrent VTE, recurrent DVT, ICH, non-ICH MB, fatal MB, CRNMB, or death by any cause. The triangles represent the health state a patient will enter after an event. The blue squares are permanent states, in which a patient will remain until death while not being at risk for other events. The red squares represent a transient state: the patient will re-enter the model in the 'Index VTE' state.
Abbreviations: CRNMB, clinically relevant non-major bleeding; DVT, deep vein thrombosis; ICH, intracranial haemorrhage; MB, major bleeding; PE, pulmonary embolism; VTE, venous thromboembolism

74x75mm (300 x 300 DPI)

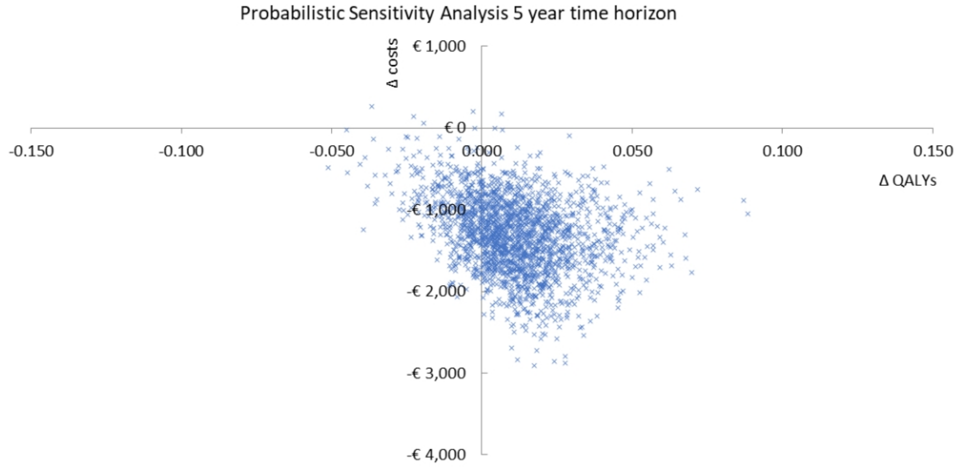

Probabilistic sensitivity analysis of the base case with five year time horizon (base case analysis).
Abbreviation: QALY, quality adjusted life-year

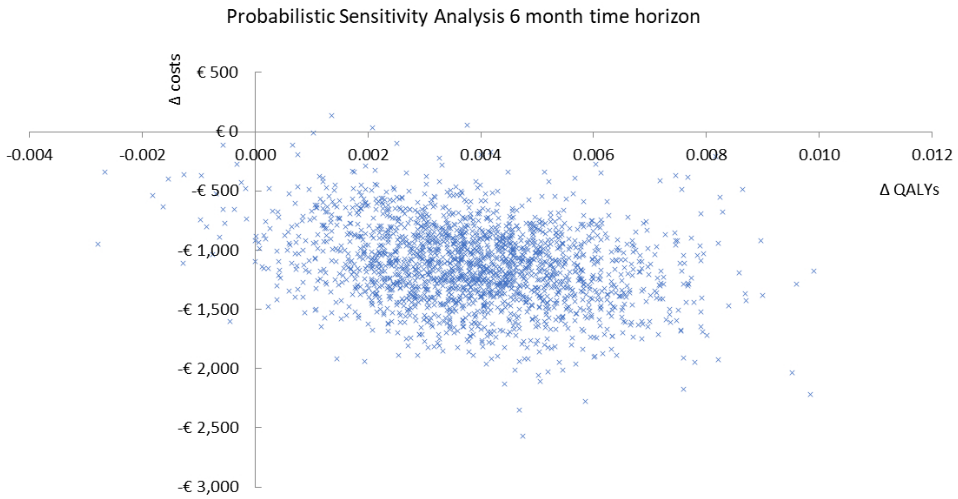

Probabilistic sensitivity analysis with six month time horizon (scenario 4). Abbreviation: QALY, quality adjusted life-year

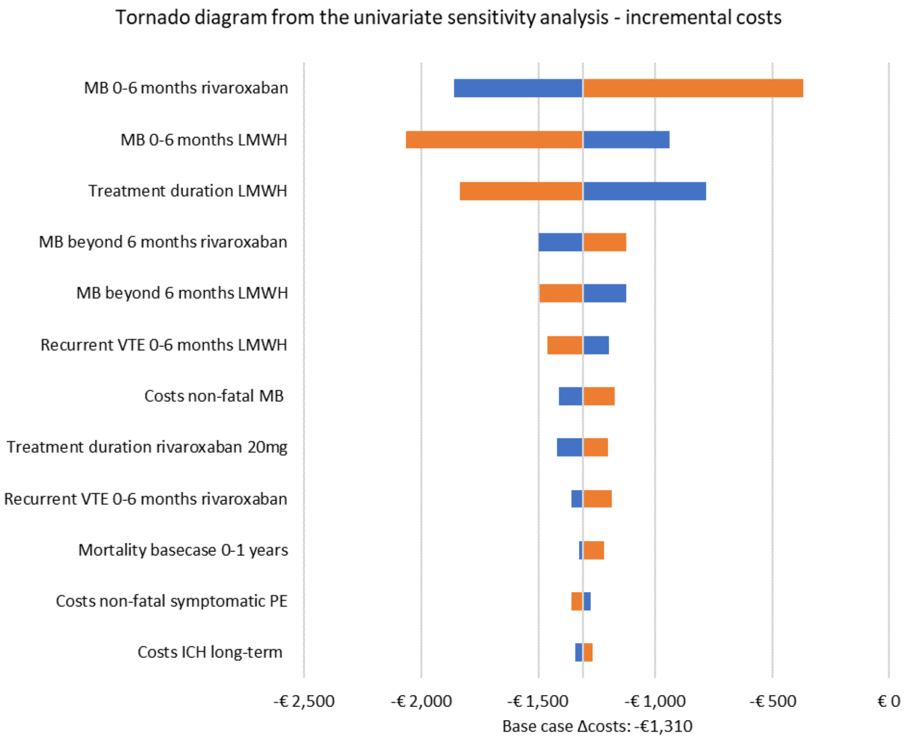

Tornado diagram from the univariate sensitivity analysis for the base case analysis showing the impact of parameters on the incremental costs. Abbreviations: ICH, intracranial haemorrhage; LMWH, low molecular weight heparin; MB, major bleeding; PE, pulmonary embolism; VTE, venous thromboembolism

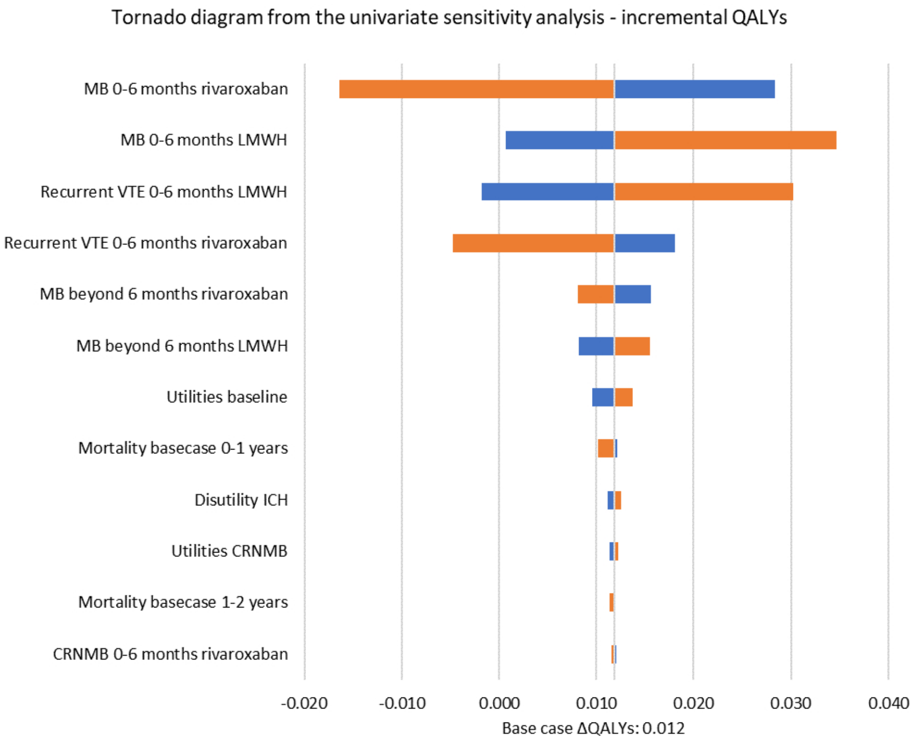

Tornado diagram from the univariate sensitivity analysis for the base case analysis showing the impact of parameters on the incremental QALYs. Abbreviations: CRNMB, clinically relevant non-major bleeding; ICH, intracranial haemorrhage; LMWH, low molecular weight heparin; MB, major bleeding; VTE, venous thromboembolism

Supplementary data file – Table S1

Manuscript title: Cost-effectiveness analysis and budget impact of rivaroxaban in cancer patients at risk of recurrent venous thromboembolism

Table S1. Transition probabilities used in the cost-effectiveness model

	Rivaroxaban (95% CI)	LMWH (95% CI)	Distribution	Reference
**Recurrent VTE**				
0–6 months	0.040 (0.020 – 0.090)	0.110 (0.070 – 0.160)	Beta	[1]
6–12 months	0.040 (0.031 – 0.050)		Beta	[2]
1–2 years	0.034 (0.027 – 0.042)		Beta	[2]
2–3 years	0.021 (0.014 – 0.029)		Beta	[2]
3–4 years	0.016 (0.009 – 0.026)		Beta	[2]
4–5 months	0.013 (0.006 – 0.024)		Beta	[2]
**Type of recurrent VTE**				
Symptomatic PE	17.4% (α = 4, β = 19)		Dirichlet	[1]
Incidental PE	30.4% (α = 7, β = 16)		Dirichlet	[1]
DVT	43.5% (α = 10, β = 13)		Dirichlet	[1]
Fatal PE	8.7% (α = 2, β = 21)		Dirichlet	[1]
**MB**				
0–6 months	0.060 (0.030 – 0.110)	0.040 (0.020 – 0.080)	Beta	[1]
Beyond 6 months treatment	0.008 (0.006 – 0.010)		Beta	[3]
**Type of MB**				
ICH	10% (α = 5, β = 45)		Dirichlet	[3]
Non-ICH MB	86% (α = 43, β = 7)		Dirichlet	[3]
Fatal MB	4% (α = 2, β = 48)		Dirichlet	[3]
**CRNMB**				
0–6 months	0.130 (0.090 – 0.190)	0.040 (0.020 – 0.090)	Beta	[1]
Beyond 6 months treatment	0.008 (0.006 – 0.010)		Beta	[3]
**PTS**				
0–6 months	0.015 (0.011 – 0.019)		Beta	[4]
6–12 months	0.012 (0.009 – 0.015)		Beta	[4]
12–18 months	0.008 (0.006 – 0.010)		Beta	[4]
18–24 months	0.025 (0.023 – 0.019)		Beta	[4]
24–30 months	0.011 (0.008 – 0.014)		Beta	[4]
30–36 months	0.006 (0.005 – 0.008)		Beta	[4]
3–4 years	0.001 (0.0008 – 0.0013)		Beta	[4]
4–5 years	0.001 (0.0008 – 0.0013)		Beta	[4]
**CTEPH (annual risk)**	0.0057 (0.0002 – 0.012)		Beta	[5]
**Mortality (annual risk)**				
0–1 years	0.230 (0.200 – 0.390)		Beta	[6]
1–2 years	0.104 (0.088 – 0.180)		Beta	[6]
2–3 years	0.058 (0.055 – 0.120)		Beta	[6]
3–4 years	0.046 (0.043 – 0.068)		Beta	[6]
4–5 years	0.032 (0.030 – 0.073)		Beta	[6]
**Relative risk of recurrent VTE, MB, and CRNMB for LMWH versus placebo, used in scenario 5**				
Recurrent VTE (any)	5.170		Fixed	[7]
MB	0.242		Fixed	[7]

For peer review only

CRNMB	1.000		Fixed	[7]
**Drug-specific distribution of the type of VTE, used in scenario 6**				
Symptomatic PE	28.6% (α = 2, β = 5)	12.5% (α = 2, β = 14)	Dirichlet	[1]
Incidental PE	14.3% (α = 1, β = 6)	37.5% (α = 6, β = 10)	Dirichlet	[1]
DVT	42.9% (α = 3, β = 4)	43.8% (α = 7, β = 9)	Dirichlet	[1]
Fatal PE	14.3% (α = 1, β = 6)	6.3% (α = 1, β = 15)	Dirichlet	[1]
**Drug-specific distribution of the type of MB, used in scenario 6**				
ICH	6.1% (α = 2, β = 31)	17.6% (α = 3, β = 14)	Dirichlet	[3]
Non-ICH MB	93.9% (α = 31, β = 2)	70.6% (α = 12, β = 5)	Dirichlet	[3]
Fatal MB	0% (α = 0, β = 33)	11.8% (α = 2, β = 15)	Dirichlet	[3]

Abbreviations: CI, confidence interval; CRNMB, clinically relevant non-major bleeding; CTEPH, chronic thromboembolic pulmonary hypertension; DVT, deep vein thrombosis; ICH, intracranial haemorrhage; LMWH, low-molecular weight heparin; MB, major bleeding; PE, pulmonary embolism; PTS, post-thrombotic syndrome; SE, standard error; VTE, venous thromboembolism

For peer review only

References

1. Young AM, Marshall A, Thirlwall J, Chapman O, Lokare A, Hill C, et al. Comparison of an Oral Factor Xa Inhibitor With Low Molecular Weight Heparin in Patients With Cancer With Venous Thromboembolism: Results of a Randomized Trial (SELECT-D). J Clin Oncol. 2018;36(20):2017–23.
2. Cohen AT, Katholing A, Rietbrock S, Bamber L, Martinez C. Epidemiology of first and recurrent venous thromboembolism in patients with active cancer. Thromb Haemost. 2017;117(01):57–65.
3. Raskob GE, van Es N, Verhamme P, Carrier M, Di Nisio M, Garcia D, et al. Edoxaban for the Treatment of Cancer-Associated Venous Thromboembolism. N Engl J Med. 2018;378(7):615–24.
4. Prandoni P, Villalta S, Bagatella P, Rossi L, Marchiori A, Piccioli A, et al. The clinical course of deep-vein thrombosis. Prospective long-term follow-up of 528 symptomatic patients. Haematologica. 1997;82(4):423–8.
5. Klok FA, van Kralingen KW, van Dijk APJ, Heyning FH, Vliegen HW, Huisman M V. Prospective cardiopulmonary screening program to detect chronic thromboembolic pulmonary hypertension in patients after acute pulmonary embolism. Haematologica. 2010;95(6):970–5.
6. The Netherlands Cancer Registry. Survival statistics - all tumours 1961-2015 [Internet].
7. Napolitano M, Saccullo G, Malato A, Sprini D, Ageno W, Imberti D, et al. Optimal duration of low molecular weight heparin for the treatment of cancer-related deep vein thrombosis: the Cancer-DACUS Study. J Clin Oncol. 2014;32(32):3607–12.

Supplementary data file – Table S2

Manuscript title: Cost-effectiveness analysis and budget impact of rivaroxaban in cancer patients at risk of recurrent venous thromboembolism

Table S2. Costs included in the cost-effectiveness model (Euros, 2019)

	Value (95% CI)	Distribution	Reference
**Event costs**			
Recurrent VTE			
Symptomatic PE	€4,717 (€2,364 – €7,868)	Gamma	[1]
Incidental PE	€0	Fixed	Assumption
DVT	€663 (€464 – €862)	Gamma	[1]
Fatal recurrent VTE [a]	€4,717 (€2,364 – €7,868)	Gamma	[1]
ICH acute care costs	€22,769 (€11,644 – €31,175)	Gamma	[2]
ICH long-term costs (monthly)	€637 (€319 – €1,063)	Gamma	[1]
Non-ICH MB	€10,685 (€5,356 – €17,824)	Gamma	[1]
Fatal MB	€10,685 (€5,356 – €17,824)	Gamma	[1]
CRNMB	€274 (€137 – €457)	Gamma	[1]
PTS	€1,431 (€717 – €2,387)	Gamma	[1]
CTEPH acute care costs	€7,843 (€3,931 – €16,433)	Gamma	[1]
CTEPH long-term costs (monthly)	€89 (€45 – €149)	Gamma	[1]
**Treatment costs**			
Drug cost (daily)			
LMWH [b]	€9.93	Fixed	[3]
Rivaroxaban 15 mg	€4.58	Fixed	[3]
Rivaroxaban 20 mg	€2.29	Fixed	[3]
Treatment duration (days)			
LMWH	183 (137 – 228)	Gamma	[4]
Rivaroxaban 15 mg	21 (16 – 26)	Gamma	[4]
Rivaroxaban 20 mg	162 (121 – 202)	Gamma	[4]
Renal monitoring [c]	€1.64 (€1.23 – €2.05)	Gamma	[5]
**Indirect costs**			
Travel costs			
Cost per km	€0.20 (€0.15 – €0.25)	Gamma	[6]
Distance to hospital (km)	7	Fixed	[6]
Distance to GP (km)	1.1	Fixed	[6]
Informal care costs			
PE	€1,515 (€1,136 – €1,894)	Gamma	[7,8]
DVT	€233 (€175 – €291)	Gamma	[7,8]
ICH (acute informal care costs)	€1,515 (€1,136 – €1,894)	Gamma	[7,8]
ICH (long-term informal care costs, monthly)	€626 (€470 – €783)	Gamma	[9]
Non-ICH MB	€758 (€568 – €947)	Gamma	[7,8]
CRNMB	€117 (€87 – €146)	Gamma	[7,8]

Abbreviations: CI, confidence interval; CRNMB, clinically relevant non-major bleeding; CTEPH, chronic thromboembolic pulmonary hypertension; DVT, deep vein thrombosis; GP, general practitioner; ICH, intracranial haemorrhage; LMWH, low-molecular weight heparin; MB, major bleeding; PE, pulmonary embolism; PTS, post-thrombotic syndrome; VTE, venous thromboembolism

[a] *Assumed to be equal to the costs of non-fatal PE*

[b] *Based on an average weight between 69 and 82 kg.*

[c] *Based on DRG code 070419 and only taken into account for rivaroxaban treated patients*

For peer review only

References

1. Heisen M, Treur MJ, Heemstra HE, Giesen EBW, Postma MJ. Cost-effectiveness analysis of rivaroxaban for treatment and secondary prevention of venous thromboembolism in the Netherlands. J Med Econ. 2017 Aug 3;20(8):813–24.
2. Baeten S a, van Exel NJ a, Dirks M, Koopmanschap M a, Dippel DW, Niessen LW. Lifetime health effects and medical costs of integrated stroke services - a non-randomized controlled cluster-trial based life table approach. Cost Eff Resour Alloc. 2010;8(1):21.
3. Medicijnkosten.nl. The National Health Care Institute (ZIN) [Internet].
4. Young AM, Marshall A, Thirlwall J, Chapman O, Lokare A, Hill C, et al. Comparison of an Oral Factor Xa Inhibitor With Low Molecular Weight Heparin in Patients With Cancer With Venous Thromboembolism: Results of a Randomized Trial (SELECT-D). J Clin Oncol. 2018;36(20):2017–23.
5. Dutch Health Authorities. NZa zorgproductapplicatie. Declaration code: 070419. [Internet]. 2018.
6. Roijen LH, Linden N van der, Bouwmans C, Kanters T, Tan SS. Dutch manual for costing studies in health care. Diemen; 2015.
7. Hakkaart-van Roijen L, van der Linden N, Bouwmans C, Kanters T, Swan Tan S. Kostenhandleiding: Methodologie van kostenonderzoek en referentieprijzen voor economische evaluaties in de gezondheidszorg. Zorginstituut Ned. 2016;1–120.
8. de Klerk M, de Boer A, Plaisier I, Schyns P, Kooiker S. Informele hulp: wie doet er wat? - Rapport - SCP [Internet]. 2015-35. 2015.
9. van den Berg B, Brouwer W, van Exel J, Koopmanschap M, van den Bos GAM, Rutten F. Economic valuation of informal care: lessons from the application of the opportunity costs and proxy good methods. Soc Sci Med. 2006;62(4):835–45.

For peer review only

Supplementary data file – Table S3

Manuscript title: Cost-effectiveness analysis and budget impact of rivaroxaban in cancer patients at risk of recurrent venous thromboembolism

Table S3. Utility values included in the cost-effectiveness model

	Value (95% CI)	Distribution	Reference
**Utilities**			
Index VTE			
0–1 month	0.565 (0.501 – 0.620)	Beta	[1]
1–2 months	0.655 (0.585 – 0.713)	Beta	[1]
2–3 months	0.674 (0.606 – 0.729)	Beta	[1]
3–4 months	0.698 (0.635 – 0.750)	Beta	[1]
4–5 months	0.707 (0.645 – 0.758)	Beta	[1]
5–6 months	0.709 (0.647 – 0.760)	Beta	[1]
Baseline utility 6 months after index VTE	0.715 (0.646 – 0.770)	Beta	[1]
Recurrent VTE			
DVT	0.605 (0.514 – 0.678)	Beta	[1]
Non-fatal symptomatic PE	0.621 (0.477 – 0.725)	Beta	[1]
Non-fatal incidental PE	0.664 (0.615 – 0.707)	Beta	[1]
Fatal PE	0.456 (0.268 – 0.595)	Beta	[1]
Non-ICH MB	0.593 (0.461 – 0.693)	Beta	[1]
CRNMB	0.622 (0.568 – 0.669)	Beta	[1]
**Utility decrements**			
Recurrent VTE within first six months after index VTE			
DVT	0.040 (0.000 – 0.158)	Beta	[1]
Symptomatic PE	0.024 (0.000 – 0.195)	Beta	[1]
Incidental PE	0.189 (0.021 – 0.404)	Beta	[1]
ICH	0.380 (0.285 – 0.475)	Beta	[2]
Severe PTS (<6 months after diagnosis)	0.186 (0.090 – 0.280)	Beta	[1]
Severe PTS (>6 months after diagnosis)	0.070 (0.053 – 0.088)	Beta	[2]
CTEPH			
0-1 year	0.194 (0.071 – 0.303)	Beta	[3]
1–4 years	0.109 (0.000 – 0.244)	Beta	[3]
4–7 years	0.079 (0.000 – 0.277)	Beta	[3]
>7 years	0.065 (0.000 – 0.164)	Beta	[3]

Abbreviations: CI, confidence interval; CRNMB, clinically relevant non-major bleeding; CTEPH, chronic thromboembolic pulmonary hypertension; DVT, deep vein thrombosis; ICH, intracranial haemorrhage; MB, major bleeding; PE, pulmonary embolism; PTS, post-thrombotic syndrome; VTE, venous thromboembolism

References

1. Lloyd AJ, Dewilde S, Noble S, Reimer E, Lee AYY. What Impact Does Venous Thromboembolism and Bleeding Have on Cancer Patients' Quality of Life? Value Heal. 2018;21(4):449–55.
2. Stevanović J, de Jong LA, Kappelhoff BS, Dvortsin EP, Voorhaar M, Postma MJ. Dabigatran for the Treatment and Secondary Prevention of Venous Thromboembolism; A Cost-Effectiveness Analysis for the Netherlands. PLoS One. 2016;11(10):e0163550.
3. Roman A, Barbera JA, Castillo MJ, Muñoz R, Escribano P. Health-related quality of life in a national cohort of patients with pulmonary arterial hypertension or chronic thromboembolic

For peer review only

pulmonary hypertension. Arch Bronconeumol. 2013;49(5):181–8.

For peer review only

CHEERS checklist—Items to include when reporting economic evaluations of health interventions

Section/item	Item No	Recommendation	Reported on page No/ line No
**Title and abstract**			
Title	1	Identify the study as an economic evaluation or use more specific terms such as "cost-effectiveness analysis", and describe the interventions compared.	Page 1, line 9-10
Abstract	2	Provide a structured summary of objectives, perspective, setting, methods (including study design and inputs), results (including base case and uncertainty analyses), and conclusions.	Page 1, line 34-64
**Introduction**			
Background and objectives	3	Provide an explicit statement of the broader context for the study.	Page 4, line 91-120
		Present the study question and its relevance for health policy or practice decisions.	Page 4, line 121-126
**Methods**			
Target population and subgroups	4	Describe characteristics of the base case population and subgroups analysed, including why they were chosen.	Page 5, line 149-151<br>Page 5, line 179
Setting and location	5	State relevant aspects of the system(s) in which the decision(s) need(s) to be made.	Page 4, line 130-140
Study perspective	6	Describe the perspective of the study and relate this to the costs being evaluated.	Page 5, line 135-137
Comparators	7	Describe the interventions or strategies being compared and state why they were chosen.	Page 4, line 130-132
Time horizon	8	State the time horizon(s) over which costs and consequences are being evaluated and say why appropriate.	Page 5, line 152-154
Discount rate	9	Report the choice of discount rate(s) used for costs and outcomes and say why appropriate.	Page 8, line 241<br>Page 9, line 264
Choice of health outcomes	10	Describe what outcomes were used as the measure(s) of benefit in the evaluation and their relevance for the type of analysis performed.	Page 4, line 133-140
Measurement of effectiveness	11a	*Single study-based estimates:* Describe fully the design features of the single effectiveness study and why the single study was a sufficient source of clinical effectiveness data.	
	11b	*Synthesis-based estimates*: Describe fully the methods used for identification of included studies and synthesis of clinical effectiveness data.	Page 6, line 184-210
Measurement and valuation of preference based outcomes	12	If applicable, describe the population and methods used to elicit preferences for outcomes.	
Estimating resources and costs	13a	*Single study-based economic evaluation:* Describe approaches used to estimate resource use associated with the alternative interventions. Describe primary or secondary research methods for valuing each resource item in terms of its unit cost. Describe any adjustments made to approximate to opportunity costs.	
	13b	*Model-based economic evaluation:* Describe approaches and data sources used to estimate	Page 8, line 213-253

For peer review only

Section/item	Item No	Recommendation	Reported on page No/ line No
		resource use associated with model health states. Describe primary or secondary research methods for valuing each resource item in terms of its unit cost. Describe any adjustments made to approximate to opportunity costs.	
Currency, price date, and conversion	14	Report the dates of the estimated resource quantities and unit costs. Describe methods for adjusting estimated unit costs to the year of reported costs if necessary. Describe methods for converting costs into a common currency base and the exchange rate.	Page 8, line 246
Choice of model	15	Describe and give reasons for the specific type of decision-analytical model used. Providing a figure to show model structure is strongly recommended.	Page 5, line 143-176
Assumptions	16	Describe all structural or other assumptions underpinning the decision-analytical model.	Page 5, line 162-166<br>Page 8, line 230-240
Analytical methods	17	Describe all analytical methods supporting the evaluation. This could include methods for dealing with skewed, missing, or censored data; extrapolation methods; methods for pooling data; approaches to validate or make adjustments (such as half cycle corrections) to a model; and methods for handling population heterogeneity and uncertainty.	Page 7, line 190-201<br>Page 10, line 284-301
**Results**			
Study parameters	18	Report the values, ranges, references, and, if used, probability distributions for all parameters. Report reasons or sources for distributions used to represent uncertainty where appropriate. Providing a table to show the input values is strongly recommended.	Page 6, line 201-210<br>Page 8, line 241-253<br>Page 9, line 264-271<br>Page 10, line 276-278
Incremental costs and outcomes	19	For each intervention, report mean values for the main categories of estimated costs and outcomes of interest, as well as mean differences between the comparator groups. If applicable, report incremental cost-effectiveness ratios.	Page 11, line 320-323<br>Page 14, line 393-398
Characterising uncertainty	20a	*Single study-based economic evaluation:* Describe the effects of sampling uncertainty for the estimated incremental cost and incremental effectiveness parameters, together with the impact of methodological assumptions (such as discount rate, study perspective).	
	20b	*Model-based economic evaluation:* Describe the effects on the results of uncertainty for all input parameters, and uncertainty related to the structure of the model and assumptions.	Page 13, line 354-389
Characterising heterogeneity	21	If applicable, report differences in costs, outcomes, or cost-effectiveness that can be explained by variations between subgroups of patients with different baseline characteristics or other observed variability in effects that are not reducible by more information.	Page 12, line 311-333
**Discussion**			
Study findings, limitations, generalisability, and current knowledge	22	Summarise key study findings and describe how they support the conclusions reached. Discuss limitations and the generalisability of the findings and how the findings fit with current knowledge.	Page 11, line 323-351
**Other**			

For peer review only

Section/item	Item No	Recommendation	Reported on page No/ line No
Source of funding	23	Describe how the study was funded and the role of the funder in the identification, design, conduct, and reporting of the analysis. Describe other non-monetary sources of support.	Page 3, line 79
Conflicts of interest	24	Describe any potential for conflict of interest of study contributors in accordance with journal policy. In the absence of a journal policy, we recommend authors comply with International Committee of Medical Journal Editors recommendations.	Page 3, line 81-84

For consistency, the CHEERS statement checklist format is based on the format of the CONSORT statement checklist

For peer review only

Cost-effectiveness analysis and budget impact of rivaroxaban compared with dalteparin in cancer patients at risk of recurrent venous thromboembolism

Journal:	*BMJ Open*
Manuscript ID	bmjopen-2020-039057.R1
Article Type:	Original research
Date Submitted by the Author:	04-Aug-2020
Complete List of Authors:	de Jong, Lisa; University of Groningen, Unit of Pharmacotherapy, -Epidemiology and -Economics<br>van der Velden, Annette; Martini Hospital, Department of Internal Medicine<br>Hulst, Marinus; Martini Hospital, Department of Clinical Pharmacy and Toxicology; University Medical Centre Groningen, Department of Health Sciences<br>Postma, Maarten; University Medical Centre Groningen, Department of Health Sciences; University of Groningen, Department of Economics, Econometrics & Finance
**Primary Subject Heading**:	Health economics
Secondary Subject Heading:	Cardiovascular medicine, Health economics
Keywords:	Anticoagulation < HAEMATOLOGY, ONCOLOGY, Thromboembolism < CARDIOLOGY, HEALTH ECONOMICS

SCHOLARONE™ Manuscripts

I, the Submitting Author has the right to grant and does grant on behalf of all authors of the Work (as defined in the below author licence), an exclusive licence and/or a non-exclusive licence for contributions from authors who are: i) UK Crown employees; ii) where BMJ has agreed a CC-BY licence shall apply, and/or iii) in accordance with the terms applicable for US Federal Government officers or employees acting as part of their official duties; on a worldwide, perpetual, irrevocable, royalty-free basis to BMJ Publishing Group Ltd ("BMJ") its licensees and where the relevant Journal is co-owned by BMJ to the co-owners of the Journal, to publish the Work in this journal and any other BMJ products and to exploit all rights, as set out in our licence.

The Submitting Author accepts and understands that any supply made under these terms is made by BMJ to the Submitting Author unless you are acting as an employee on behalf of your employer or a postgraduate student of an affiliated institution which is paying any applicable article publishing charge ("APC") for Open Access articles. Where the Submitting Author wishes to make the Work available on an Open Access basis (and intends to pay the relevant APC), the terms of reuse of such Open Access shall be governed by a Creative Commons licence – details of these licences and which Creative Commons licence will apply to this Work are set out in our licence referred to above.

Other than as permitted in any relevant BMJ Author's Self Archiving Policies, I confirm this Work has not been accepted for publication elsewhere, is not being considered for publication elsewhere and does not duplicate material already published. I confirm all authors consent to publication of this Work and authorise the granting of this licence.

For peer review only

Cost-effectiveness analysis and budget impact of rivaroxaban compared with dalteparin in cancer patients at risk of recurrent venous thromboembolism

Short title: Economic evaluation of rivaroxaban in cancer patients

Authors:

Lisa A. de Jong[1], Annette W.G. van der Velden[2], Marinus van Hulst[3,4]*, Maarten J. Postma[4,5]*

Affiliations:

[1] Unit of PharmacoTherapy, -Epidemiology & -Economics, University of Groningen, Groningen Research Institute of Pharmacy (GRIP), Antonius Deusinglaan 1, Groningen, the Netherlands

[2] Department of Internal Medicine, Martini Hospital, Van Swietenplein 1, Groningen, The Netherlands

[3] Department of Clinical Pharmacy and Toxicology, Martini Hospital, Van Swietenplein 1, Groningen, The Netherlands

[4] Department of Health Sciences, University of Groningen, University Medical Center Groningen, Hanzeplein 1, Groningen, The Netherlands

[5] Department of Economics, Econometrics & Finance, University of Groningen, Faculty of Economics & Business, Nettelbosje 2, Groningen, The Netherlands

*Shared last author

Corresponding author:

Lisa A de Jong

E-mail: l.a.de.jong@rug.nl

Phone: +31630048956

ORCID ID: 0000-0001-8814-0670

Author contributions:

LA de Jong contributed to the design, interpretation of the data, modelling, drafting the manuscript and revisions. M van Hulst, MJ Postma and AWG van der Velden contributed to the design, interpretation of the data, validation of the model and drafting the manuscript.

For peer review only - http://bmjopen.bmj.com/site/about/guidelines.xhtml

Abstract

Objectives: We aim to calculate the cost-effectiveness and budget impact of rivaroxaban compared with dalteparin in cancer patients at risk of recurrent venous thromboembolism (VTE).

Setting: The analyses were performed for the Dutch healthcare setting. We built a Markov model to calculate the cost-effectiveness from a societal perspective over a five-year time horizon.

Participants: A hypothetical cohort of 1,000 cancer patients with VTE entered the model with baseline characteristics based on the SELECT-D trial.

Intervention: Six months treatment with rivaroxaban (15 mg twice daily for first three weeks followed by 20 mg once daily) was compared with six months treatment with dalteparin (200 IU/kg daily during month one followed by 150 IU/kg daily).

Primary and secondary outcome measures: The primary outcome of the cost-effectiveness analysis was the incremental cost-effectiveness ratio (ICER). The robustness of the model was evaluated in probabilistic and univariate sensitivity analyses. A budget impact analysis was performed to calculate the total annual financial consequences for the society.

Results: In the base case and all scenarios, rivaroxaban were cost-saving while also slightly improving the patient's health, resulting in economically dominant ICERs. In the probabilistic sensitivity analysis, 77.8% and 98.7% of the simulations showed rivaroxaban to be cost-saving and more effective for a five year and six-month time horizon, respectively. Rivaroxaban can save up to €11,326,763 (confidence interval: €5,164,254–€17,363,231) in approximately 8,000 cancer patients with VTE per year compared with dalteparin based on a one-year time horizon.

Conclusions: Treatment with rivaroxaban is economically dominant over dalteparin in cancer patients at risk for recurrent VTE in the Netherlands. The use of rivaroxaban instead of a LMWH can save up to ten million euros per year, primarily driven by the difference in drug costs.

Strengths and limitations of this study

- This analysis includes both cost-effectiveness and budget impact analyses, presenting the economic impact on a patient as well as on a population level.
- Markov tunnel states were used to model the occurrence of time-dependent events.
- Various additional scenarios were used to analyse the effect of different assumptions and clinical situations.
- We assumed a six-month treatment duration for all patients, while in clinical practice the treatment duration may vary between patients.
- Due to lack of data, the productivity losses were not taken into account.

Funding statement: This work was supported by Bayer Pharma Netherlands. The sponsor was involved with the start of the project, but they were not involved in the identification of data, design, conduct, and reporting of the analysis. Award/grant number: not applicable.

Competing interests: LA De Jong, M van Hulst and AWG van der Velden declare that they have no competing interest with relation to subject. Postma MJ has received research grants from various pharmaceutical companies, including but not limiting to Bayer, Pfizer, Bristol-Myers Squibb, GSK, Roche and Novartis.

For peer review only - http://bmjopen.bmj.com/site/about/guidelines.xhtml

Introduction

Venous thromboembolism (VTE), comprising both pulmonary embolism (PE) and deep vein thrombosis (DVT), is a major challenge in patients with cancer [1]. In addition to the characteristics of the cancer itself, cancer therapy (chemotherapy and cancer surgery) has effects on the patient's coagulation system and therefore increases the risk of VTE and bleeding [2,3]. VTE in cancer patients can cause unnecessary hospitalizations, interruption or postponement of cancer treatment, and increased mortality, leading to decreased quality of life and increased costs.

VTE is treated with anticoagulation therapy, and this is continued as prophylaxis for recurrence over a longer period because of the high risk of recurrence during the first months after the initial VTE [4]. Vitamin K antagonists (VKAs) or direct oral anticoagulants (DOACs) are indicated for the treatment and prevention of VTE in the general population [5]. DOACs, are a relatively new class of anticoagulants. Apixaban, dabigatran, edoxaban and rivaroxaban are the four DOACs that are currently registered for the prevention of recurrent VTE in Europe. DOACs have a more beneficial efficacy/safety ratio, do not require routine measurements of the INR, and show fewer food-drug and drug-drug interactions compared with VKAs [6,7].

The guidelines recommend against the use of VKAs in cancer patients because of potential drug interactions, liver dysfunction, and malnutrition, all of which lead to fluctuations of the international normalized ratio (INR) and could result in negative patient outcomes [8–11]. Moreover, trials in cancer patients with VTE have shown that LMWH is more effective in the prevention of recurrent VTE compared with VKA, without increasing bleeding risk [12–14]. Therefore, the guidelines recommend at least 6 months of therapeutic treatment with a daily subcutaneous injection of low molecular weight heparin (LMWH, e.g., dalteparin) in cancer patients [8–11]. However, recently, DOACs rivaroxaban and edoxaban were also added as treatment options for the prevention of recurrent VTE in cancer patients. This recommendation was based on the results from the SELECT-D and HOKUSAI VTE Cancer trials [15,16].

The SELECT-D is a multicenter, randomized, clinical pilot trial in the UK; it is a head-to-head comparison of rivaroxaban and dalteparin in 406 patients with active cancer who had experienced a symptomatic PE, incidental PE, or symptomatic DVT [15]. Incidental PEs are non-symptomatic PEs that are incidentally found during tumour imaging. The trial researchers found that rivaroxaban reduces the recurrence of VTE (six month cumulative VTE recurrence rate: 4% versus 11%) at the cost of an increased risk of bleeding (six month cumulative major bleeding [MB] rate: 6% versus 4%; six month cumulative clinically relevant non-major bleeding [CRNMB] rate: 13% versus 4%) compared with dalteparin. These results were comparable to those of a large retrospective study by Streiff et al. [17].

Based on the results of these studies and the fact that DOACs can be orally administered (unlike the subcutaneously injected LMWHs), a greater utilisation of DOACs for VTE in cancer patients might be expected. Since the introduction of DOACs there has been an ongoing discussion about the economic impact of these drugs. By designing an economic model based on the SELECT-D trial, we aim to evaluate the cost-effectiveness and budget impact of rivaroxaban compared with dalteparin in cancer patients at risk of recurrent VTE in the Netherlands.

Methods

The economic model comparing rivaroxaban with dalteparin was designed based on the SELECT-D trial [15], since this study presented the most comprehensive results reflecting recurrent VTE and bleeding

For peer review only - http://bmjopen.bmj.com/site/about/guidelines.xhtml

complications per event type (symptomatic PE, incidental PE, and DVT) or severity (MB and CRNMB). The primary outcome of the cost-effectiveness analysis is the incremental cost-effectiveness ratio (ICER); this is calculated by dividing the incremental costs by the incremental health effects, expressed in quality adjusted life-years (QALYs). In accordance with Dutch costing guidelines for economic evaluations in healthcare, the ICER was calculated from a societal perspective, which incorporates direct as well as indirect costs both inside and outside the healthcare sector[18]. We performed sensitivity and scenario analyses to test the robustness of the model. Additionally, we conducted a budget impact analysis to reflect the annual financial consequences of the use of rivaroxaban in cancer patients at risk of recurrent VTE in the Netherlands. The analysis was carried out early 2019. The analyses were conducted based on publicly available information which is presented and referenced in the article and Supporting Information files, and did therefore not require any patient consent forms or approval from an ethical review board.

Model outline

We developed a decision-tree-based Markov model using Microsoft Excel 2016 to calculate the ICER. Figure 1 shows a schematic representation of the model, with the disease course being represented by separate health states. A hypothetical cohort of 1,000 cancer patients with VTE entered the model with incidental PE, symptomatic PE, or DVT, represented by the 'index VTE' health state. According to the guidelines, patients with incidental PE should be treated identically to those with symptomatic PE [8,10]. Patient characteristics were based on the SELECT-D trial protocol (Table 1) [15]. The SELECT-D population is representative for the Dutch cancer population, based on age, tumour type, and gender distribution [19]. Patients move through various health states in the model during the follow-up time of five years. Five years was used because overall survival was assumed to be low after five years since the majority (58%) of the SELECT-D trial population had metastatic cancer [15]. We included the following health states in our model (see legend of Figure 1 for abbreviations): 'recurrent incidental PE', 'recurrent symptomatic PE', 'fatal recurrent VTE', 'recurrent DVT', 'ICH', 'non-ICH MB', 'fatal MB', 'CRNMB', 'death by any cause', and 'no event'. Patients were assumed to remain in these states for one cycle, after which they moved back to the 'index VTE' state or the chronic, debilitating 'post-ICH' state, in which they remained until death without being at risk for any further complications. The cycle length was one month. Markov tunnel states (one-month post-VTE, two months post-VTE, …, 60 months post VTE) were used to implement time-dependency. These temporary states can only be visited once, which allows time-dependent future transitions, costs, and health-related quality of life dependent on how long the patient has gone without a recurrent VTE event [20]. The chronic complications post-thrombotic syndrome (PTS) and chronic thromboembolic pulmonary hypertension (CTEPH) were modelled in the background. This means that PTS or CTEPH could occur at any time in the model, regardless of the health state the patient is in. Costs and health effects of these events were taken into account. However, only the severe cases of PTS were modelled, since the costs of minor PTS are considered negligible. For these chronic complications we also used tunnel states since the risks of PTS and CTEPH were also time-dependent.

Figure 1. Model outline. All patients enter the model in the 'Index VTE' state and move to other states upon the occurrence of one of the following events: recurrent incidental PE, recurrent symptomatic PE, fatal recurrent VTE, recurrent DVT, ICH,

For peer review only - http://bmjopen.bmj.com/site/about/guidelines.xhtml

non-ICH MB, fatal MB, CRNMB, or death by any cause. The triangles represent the health state a patient will enter after an event. The blue squares are permanent states, in which a patient will remain until death while not being at risk for other events. The red squares represent a transient state: the patient will re-enter the model in the 'Index VTE' state.

Abbreviations: CRNMB, clinically relevant non-major bleeding; DVT, deep vein thrombosis; ICH, intracranial haemorrhage; MB, major bleeding; PE, pulmonary embolism; VTE, venous thromboembolism

Table 1. Patient characteristics of the hypothetical cohort of 1,000 cancer patients at risk of recurrent VTE.

Unit	Value	Reference
Age (years)	67	[15]
Proportion male	53%	[15]
BMI (kg/m^2)	25.6	[15]
Type of cancer		
Early or locally advanced cancer	39%	[15]
Metastatic cancer	58%	[15]
Haematologic malignancy	2%	[15]
Distribution of PE and DVT		
% index VTE that is symptomatic PE	20%	[15]
% index VTE that is incidental PE	53%	[15]
% index VTE that is DVT	27%	[15]

Abbreviations: BMI, body mass index; DVT, deep vein thrombosis; PE, pulmonary embolism; VTE, venous thromboembolism

Transition probabilities

Transition probabilities were used to calculate the number of patients in each health state per one-month cycle. Table S1 summarizes all event rates presented in six-month risks. The event rates were translated into monthly transition probabilities with the following formula:

$$P = 1 - \exp\{-rt\}$$

Where P is the transition probability, r is the event rate, and t is the cycle length (one month) [20].

Event rates of recurrent VTE, MB, and CRNMB in the first six months of treatment were based on the SELECT-D trial [15]. If patients did not experience a recurrent event during this period, anticoagulation treatment was discontinued. Recurrent VTE rates after treatment discontinuation were based on a retrospective study in active-cancer patients experiencing a VTE [4]. Upon the occurrence of a non-fatal recurrent VTE, patients were assigned to another six months treatment, with corresponding event rates. Bleeding risks after treatment discontinuation were based on the outcomes of the cancer population of the HOKUSAI VTE Cancer trial (which followed patients after edoxaban discontinuation for an additional six months) because these data is not reported for the SELECT-D trial [16]. The HOKUSAI VTE Cancer trial was also used to determine the distribution of ICH, non-ICH, and fatal bleeding. The distributions among the different types of VTE (incidental PE, symptomatic PE, DVT, and fatal PE) and MB (ICH, non-ICH, fatal MB) were calculated based on the total number of events in both arms (rivaroxaban and dalteparin) together and assumed it to be treatment-independent, since the total number of events in the trials was low. The distributions of the types of VTE event were based on the number of recurrent VTE events observed in the SELECT-D trial in the lower extremities and pulmonary embolisms— other locations of VTE events (brachial, subclavian, jugular, renal plus inferior vena cava, or the extrahepatic vein) were excluded [15]. Mortality rates (death by any cause) were based on Dutch cancer mortality data from the Netherlands Cancer Registry [21]. In the sensitivity

For peer review only - http://bmjopen.bmj.com/site/about/guidelines.xhtml

analysis, all transition probabilities were varied over beta distributions. For percentages of the type of recurrent VTE and MB, a Dirichlet distribution was used in the sensitivity analysis. As recommended by the Dutch guidelines for economic evaluation of healthcare, the distributions were based on Briggs et al., who described the use of distributions around model input parameters (e.g., distributions limited to positive values (costs) or even confined between 0-1 (probabilities)) [18,20].

Costs

All cost parameters are standardised to 2019 Euros, and summarised in Table S2. Event-related healthcare costs were based on a previous Dutch cost-effectiveness study for rivaroxaban in the general VTE population [22]. Costs of fatal recurrent VTE were assumed to be similar to those of non-fatal symptomatic PE. We assumed no event-related healthcare costs for patients with incidental PE, because these embolisms were found incidentally and did therefore not require physician visits. However, since patients with incidental PE should be treated identically to those with symptomatic PE, we did take medication costs into account. Costs for ICH and CTEPH consisted of acute care costs during the first month after diagnosis, followed by long-term care costs until the patient moved to the 'death' state. Costs of a fatal MB were assumed to be equal to those of non-fatal non-ICH MB.

Drug costs were retrieved from the national medication costs database [23]. For rivaroxaban these costs were based on 15 mg twice daily for three weeks followed by 20 mg once daily. Drug costs of dalteparin were based on 200 IU/kg daily during month one followed by 150 IU/kg daily in months two to six [15,24]. Based on an average body mass index of 25.6 from the SELECT-D trial and an average height of 1.72 m for the Dutch population, we calculated that the average weight was between 69 and 82 kg, which corresponds with a dose of 15,000 IU daily during month one followed by 12,500 IU daily in months two to six [15,25]. Rivaroxaban users were assumed to require an annual check-up of their renal function [6]. We included one-time costs for an injection instruction by a home-caregiver. Administration costs were only accounted to patients with early or locally advanced cancer (39%), since patients with metastatic cancer or haematologic malignancies often already have home care or an informal caregiver who can administer the dalteparin injection. Similarly, informal care costs were only taken into account for this same subgroup.

Based on a previously published report on informal care in the Netherlands, we made a distinction between intensive (26 hours per week) and non-intensive (8 hours per week) informal care [26]. This was multiplied by the average duration and tariff for informal care, obtained from the Dutch cost manual [27]. To prevent double counting, we did not include informal care costs for the chronic complications. Travel costs were taken into account for renal monitoring visits and upon the occurrence of a DVT or CRNMB. Costs related to forgone leisure activity were not taken into account since there is no data available on the impact of a VTE or bleeding on leisure losses in cancer patients. Moreover, the starting age of the population in the model was 67 years (which is the Dutch retirement age) based on the average age of the SELECT-D trial and the fact that the majority (58%) of the patients in the SELECT-D trial had metastatic cancer may indicate a low employment rate.

Costs were discounted at an annual rate of 4% [18]. In the sensitivity analysis, the costs were varied with gamma distributions corresponding to the 95% confidence interval (CI) [18,20], as indicated in Table S2.

For peer review only - http://bmjopen.bmj.com/site/about/guidelines.xhtml

Utilities

Utility scores, used to calculate the QALYs, were derived from a sub-analysis from the CATCH study assessing the EQ-5D scores associated with VTE and recurrent VTE in cancer patients (Table S3) [28]. The CATCH study assessed the effectiveness of six months of treatment with tinzaparin versus warfarin for the treatment of acute venous thromboembolism in patients with active cancer. It was chosen because it aligns well with our population and events of interest. Utility decrements for CTEPH were based on a study assessing EQ-5D VAS scores in CTEPH patients up to 5 years after their initial diagnosis [29]. Utility decrements for ICH and long-term PTS (>6months after diagnosis) were obtained from a previous cost-effectiveness study [30]. QALYs related to fatal events, such as death to any cause, fatal PE and fatal MB, were assumed to be 0. QALYs were discounted at 1.5% per annum according to Dutch guidelines [18]. In the sensitivity analyses, utility scores were varied over their 95% CI with a beta distribution [18,20].

Sensitivity analysis

Sensitivity analyses were conducted to check the robustness of the model results to uncertainty and known variations in key input parameters. In the probabilistic sensitivity analysis, all input parameters were varied simultaneously over their 95% CI. If the 95% CI was unavailable and calculating the 95% CI based on the number of events was not possible, the 95% CI was calculated based on a 25% standard error. The ICER was calculated with 2,000 iterations and plotted in a cost-effectiveness plane. A univariate sensitivity analysis was conducted to show the influence of an individual parameter on the ICER. The 12 most influencing parameters were presented in a tornado diagram.

Scenario analysis

We conducted several scenario analyses to show the effect on the outcomes of different (clinical) situations (Table 2).

Table 2. Overview of the scenario analyses.

<table>
<tr><th>Scenario</th><th>Description</th><th>Details</th></tr>
<tr><td>Base case</td><td>5-year time horizon from societal perspective</td><td>-</td></tr>
<tr><td>1</td><td>6-month time horizon from societal perspective</td><td>The follow-up period of the SELECT-D trial was six months; therefore, outcomes beyond six months had to be extrapolated based on other publications.</td></tr>
<tr><td>2</td><td>Base case analysis from healthcare payer's perspective</td><td>In the Netherlands, guidelines advise to calculate the ICER from a societal perspective, while in countries such as the UK or Belgium, the healthcare payer's perspective is preferred. To make results comparable to other countries we also calculated the base case ICER from a healthcare payer's perspective, by excluding the indirect costs.</td></tr>
<tr><td>3</td><td>Base case analysis with dalteparin dose of 12,500 IU</td><td rowspan="2">The costs of dalteparin vary with the patient's weight. For the base case analysis, we assumed an average weight between 69 and 82 kg. In scenarios 3 and 4 we calculated the base case ICER with the costs of dalteparin based on weight categories of 57–68 kg (12,500 IE daily during month one followed by 10,000 IE daily in month two to six) and 83–98 kg (18,000 IE daily during month one followed by 15,000 IE daily in month two to six), respectively.</td></tr>
<tr><td>4</td><td>Base case analysis with dalteparin dose of 18,000 IU</td></tr>
</table>

For peer review only

5	Scenario 1 with treatment duration based on Streiff et al.	This scenario was similar to scenario 1, except for the treatment period which was based on a study of Streiff et al., who—comparable to SELECT-D—compared rivaroxaban to LMWH for the prevention of recurrent VTE in cancer patients [15]. They found an average treatment duration of one month and three months for LMWH and rivaroxaban, respectively.
6	Base case analysis using drug-specific distributions for the types of VTE and MB	Due to low numbers of VTE and MB events observed in the SELECT-D trial [14] and HOKUSAI VTE Cancer [16] trials, respectively, we calculated the distribution of the types of VTE and MB in the base case analysis based on the total number of events and assumed it to be equal for both drugs. In this scenario we assess the effect of this assumption on the cost-effectiveness results by using the drug-specific distributions of the types of VTE and MB based on the results of the SELECT-D and HOKUSAI VTE Cancer trials [14,19].

Abbreviations: IU, international units; MB, major bleeding; VTE, venous thromboembolism.

Budget impact

A budget impact analysis was conducted to estimate the total annual financial consequences of the implementation of rivaroxaban for the treatment and prevention of VTE in cancer patients within the Dutch healthcare setting. The budget impact was calculated from a societal perspective using the costs calculations from the cost-effectiveness model with a one-year time horizon. We extracted from the model the costs (event-related, treatment, and indirect costs) per patient with a cut-off point of one year for rivaroxaban and dalteparin. The difference in cost per patient was multiplied by the annual number of cancer patients with VTE in the Netherlands. The incidence of VTE in cancer patients and the total number of Dutch cancer patients were used to calculate the yearly number of cancer patients with VTE. The Netherlands Cancer Registry estimated a total of 579,781 cancer patients in 2017 [31]. The incidence of VTE in cancer patients was 13.9 per 1,000 person-years, based on a cohort study of linked UK databases [32]. The outcome of the budget impact analysis was presented as the total budget impact per year, including a subdivision of the costs per type (event-related costs, treatment costs and indirect costs) and corresponding 95% CIs derived from PSA.

Results

Cost-effectiveness analysis

Table 3 represents the deterministic results of the base case and scenario analyses. In each scenario, rivaroxaban was economically dominant–meaning that it simultaneously confers better clinical and quality-of-life outcomes at less cost–over dalteparin. As such, a numerical ICER is not presented because it has no meaning. Despite the fact that every scenario shows an improvement in the patient's health, the difference in QALYs was very low (incremental QALYs of 0.012 over 5 years' time horizon, which equals 4.4 quality-adjusted life days, in the base case analysis). In the base case analysis, rivaroxaban saved €1,376 per patient compared with dalteparin. The scenario calculating the cost-effectiveness over a six-month time horizon resulted in cost-savings of €1,312 per patient (scenario 1). There was increased cost-savings compared with the societal perspective when calculated from a healthcare payer's perspective (scenario 2). In scenarios 3 and 4 we assessed the effect of variations in the patient's weight (and thus dalteparin dosing) on the ICER. Compared with the base case analysis, there was decreased cost-savings with a lower dalteparin dose and increased cost-savings with a higher dalteparin dose, both still resulting in dominant ICERs. When comparing three months of rivaroxaban

For peer review only - http://bmjopen.bmj.com/site/about/guidelines.xhtml

treatment to one month of dalteparin treatment, we found incremental QALYs of 0.016 and cost-savings of €702 per patient (scenario 5). We assessed the effect of using drug-specific distributions of the types of VTE and MB, resulting in cost-savings of €1,815 and incremental QALYs of 0.037 (scenario 6).

The number of events and the corresponding average costs per patient in the base case analysis and scenario 4 (base case analysis with a time horizon of 6 months) are presented in Table 4. Rivaroxaban is associated with a lower number of recurrent VTE events, preventing on average €131 and €108 in costs per patient over five years and over six months, respectively. On the other hand, rivaroxaban causes more bleeding events, especially in the treatment period. ICH and non-ICH MB have the highest incremental event costs per patient. Treatment costs are higher for dalteparin compared with rivaroxaban, with incremental costs of €1,721 and €1,468 in the five-year and the six-month time horizon, respectively. The differences in indirect costs for rivaroxaban compared with dalteparin were €19 and -€2 for the five-year and the six-month time horizon, respectively.

For peer review only

Table 3. Deterministic results per patient of the base case and scenario analyses in a cohort of 1,000 cancer patients (2019, Euros).

	Costs	QALYs	Δ Costs	Δ QALYs	ICER
**Base case analysis** - 5-year time horizon from societal perspective					
Rivaroxaban	€3,139	2.459	-€1,476	0.012	Dominant
Dalteparin	€4,615	2.448			
**Scenario 1** – 6-month time horizon from societal perspective					
Rivaroxaban	€1,361	0.304	-€1,312	0.004	Dominant
Dalteparin	€2,673	0.300			
**Scenario 2** – base case analysis from healthcare payer's perspective					
Rivaroxaban	€2,942	2.459	-€1,496	0.012	Dominant
Dalteparin	€4,438	2.448			
**Scenario 3** – base case analysis with dalteparin dose of 12,500 IU					
Rivaroxaban	€3,139	2.459	-€1,079	0.012	Dominant
Dalteparin	€4,218	2.448			
**Scenario 4** – base case analysis with dalteparin dose of 18,000 IU					
Rivaroxaban	€3,139	2.459	-€1,898	0.012	Dominant
Dalteparin	€5,037	2.448			
**Scenario 5** – scenario 1 with treatment duration based on Streiff et al.					
Rivaroxaban	€1,299	0.289	-€702	0.016	Dominant
Dalteparin	€2,001	0.273			
**Scenario 6** – base case analysis using drug-specific distributions for the types of VTE and MB					
Rivaroxaban	€3,065	2.463	-€1,815	0.037	Dominant
Dalteparin	€4,880	2.425			

Abbreviations: ICER, incremental cost-effectiveness ratio; IU, international units; MB, major bleeding; QALY, quality adjusted life-years; VTE, venous thromboembolism.

Table 4. Number of events and costs per event per patient in a cohort of 1,000 cancer patients (2019, Euros).

Base case (5-year time horizon)						
	Rivaroxaban		Dalteparin		Incremental	
	Number of events	Costs per patient	Number of events	Costs per patient	Number of events	Costs per patient
**Event costs**						
Recurrent VTE	191	€311.85	275	€442.92	-84	-€131
Non-fatal symptomatic recurrent PE	33	€168.36	48	€239.13	-15	-€71

For peer review only - http://bmjopen.bmj.com/site/about/guidelines.xhtml

Non-fatal incidental recurrent PE	58	-	84	-	-26	
Non-fatal recurrent DVT	83	€59.31	120	€84.23	-37	-€25
Fatal recurrent VTE	17	€84.18	24	€119.56	-7	-€35
ICH	11	€550.70	9	€438.40	2	€112
Non-ICH MB	98	€1,106.87	79	€902.47	19	€204
Fatal MB	5	€51.48	4	€41.98	1	€10
CRNMB	197	€56.28	92	€26.93	105	€29
PTS	61	€92.72	61	€92.37	0	€0
CTEPH	20	€223.79	20	€222.83	0	€1
**Total event costs**		€2,705.54		€2,610.83		€95
**Treatment costs**		€548.83		€2,270.33		-€1,721
**Indirect costs**		€196.31		€177.08		€19
**Scenario 1 (6-month time horizon)**						
	**Rivaroxaban**		**Dalteparin**		**Incremental**	
	Number of events	Costs per patient	Number of events	Costs per patient	Number of events	Costs per patient
**Event costs**						
Recurrent VTE	38	€58.95	109	€166.96	-70	-€108
Non-fatal symptomatic recurrent PE	7	€31.82	19	€90.14	-12	-€58
Non-fatal incidental recurrent PE	12	-	33	-	-21	-
Non-fatal recurrent DVT	17	€11.21	47	€31.75	-31	-€21
Fatal recurrent VTE	3	€15.91	9	€45.07	-6	-€29
ICH	6	€142.82	4	€94.25	2	€49
Non-ICH MB	50	€539.38	33	€355.95	17	€183
Fatal MB	2	€25.09	2	€16.56	1	€9
CRNMB	130	€35.99	38	€10.62	91	€25
PTS	14	€20.59	14	€20.56	0	€0
CTEPH	3	€21.96	3	€21.93	0	€0
**Total event costs**		€903.72		€2,639.25		-€1,736
**Treatment costs**		€479.40		€1,947.45		-€1,468
**Indirect costs**		€36.50		€38.39		-€2

Abbreviations: CRNMB, clinically relevant non-major bleeding; CTEPH, chronic thromboembolic pulmonary hypertension; DVT, deep vein thrombosis; ICH, intracranial haemorrhage; MB, major bleeding; PE, pulmonary embolism; PTS, post thrombotic syndrome; VTE, venous thromboembolism

In the probabilistic sensitivity analysis, we assessed the robustness of the model over a five-year time horizon (base case) and a six-month time horizon (scenario 1). The results are presented in cost-effectiveness planes in Figure 2 and Figure S1. In the base case analysis, rivaroxaban was in the majority (77.8%) of the 2,000 iterations cost-saving and more effective compared with dalteparin. In 22.2% of the iterations rivaroxaban is considered cost-saving but less effective compared with dalteparin. In scenario 1, rivaroxaban was in almost all (98.7%) the iterations cost-saving and more effective compared with dalteparin.

The influence of the individual input parameters on the base case incremental costs and QALYs are analysed in the univariate sensitivity analysis. The tornado diagrams (Figure 3 and Figure 4) present the 12 input parameters with the highest impact in the base case analysis. The risk of MB for both rivaroxaban and dalteparin, treatment duration of dalteparin, and recurrent VTE risks during the first six months after a VTE had the highest influence on the incremental costs. Similarly, the risk of MB and recurrent VTE in the first six months for rivaroxaban and dalteparin showed the highest influence on the incremental QALYs. Similar results were found in the univariate sensitivity analysis of scenario 1 (Figure S2 and Figure S3).

For peer review only - http://bmjopen.bmj.com/site/about/guidelines.xhtml

For peer review only

Figure 2. Probabilistic sensitivity analysis of the base case with five-year time horizon (base case analysis). *The red mark represents the deterministic incremental cost-effectiveness ratio. Abbreviation: QALY, quality adjusted life-year*

Figure 3. Tornado diagram from the univariate sensitivity analysis for the base case analysis showing the impact of parameters on the incremental costs. *Abbreviations: ICH, intracranial haemorrhage; MB, major bleeding; PE, pulmonary embolism; VTE, venous thromboembolism*

Figure 4. Tornado diagram from the univariate sensitivity analysis for the base case analysis showing the impact of parameters on the incremental QALYs. *Abbreviations: CRNMB, clinically relevant non-major bleeding; ICH, intracranial haemorrhage; MB, major bleeding; VTE, venous thromboembolism*

Budget impact

The results of the budget impact analysis are presented in Table 5. The replacement of LMWHs (including dalteparin) with rivaroxaban can lead to cost-savings of a maximum of €11,326,763 (€5,164,254–€17,363,231) over approximately 8,000 cancer patients with VTE based on a one-year time horizon. A reduction in treatment costs can lead to savings of up to €12.6 million. Event-related costs and indirect costs slightly increase by €1,234,467 (€-2,103,366–€5,231,955) and €2,101 (€-173,830–€184,677), respectively, when LMWHs are replaced by rivaroxaban.

Table 5. Budget impact over one-year time horizon in the Netherlands.

Event-related costs	€1,234,467 (€-2,103,366–€5,231,955)
Treatment costs	€-12,559,130 (€-17,327,405–€-8,149,498)
Indirect costs	€-2,101 (€-173,830–€184,677)
**Budget impact**	**€-11,326,763 (€-17,363,231–€-5,164,254)**

Discussion

Thrombosis treatment is a challenge in cancer patients. According to the guidelines, LMWHs and DOACs edoxaban and rivaroxaban are the preferred treatment for the prevention of recurrent VTE in cancer patients [8–11]. We have assessed the cost-effectiveness and budget impact of rivaroxaban in cancer patients at risk of recurrent VTE based on the SELECT-D trial [15]. We conclude that, in the Netherlands, rivaroxaban is a cost-saving treatment option with a small health benefit per patient over five years compared with dalteparin. In sensitivity analyses our model appeared to be robust.

The cost-savings were mainly driven by the difference in treatment costs. It should be noted that this is specifically the case for the Netherlands, and may differ in other countries. On the other hand, both the cost-effectiveness and budget impact analyses showed that the event-related costs and indirect costs increase with the use of rivaroxaban compared with dalteparin. A total of 84 VTE-related events were prevented over five years, leading to an average cost-saving of €131 per patient. This is line with findings from a recent study that assessed the VTE-related healthcare costs in cancer patients,

For peer review only - http://bmjopen.bmj.com/site/about/guidelines.xhtml

which found that rivaroxaban treated patients had a significantly lower total VTE-related costs relative to patients treated with LMWH [33]. Although the cost difference between the rivaroxaban and LWMH cohorts was even greater with $12,004 per patient per year.

On the other hand, MB events were more frequent with rivaroxaban compared with dalteparin (11 ICH and 98 non-ICH versus 9 ICH and 79 non-ICH, respectively). MB events are very burdensome and frequently severely disabling, leading to high acute and long-term direct and indirect costs. This explains why the indirect costs were higher for rivaroxaban than for dalteparin in the base case scenario. Moreover, there was no data available on leisure activity losses caused by the occurrence of a VTE event in patients who are already burdened with cancer. Therefore, the indirect costs might have been underestimated, possibly leading to lower cost-saving results. The indirect costs account for €196 to €177 per patient over five years—approximately 4-6% of the total cost—however, they do not have a major influence on the differences between the two drugs (€19 and -€2 for the five-year and 6-month time horizon, respectively). This suggests that, although the indirect costs might have been underestimated, rivaroxaban is still likely to be cost-saving compared with dalteparin.

As mentioned, the main driver of the cost-savings is the difference in treatment costs. In the cost-effectiveness analysis, we estimated that more than €1,700 per patient over a five-year period can be saved on treatment costs, compared with dalteparin. Moreover, in the scenario analysis we varied the price of dalteparin based on weight. Although the lowest dose (12,500 IU daily during month one followed by 10,000 IU in months two to six based on weight class 57–68 kg) had a lower price, €8.06 versus €9.93, the ICER remained cost-saving. Rivaroxaban users were assumed to require an annual check-up of their renal function. However, cancer patients (especially those with metastatic cancer) are at higher risk for renal impairment and may be tested much more frequently [34]. This may have caused an overestimation of the costs of rivaroxaban, and therefore underestimated the total cost-savings of rivaroxaban compared with dalteparin.

In the budget impact analysis, we calculated that rivaroxaban replacing LMWH (including dalteparin) leads to cost-savings of a maximum of €11,326,763 within one year over a total of 8,000 cancer patients. This is the absolute maximum, since it is not possible to treat each patient with rivaroxaban from a clinical perspective. In practice, the market share of rivaroxaban will be lower—despite the fact that there are three other DOACs that could be prescribed—because there are some clinical considerations that should be taken into account. Firstly, although DOACs have far fewer drug interactions than VKAs, it should be noted that rivaroxaban is metabolized by CYP3A4 enzymes [1]. Cancer patients, especially those with haematological cancer, are at high risk for opportunistic and fungal infections, for which they are often treated with CYP3A4 inhibitors or inducers [35]. For this reason, prescription of rivaroxaban for the prevention of recurrent VTE in cancer patients must be done carefully [1]. This interaction does not play a role in LWMH treatment.

Secondly, the balance between the risk of thrombosis and the risk of bleeding should always be a consideration in the prescription of anticoagulants. For example, DOACs are not advised in patients with GI tumours, due to a higher risk of GI bleeding [8–11]. Some prediction scores for primary prevention have been developed to predict thrombosis risk in cancer patients, since thrombosis prophylaxis is most effective in patients with an increased VTE risk. Unfortunately, for cancer these scores have still not been shown to reliably identify patients with the highest risk [36]. Predictive scores for bleeding, such as the HAS-BLED score used for atrial fibrillation patients, are also needed.

A third consideration is the oral administration of rivaroxaban. Although it is less burdensome than the LMWH injections, oral administration can be problematic in patients with anorexia and vomiting, which is often seen as a side effect in cancer therapy [15]. Moreover, low food intake might

For peer review only - http://bmjopen.bmj.com/site/about/guidelines.xhtml

influence the metabolism of rivaroxaban resulting in lower bioavailability [37]. Lastly, adherence is always a point of discussion, but since adherence to current guidelines is often low [36], we feel that adherence might even increase due to the more patient-friendly administration.

Our analysis is not without limitations. It should also be noted that 58% of the patients included in the SELECT-D trial were having metastatic cancer, and thus results and conclusions pertain mostly to severely ill patients. Also, the majority (53%) of the initial VTE events were incidental PE, related to computed tomography imaging for tumour status [15]. Additionally, as with all cost-effectiveness models some assumptions need to be made due to lack of data.

We assumed that patients were treated with anticoagulation over six months, which is in line with the guidelines [8–11]. Previous studies have shown that adherence to these guidelines is poor [36]. As seen in the study by Streiff et al, in practice, treatment with LMWH is often not six months, presumably due to the fact that LMWH injections are burdensome, there are concerns about the bleeding risk, and the complexity of the treatment of cancer patients [36]. However, this recommended treatment period was also not achieved in many patients treated with rivaroxaban, which resulted in an average duration of three months. We conducted a scenario analysis (scenario 5) to assess this difference in treatment duration (one month of LMWH versus three months of rivaroxaban). These results favoured rivaroxaban, because the incremental QALYs increased while still being cost-saving. On the other hand, there are also some clinical situations in which the treatment period might be longer than six months: for example, in patients with a recurrent VTE event, patients with an active malignancy, or patients receiving cancer treatment for their malignancy beyond six months. Moreover, in the Netherlands anticoagulation is often continued after six months of initial treatment in case the cancer is still active. Unfortunately, we were unable to assess the effect of continued anticoagulation treatment due to lack of data. However, since rivaroxaban is associated with cost-saving results during the first six months, it is to be expected that during a longer treatment period the cost-savings and health gains will accrue even more compared with dalteparin.

In the univariate sensitivity analysis, we have shown that the risk of MB and VTE for both rivaroxaban and dalteparin have a high influence on the incremental costs and QALYs. In the SELECT-D trial [15], the incidence of symptomatic and fatal PE events was relatively higher in patients treated with rivaroxaban. However, due to low numbers of VTE observed in the SELECT-D trial [15], we calculated the distribution of the type of VTE based on the total number of events and assumed it to be equal for both drugs. This may have led to an overestimation of the effect of rivaroxaban compared with dalteparin, since symptomatic and fatal PE events have a higher impact on the costs and the patient’s health compared to DVT and incidental PE. On the other hand, we used this same approach to calculate the distributions of the types of MB from the HOKUSAI VTE Cancer trial [16], in which the patients treated with dalteparin had relatively more severe MB events compared with the NOAC edoxaban (ICH: 17.6% versus 6.1%, respectively). This results in an underestimation of the number of MBs in dalteparin-treated patients. We assessed the effect of using drug-specific distributions of the type of VTE and MB in scenario six, showing an increase in incremental cost-savings and QALYs compared to the base case analysis. Therefore, we conclude that our approach of using equal distributions of the types of VTE and MB for rivaroxaban and dalteparin is conservative.

This study focuses on the secondary prevention of VTE, based on the results of the SELECT-D and, partially, the HOKUSAI VTE Cancer trials. However, recently, apixaban was also assessed in cancer patients at risk of recurrent VTE and found to be non-inferior compared to dalteparin [38,39]. Moreover, the AVERT and CASSINI trials have shown that apixaban and rivaroxaban are also effective as a primary prophylaxis of VTE in cancer patients compared with a placebo [40–42]. Based on these

For peer review only

For peer review only - http://bmjopen.bmj.com/site/about/guidelines.xhtml

two studies, clinicians may consider DOAC prophylaxis in some of their cancer patients [42]. Therefore, future research is needed to assess if DOACs are also cost-effective for the primary prevention of VTE.

Conclusion

Treatment with rivaroxaban is dominant (cost-saving while slightly improving the patient's health) over dalteparin in cancer patients at risk for recurrent VTE in the Netherlands. The use of rivaroxaban instead of LMWH can save more than eleven million euros per year, which is primarily driven by the difference in treatment costs. Since treatment with rivaroxaban is economically dominant compared with dalteparin and its oral administration is more convenient than daily subcutaneous injection, we feel that certain cancer patients can benefit from DOAC treatment.

References

1. Gerotziafas GT, Mahé I, Elalamy I. Therapeutics and Clinical Risk Management Dovepress New orally active anticoagulant agents for the prevention and treatment of venous thromboembolism in cancer patients. Ther Clin Risk Manag. 2014;10:423–36.
2. Falanga A, Marchetti M, Vignoli A. Coagulation and cancer: Biological and clinical aspects. J Thromb Haemost. 2013;11(2):223–33.
3. Lechner D, Weltermann A. Chemotherapy-induced thrombosis: a role for microparticles and tissue factor? Semin Thromb Hemost. 2008;34(2):199–203.
4. Cohen AT, Katholing A, Rietbrock S, Bamber L, Martinez C. Epidemiology of first and recurrent venous thromboembolism in patients with active cancer. Thromb Haemost. 2017;117(01):57–65.
5. Minister of Health W and S. Letter to the parlement - reimbursement rivaroxaban VTE (Herbeoordeling uitbreiding nadere voorwaarden rivaroxaban (Xarelto®) bij VTE. 2015;
6. Steffel J, Verhamme P, Potpara TS, Albaladejo P, Antz M, Desteghe L, et al. The 2018 European Heart Rhythm Association Practical Guide on the use of non-vitamin K antagonist oral anticoagulants in patients with atrial fibrillation. Eur Heart J. 2018;
7. Mandala M, Falanga A, Roila F, ESMO Guidelines Working Group. Management of venous thromboembolism (VTE) in cancer patients: ESMO Clinical Practice Guidelines. Ann Oncol. 2011;22(Supplement 6):vi85–92.
8. Key NS, Khorana AA, Kuderer NM, Bohlke K, Lee AYY, Arcelus JI, et al. Venous thromboembolism prophylaxis and treatment in patients with cancer: ASCO clinical practice guideline update. J Clin Oncol. 2020;38(5):496–520.
9. Farge D, Frere C, Connors JM, Ay C, Khorana AA, Munoz A, et al. 2019 International Clinical Practice Guidelines for the Treatment and Prophylaxis of Venous Thromboembolism in Patients With Cancer. Lancet Oncol. 2019;20(10):e566–81.
10. Konstantinides S V., Meyer G, Bueno H, Galié N, Gibbs JSR, Ageno W, et al. 2019 ESC Guidelines for the diagnosis and management of acute pulmonary embolism developed in collaboration with the European respiratory society (ERS). Eur Heart J. 2020;41(4):543–603.
11. Streiff MB, Holmstrom B, Angelini D, Ashrani A, Bockenstedt PL, Chesney C, et al. NCCN Guidelines® insights cancer-associated venous thromboembolic disease, version 2.2018 featured updates to the NCCN guidelines. JNCCN J Natl Compr Cancer Netw. 2018;16(11):1289–303.
12. Hull RD, Pineo GF, Brant RF, Mah AF, Burke N, Dear R, et al. Long-term low-molecular-weight heparin versus usual care in proximal-vein thrombosis patients with cancer. Am J Med. 2006

For peer review only - http://bmjopen.bmj.com/site/about/guidelines.xhtml

Dec;119(12):1062–72.
13. Lee AYY, Levine MN, Baker RI, Bowden C, Kakkar AK, Prins M, et al. Low-Molecular-Weight Heparin versus a Coumarin for the Prevention of Recurrent Venous Thromboembolism in Patients with Cancer. N Engl J Med. 2003;349(2):146–53.
14. Meyer G, Marjanovic Z, Valcke J, Lorcerie B, Gruel Y, Solal-Celigny P, et al. Comparison of low-molecular-weight heparin and warfarin for the secondary prevention of venous thromboembolism in patients with cancer: a randomized controlled study. Arch Intern Med. 162(15):1729–35.
15. Young AM, Marshall A, Thirlwall J, Chapman O, Lokare A, Hill C, et al. Comparison of an Oral Factor Xa Inhibitor With Low Molecular Weight Heparin in Patients With Cancer With Venous Thromboembolism: Results of a Randomized Trial (SELECT-D). J Clin Oncol. 2018;36(20):2017–23.
16. Raskob GE, van Es N, Verhamme P, Carrier M, Di Nisio M, Garcia D, et al. Edoxaban for the Treatment of Cancer-Associated Venous Thromboembolism. N Engl J Med. 2018;378(7):615–24.
17. Streiff MB, Milentijevic D, McCrae K, Yannicelli D, Fortier J, Nelson WW, et al. Effectiveness and safety of anticoagulants for the treatment of venous thromboembolism in patients with cancer. Am J Hematol. 2018;93(5):664–71.
18. Dutch Institute National Health Care (Zorginstituut Nederland). Richtlijn voor het uitvoeren van economische evaluaties in de gezondheidzorg (Protocol for the execution of economic evaluation in healthcare). 29-02-2016. 2016;(november):120.
19. National Institute of Public Health and the Environment (RIVM). Cancer numbers and context: current situation [Internet]. volksgezondheidenzorg.info. 2017. p. 1.
20. Briggs A, Claxton K, Sculpher M. Decision modelling for Health Economic Evaluation. 3rd ed. New York: Oxford University Press; 2011. 57–58 p.
21. The Netherlands Cancer Registry. Survival statistics - all tumours 1961-2015 [Internet].
22. Heisen M, Treur MJ, Heemstra HE, Giesen EBW, Postma MJ. Cost-effectiveness analysis of rivaroxaban for treatment and secondary prevention of venous thromboembolism in the Netherlands. J Med Econ. 2017 Aug 3;20(8):813–24.
23. Medicijnkosten.nl. The National Health Care Institute (ZIN) [Internet].
24. Summary of Product Characteristics Fragmin. 2015;
25. Dutch Statistics. Length and weight of Dutch population [Internet]. 2017.
26. de Klerk M, de Boer A, Plaisier I, Schyns P, Kooiker S. Informele hulp: wie doet er wat? - Rapport - SCP [Internet]. 2015-35. 2015.
27. Hakkaart-van Roijen L, van der Linden N, Bouwmans C, Kanters T, Swan Tan S. Kostenhandleiding: Methodologie van kostenonderzoek en referentieprijzen voor economische evaluaties in de gezondheidszorg. Zorginstituut Ned. 2016;1–120.
28. Lloyd AJ, Dewilde S, Noble S, Reimer E, Lee AYY. What Impact Does Venous Thromboembolism and Bleeding Have on Cancer Patients' Quality of Life? Value Heal. 2018;21(4):449–55.
29. Roman A, Barbera JA, Castillo MJ, Muñoz R, Escribano P. Health-related quality of life in a national cohort of patients with pulmonary arterial hypertension or chronic thromboembolic pulmonary hypertension. Arch Bronconeumol. 2013;49(5):181–8.
30. Stevanović J, de Jong LA, Kappelhoff BS, Dvortsin EP, Voorhaar M, Postma MJ. Dabigatran for the Treatment and Secondary Prevention of Venous Thromboembolism; A Cost-Effectiveness Analysis for the Netherlands. PLoS One. 2016;11(10):e0163550.
31. The Netherlands Cancer Registry. Prevalence statistics - all tumours [Internet].
32. Walker AJ, Card TR, West J, Crooks C, Grainge MJ. Incidence of venous thromboembolism in patients with cancer - a cohort study using linked United Kingdom databases. Eur J Cancer. 2013 Apr;49(6):1404–13.
33. Streiff M, Milentijevic D, McCrae KR, Laliberté F, Lejeune D, Lefebvre P, et al. Healthcare resource utilization and costs associated with venous thromboembolism in cancer patients

For peer review only - http://bmjopen.bmj.com/site/about/guidelines.xhtml

treated with anticoagulants. J Med Econ. 2019;22(11):1134–40.
34. Aapro M, Launay-Vacher V. Importance of monitoring renal function in patients with cancer. Cancer Treat Rev. 2012;38(3):235–40.
35. Vedham V, Divi RL, Starks VL, Verma M. Multiple Infections and Cancer: Implications in Epidemiology. Technol Cancer Res Treat. 2014;13(2):177–94.
36. Mahé I, Chidiac J, Helfer H, Noble S. Factors influencing adherence to clinical guidelines in the management of cancer-associated thrombosis. J Thromb Haemost. 2016;14(11):2107–13.
37. Committee for Medicinal Products for Human Use (CHMP). Summary of product characteristics - rivaroxaban 20 mg [Internet].
38. Agnelli G, Becattini C, Meyer G, Muñoz A, Huisman M V., Connors JM, et al. Apixaban for the treatment of venous thromboembolism associated with cancer. N Engl J Med. 2020;382(17):1599–607.
39. McBane RD, Wysokinski WE, Le-Rademacher JG, Zemla T, Ashrani A, Tafur A, et al. Apixaban and dalteparin in active malignancy-associated venous thromboembolism: The ADAM VTE trial. J Thromb Haemost. 2020;18(2):411–21.
40. Khorana AA, Soff GA, Kakkar AK, Vadhan-Raj S, Riess H, Wun T, et al. Rivaroxaban for Thromboprophylaxis in High-Risk Ambulatory Patients with Cancer. N Engl J Med. 2019 Feb;380(8):720–8.
41. Carrier M, Abou-Nassar K, Mallick R, Tagalakis V, Shivakumar S, Schattner A, et al. Apixaban to Prevent Venous Thromboembolism in Patients with Cancer. N Engl J Med. 2019;380(8):711–9.
42. Agnelli G. Direct Oral Anticoagulants for Thromboprophylaxis in Ambulatory Patients with Cancer. N Engl J Med. 2019;380(8):781–3.

Author contributions

Lisa A. de Jong built the economic model, performed the analyses, and contributed to the design of the work, interpretation of the results, writing of the manuscript.
Annette W.G. van der Velden contributed to the interpretation of the results, writing of the manuscript, and critical revision for important intellectual content.
Marinus van Hulst contributed to the design of the work, interpretation of the results, writing of the manuscript, and critical revision for important intellectual content.
Maarten J. Postma contributed to the design of the work, interpretation of the results, writing of the manuscript, and critical revision for important intellectual content.
All authors approved of the version to be published.

Checklist for the appropriate reporting statement: This manuscript was written in accordance with the CHEERS checklist for reporting economic evaluations of health interventions.

Data sharing statement: All relevant data are included in the manuscript. The analyses were conducted based on publicly available information which is presented and referenced in the manuscript.

Word count: 4,508 (excluding table and figure descriptions and references)

Patient consent and ethical approval: The analyses were conducted based on publicly available information which is presented and referenced in the article and Supporting Information files, and did therefore not require any patient consent forms or approval from an ethical review board.

For peer review only - http://bmjopen.bmj.com/site/about/guidelines.xhtml

Patient and public involvement statement: It was not appropriate to involve patients or the public in the design, or conduct, or reporting, or dissemination plans of our research, because this health economic analysis was based on publicly available data and solely concentrated on the analysis of the economics consequence of treating cancer patients with rivaroxaban instead of the current standard of care.

For peer review only

For peer review only - http://bmjopen.bmj.com/site/about/guidelines.xhtml

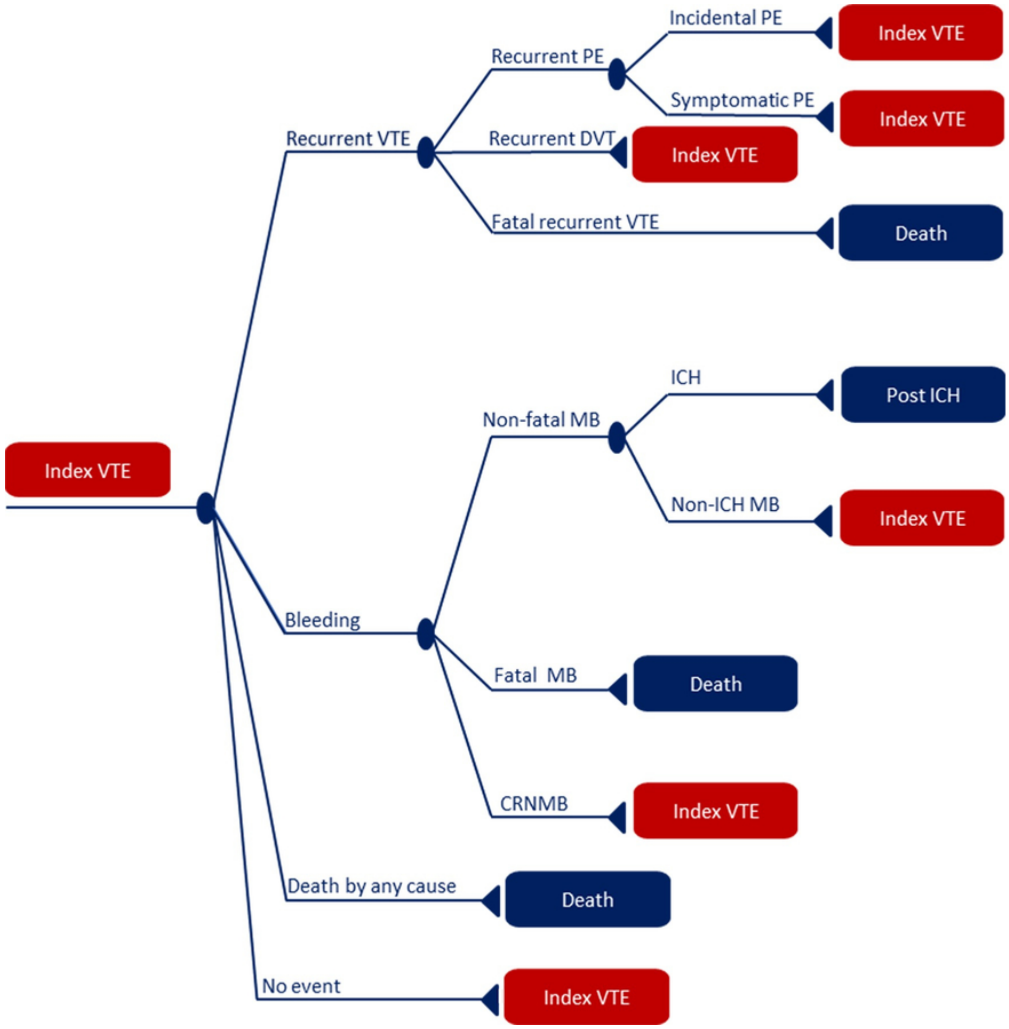

Figure 1. Model outline. All patients enter the model in the 'Index VTE' state and move to other states upon the occurrence of one of the following events: recurrent incidental PE, recurrent symptomatic PE, fatal recurrent VTE, recurrent DVT, ICH, non-ICH MB, fatal MB, CRNMB, or death by any cause. The triangles represent the health state a patient will enter after an event. The blue squares are permanent states, in which a patient will remain until death while not being at risk for other events. The red squares represent a transient state: the patient will re-enter the model in the 'Index VTE' state.
Abbreviations: CRNMB, clinically relevant non-major bleeding; DVT, deep vein thrombosis; ICH, intracranial haemorrhage; MB, major bleeding; PE, pulmonary embolism; VTE, venous thromboembolism

74x75mm (300 x 300 DPI)

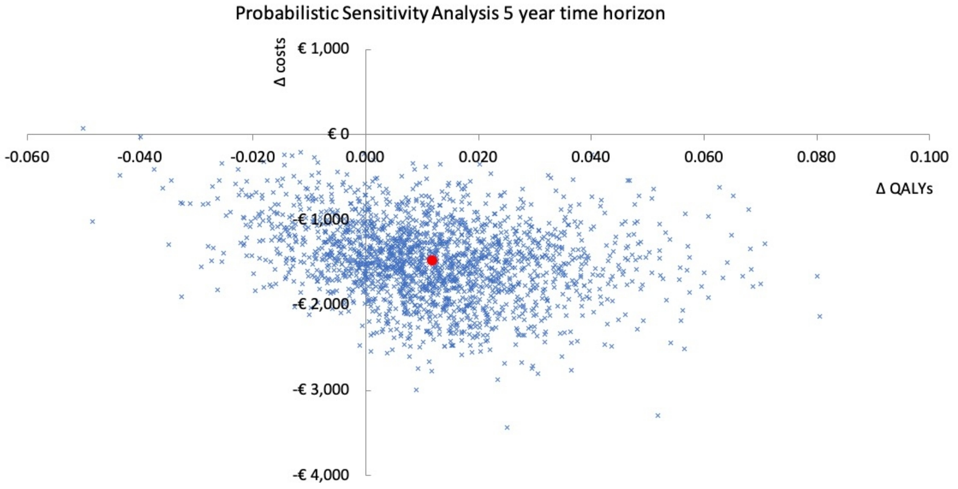

Figure 2. Probabilistic sensitivity analysis of the base case with five-year time horizon (base case analysis). The red mark represents the deterministic incremental cost-effectiveness ratio. Abbreviation: QALY, quality adjusted life-year

206x112mm (144 x 144 DPI)

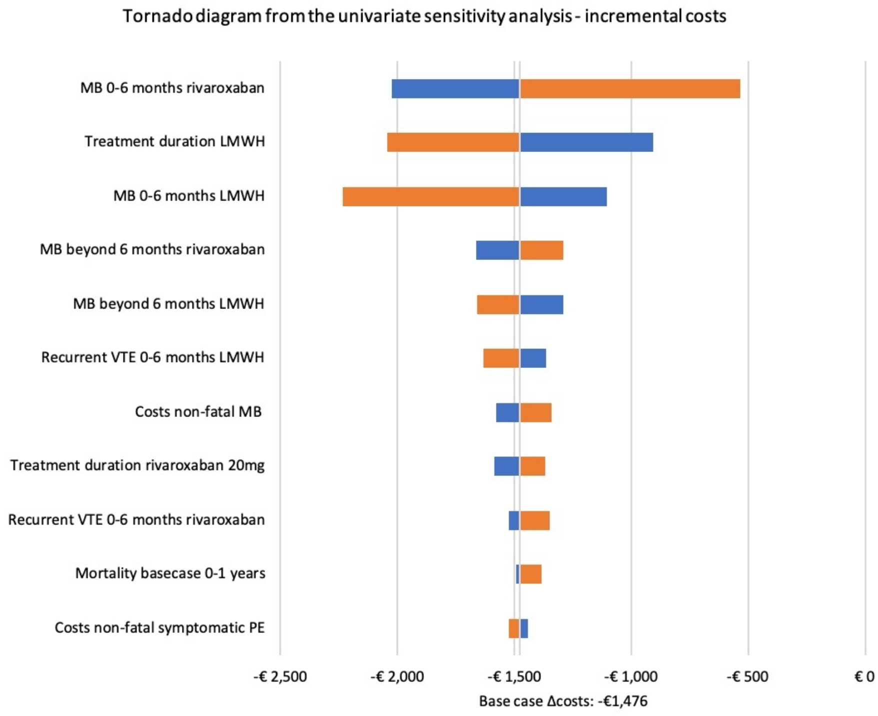

Figure 3. Tornado diagram from the univariate sensitivity analysis for the base case analysis showing the impact of parameters on the incremental costs. Abbreviations: ICH, intracranial haemorrhage; MB, major bleeding; PE, pulmonary embolism; VTE, venous thromboembolism

194x143mm (144 x 144 DPI)

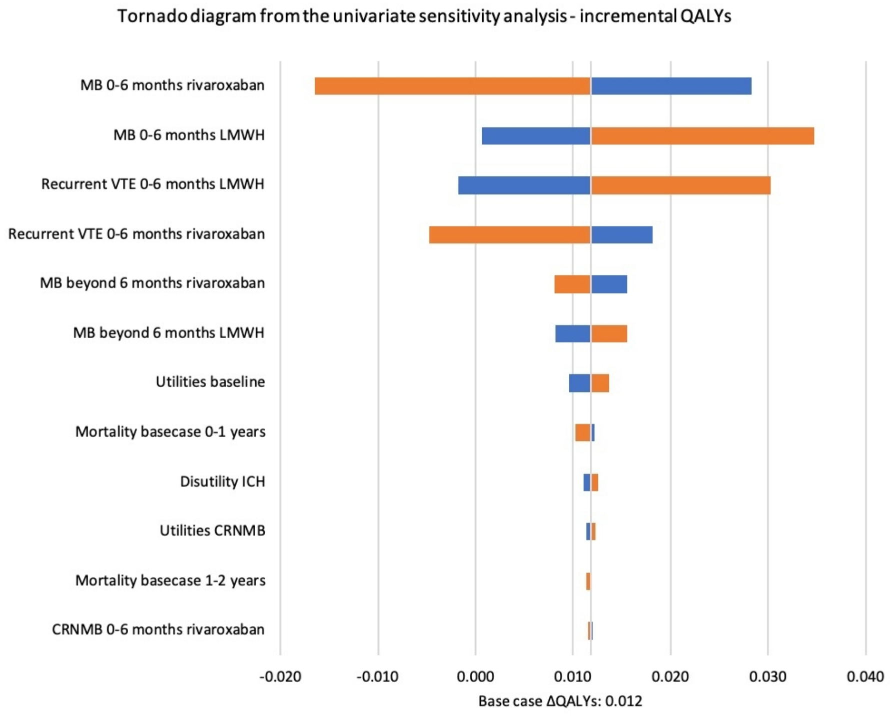

Figure 4. Tornado diagram from the univariate sensitivity analysis for the base case analysis showing the impact of parameters on the incremental QALYs. Abbreviations: CRNMB, clinically relevant non-major bleeding; ICH, intracranial haemorrhage; MB, major bleeding; VTE, venous thromboembolism

194x143mm (144 x 144 DPI)

Supplementary data file – Table S1

Manuscript title: Cost-effectiveness analysis and budget impact of rivaroxaban in cancer patients at risk of recurrent venous thromboembolism

Table S1. Transition probabilities used in the cost-effectiveness model

	Rivaroxaban (95% CI)	LMWH (95% CI)	Distribution	Reference
**Recurrent VTE**				
0–6 months	0.040 (0.020 – 0.090)	0.110 (0.070 – 0.160)	Beta	[1]
6–12 months	0.040 (0.031 – 0.050)		Beta	[2]
1–2 years	0.034 (0.027 – 0.042)		Beta	[2]
2–3 years	0.021 (0.014 – 0.029)		Beta	[2]
3–4 years	0.016 (0.009 – 0.026)		Beta	[2]
4–5 months	0.013 (0.006 – 0.024)		Beta	[2]
**Type of recurrent VTE**				
Symptomatic PE	17.4% (α = 4, β = 19)		Dirichlet	[1]
Incidental PE	30.4% (α = 7, β = 16)		Dirichlet	[1]
DVT	43.5% (α = 10, β = 13)		Dirichlet	[1]
Fatal PE	8.7% (α = 2, β = 21)		Dirichlet	[1]
**MB**				
0–6 months	0.060 (0.030 – 0.110)	0.040 (0.020 – 0.080)	Beta	[1]
Beyond 6 months treatment	0.008 (0.006 – 0.010)		Beta	[3]
**Type of MB**				
ICH	10% (α = 5, β = 45)		Dirichlet	[3]
Non-ICH MB	86% (α = 43, β = 7)		Dirichlet	[3]
Fatal MB	4% (α = 2, β = 48)		Dirichlet	[3]
**CRNMB**				
0–6 months	0.130 (0.090 – 0.190)	0.040 (0.020 – 0.090)	Beta	[1]
Beyond 6 months treatment	0.008 (0.006 – 0.010)		Beta	[3]
**PTS**				
0–6 months	0.015 (0.011 – 0.019)		Beta	[4]
6–12 months	0.012 (0.009 – 0.015)		Beta	[4]
12–18 months	0.008 (0.006 – 0.010)		Beta	[4]
18–24 months	0.025 (0.023 – 0.019)		Beta	[4]
24–30 months	0.011 (0.008 – 0.014)		Beta	[4]
30–36 months	0.006 (0.005 – 0.008)		Beta	[4]
3–4 years	0.001 (0.0008 – 0.0013)		Beta	[4]
4–5 years	0.001 (0.0008 – 0.0013)		Beta	[4]
**CTEPH (annual risk)**	0.0057 (0.0002 – 0.012)		Beta	[5]
**Mortality (annual risk)**				
0–1 years	0.230 (0.200 – 0.390)		Beta	[6]
1–2 years	0.104 (0.088 – 0.180)		Beta	[6]
2–3 years	0.058 (0.055 – 0.120)		Beta	[6]
3–4 years	0.046 (0.043 – 0.068)		Beta	[6]
4–5 years	0.032 (0.030 – 0.073)		Beta	[6]
**Relative risk of recurrent VTE, MB, and CRNMB for LMWH versus placebo, used in scenario 5**				
Recurrent VTE (any)	5.170		Fixed	[7]
MB	0.242		Fixed	[7]
CRNMB	1.000		Fixed	[7]

For peer review only

Drug-specific distribution of the type of VTE, used in scenario 6				
Symptomatic PE	28.6% (α = 2, β = 5)	12.5% (α = 2, β = 14)	Dirichlet	[1]
Incidental PE	14.3% (α = 1, β = 6)	37.5% (α = 6, β = 10)	Dirichlet	[1]
DVT	42.9% (α = 3, β = 4)	43.8% (α = 7, β = 9)	Dirichlet	[1]
Fatal PE	14.3% (α = 1, β = 6)	6.3% (α = 1, β = 15)	Dirichlet	[1]
**Drug-specific distribution of the type of MB, used in scenario 6**				
ICH	6.1% (α = 2, β = 31)	17.6% (α = 3, β = 14)	Dirichlet	[3]
Non-ICH MB	93.9% (α = 31, β = 2)	70.6% (α = 12, β = 5)	Dirichlet	[3]
Fatal MB	0% (α = 0, β = 33)	11.8% (α = 2, β = 15)	Dirichlet	[3]

Abbreviations: CI, confidence interval; CRNMB, clinically relevant non-major bleeding; CTEPH, chronic thromboembolic pulmonary hypertension; DVT, deep vein thrombosis; ICH, intracranial haemorrhage; LMWH, low-molecular weight heparin; MB, major bleeding; PE, pulmonary embolism; PTS, post-thrombotic syndrome; SE, standard error; VTE, venous thromboembolism

For peer review only

References

1. Young AM, Marshall A, Thirlwall J, Chapman O, Lokare A, Hill C, et al. Comparison of an Oral Factor Xa Inhibitor With Low Molecular Weight Heparin in Patients With Cancer With Venous Thromboembolism: Results of a Randomized Trial (SELECT-D). J Clin Oncol. 2018;36(20):2017–23.
2. Cohen AT, Katholing A, Rietbrock S, Bamber L, Martinez C. Epidemiology of first and recurrent venous thromboembolism in patients with active cancer. Thromb Haemost. 2017;117(01):57–65.
3. Raskob GE, van Es N, Verhamme P, Carrier M, Di Nisio M, Garcia D, et al. Edoxaban for the Treatment of Cancer-Associated Venous Thromboembolism. N Engl J Med. 2018;378(7):615–24.
4. Prandoni P, Villalta S, Bagatella P, Rossi L, Marchiori A, Piccioli A, et al. The clinical course of deep-vein thrombosis. Prospective long-term follow-up of 528 symptomatic patients. Haematologica. 1997;82(4):423–8.
5. Klok FA, van Kralingen KW, van Dijk APJ, Heyning FH, Vliegen HW, Huisman M V. Prospective cardiopulmonary screening program to detect chronic thromboembolic pulmonary hypertension in patients after acute pulmonary embolism. Haematologica. 2010;95(6):970–5.
6. The Netherlands Cancer Registry. Survival statistics - all tumours 1961-2015 [Internet].
7. Napolitano M, Saccullo G, Malato A, Sprini D, Ageno W, Imberti D, et al. Optimal duration of low molecular weight heparin for the treatment of cancer-related deep vein thrombosis: the Cancer-DACUS Study. J Clin Oncol. 2014;32(32):3607–12.

Supplementary data file – Table S2

Manuscript title: Cost-effectiveness analysis and budget impact of rivaroxaban in cancer patients at risk of recurrent venous thromboembolism

Table S2. Costs included in the cost-effectiveness model (Euros, 2019)

	Value (95% CI)	Distribution	Reference
**Event costs**			
Recurrent VTE			
Symptomatic PE	€4,717 (€2,364 – €7,868)	Gamma	[1]
Incidental PE	€0	Fixed	Assumption
DVT	€663 (€464 – €862)	Gamma	[1]
Fatal recurrent VTE [a]	€4,717 (€2,364 – €7,868)	Gamma	[1]
ICH acute care costs	€22,769 (€11,644 – €31,175)	Gamma	[2]
ICH long-term costs (monthly)	€637 (€319 – €1,063)	Gamma	[1]
Non-ICH MB	€10,685 (€5,356 – €17,824)	Gamma	[1]
Fatal MB	€10,685 (€5,356 – €17,824)	Gamma	[1]
CRNMB	€274 (€137 – €457)	Gamma	[1]
PTS	€1,431 (€717 – €2,387)	Gamma	[1]
CTEPH acute care costs	€7,843 (€3,931 – €16,433)	Gamma	[1]
CTEPH long-term costs (monthly)	€89 (€45 – €149)	Gamma	[1]
**Treatment costs**			
Drug cost (daily)			
LMWH [b]	€9.93	Fixed	[3]
Rivaroxaban 15 mg	€4.58	Fixed	[3]
Rivaroxaban 20 mg	€2.29	Fixed	[3]
Treatment duration (days)			
LMWH	183 (137 – 228)	Gamma	[4]
Rivaroxaban 15 mg	21 (16 – 26)	Gamma	[4]
Rivaroxaban 20 mg	162 (121 – 202)	Gamma	[4]
LMWH administration costs			
Costs for home caregiver (per hour)	€59.34 (€44.51 – €74.18)	Gamma	[5]
Duration of at home administration (hour)	0.25 (0.19 – 0.31)	Gamma	Assumption
Hospitalisation duration PE (days) [c]	6.6 (5.0 – 8.3)	Gamma	[6]
Renal monitoring [c]	€1.64 (€1.23 – €2.05)	Gamma	[7]
**Indirect costs**			
Travel costs			
Cost per km	€0.20 (€0.15 – €0.25)	Gamma	[8]
Distance to hospital (km)	7	Fixed	[8]
Distance to GP (km)	1.1	Fixed	[8]
Informal care costs			
PE	€1,515 (€1,136 – €1,894)	Gamma	[5,9]
DVT	€233 (€175 – €291)	Gamma	[5,9]
ICH (acute informal care costs)	€1,515 (€1,136 – €1,894)	Gamma	[5,9]
ICH (long-term informal care costs, monthly)	€626 (€470 – €783)	Gamma	[10]
Non-ICH MB	€758 (€568 – €947)	Gamma	[5,9]
CRNMB	€117 (€87 – €146)	Gamma	[5,9]

Abbreviations: CI, confidence interval; CRNMB, clinically relevant non-major bleeding; CTEPH, chronic thromboembolic pulmonary hypertension; DVT, deep vein thrombosis; GP, general practitioner; ICH, intracranial haemorrhage; LMWH, low-molecular weight heparin; MB, major bleeding; PE, pulmonary embolism; PTS, post-thrombotic syndrome; VTE, venous thromboembolism

[a] Assumed to be equal to the costs of non-fatal PE

For peer review only

$_b$ *Based on an average weight between 69 and 82 kg.*

$_c$ *Based on DRG code 070419 and only taken into account for rivaroxaban treated patients*

References

1. Heisen M, Treur MJ, Heemstra HE, Giesen EBW, Postma MJ. Cost-effectiveness analysis of rivaroxaban for treatment and secondary prevention of venous thromboembolism in the Netherlands. J Med Econ. 2017 Aug 3;20(8):813–24.
2. Baeten S a, van Exel NJ a, Dirks M, Koopmanschap M a, Dippel DW, Niessen LW. Lifetime health effects and medical costs of integrated stroke services - a non-randomized controlled cluster-trial based life table approach. Cost Eff Resour Alloc. 2010;8(1):21.
3. Medicijnkosten.nl. The National Health Care Institute (ZIN) [Internet].
4. Young AM, Marshall A, Thirlwall J, Chapman O, Lokare A, Hill C, et al. Comparison of an Oral Factor Xa Inhibitor With Low Molecular Weight Heparin in Patients With Cancer With Venous Thromboembolism: Results of a Randomized Trial (SELECT-D). J Clin Oncol. 2018;36(20):2017–23.
5. Hakkaart-van Roijen L, van der Linden N, Bouwmans C, Kanters T, Swan Tan S. Kostenhandleiding: Methodologie van kostenonderzoek en referentieprijzen voor economische evaluaties in de gezondheidszorg. Zorginstituut Ned. 2016;1–120.
6. van Bellen B, Bamber L, Correa de Carvalho F, Prins M, Wang M, Lensing AWA. Reduction in the length of stay with rivaroxaban as a single-drug regimen for the treatment of deep vein thrombosis and pulmonary embolism. Curr Med Res Opin. 2014;30(5):829–37.
7. Dutch Health Authorities. NZa zorgproductapplicatie. Declaration code: 070419. [Internet]. 2018.
8. Roijen LH, Linden N van der, Bouwmans C, Kanters T, Tan SS. Dutch manual for costing studies in health care. Diemen; 2015.
9. de Klerk M, de Boer A, Plaisier I, Schyns P, Kooiker S. Informele hulp: wie doet er wat? - Rapport - SCP [Internet]. 2015-35. 2015.
10. van den Berg B, Brouwer W, van Exel J, Koopmanschap M, van den Bos GAM, Rutten F. Economic valuation of informal care: lessons from the application of the opportunity costs and proxy good methods. Soc Sci Med. 2006;62(4):835–45.

For peer review only

Supplementary data file – Table S3

Manuscript title: Cost-effectiveness analysis and budget impact of rivaroxaban in cancer patients at risk of recurrent venous thromboembolism

Table S3. Utility values included in the cost-effectiveness model

	Value (95% CI)	Distribution	Reference
**Utilities**			
Index VTE			
0–1 month	0.565 (0.501 – 0.620)	Beta	[1]
1–2 months	0.655 (0.585 – 0.713)	Beta	[1]
2–3 months	0.674 (0.606 – 0.729)	Beta	[1]
3–4 months	0.698 (0.635 – 0.750)	Beta	[1]
4–5 months	0.707 (0.645 – 0.758)	Beta	[1]
Baseline utility 6 months after index VTE	0.715 (0.646 – 0.770)	Beta	[1]
Recurrent VTE			
DVT	0.605 (0.514 – 0.678)	Beta	[1]
Non-fatal symptomatic PE	0.621 (0.477 – 0.725)	Beta	[1]
Non-fatal incidental PE	0.664 (0.615 – 0.707)	Beta	[1]
Non-ICH MB	0.593 (0.461 – 0.693)	Beta	[1]
CRNMB	0.622 (0.568 – 0.669)	Beta	[1]
**Utility decrements**			
Recurrent VTE within first six months after index VTE			
DVT	0.040 (0.000 – 0.158)	Beta	[1]
Symptomatic PE	0.024 (0.000 – 0.195)	Beta	[1]
Incidental PE	0.189 (0.021 – 0.404)	Beta	[1]
ICH	0.380 (0.285 – 0.475)	Beta	[2]
Severe PTS (<6 months after diagnosis)	0.186 (0.090 – 0.280)	Beta	[1]
Severe PTS (>6 months after diagnosis)	0.070 (0.053 – 0.088)	Beta	[2]
CTEPH			
0-1 year	0.194 (0.071 – 0.303)	Beta	[3]
1–4 years	0.109 (0.000 – 0.244)	Beta	[3]
4–5 years	0.079 (0.000 – 0.277)	Beta	[3]

Abbreviations: CI, confidence interval; CRNMB, clinically relevant non-major bleeding; CTEPH, chronic thromboembolic pulmonary hypertension; DVT, deep vein thrombosis; ICH, intracranial haemorrhage; MB, major bleeding; PE, pulmonary embolism; PTS, post-thrombotic syndrome; VTE, venous thromboembolism

References

1. Lloyd AJ, Dewilde S, Noble S, Reimer E, Lee AYY. What Impact Does Venous Thromboembolism and Bleeding Have on Cancer Patients' Quality of Life? Value Heal. 2018;21(4):449–55.
2. Stevanović J, de Jong LA, Kappelhoff BS, Dvortsin EP, Voorhaar M, Postma MJ. Dabigatran for the Treatment and Secondary Prevention of Venous Thromboembolism; A Cost-Effectiveness Analysis for the Netherlands. PLoS One. 2016;11(10):e0163550.
3. Roman A, Barbera JA, Castillo MJ, Muñoz R, Escribano P. Health-related quality of life in a national cohort of patients with pulmonary arterial hypertension or chronic thromboembolic pulmonary hypertension. Arch Bronconeumol. 2013;49(5):181–8.

For peer review only

Supplementary data file – Figure S1

Manuscript title: Cost-effectiveness analysis and budget impact of rivaroxaban in cancer patients at risk of recurrent venous thromboembolism

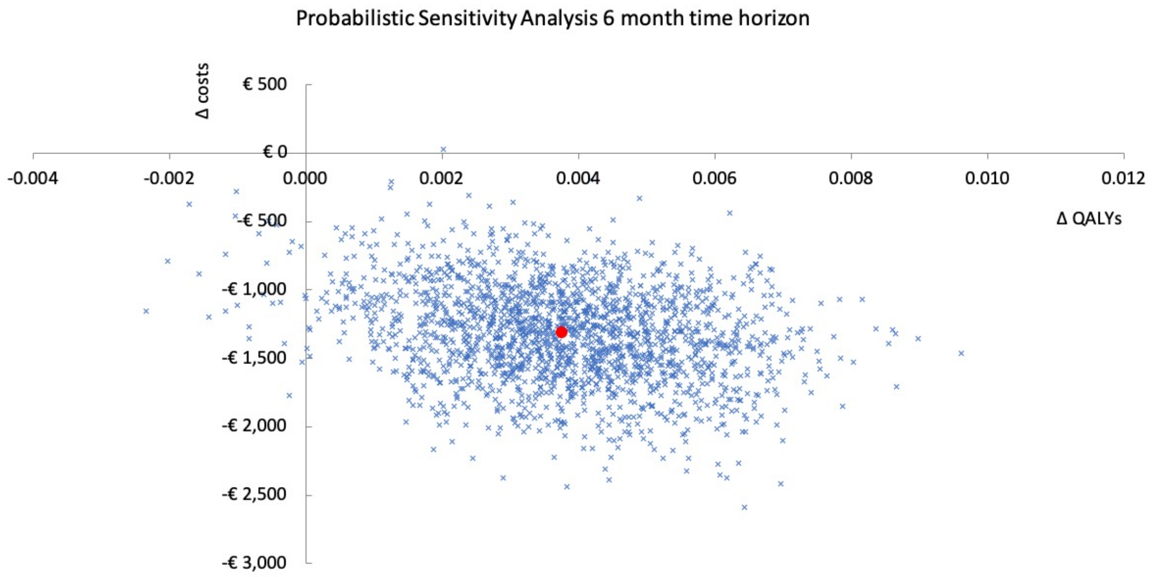

Figure S1. Probabilistic sensitivity analysis with six-month time horizon (scenario 1). *The red mark represents the deterministic incremental cost-effectiveness ratio. Abbreviation: QALY, quality adjusted life-year*

For peer review only

Supplementary data file – Figure S2

Manuscript title: Cost-effectiveness analysis and budget impact of rivaroxaban in cancer patients at risk of recurrent venous thromboembolism

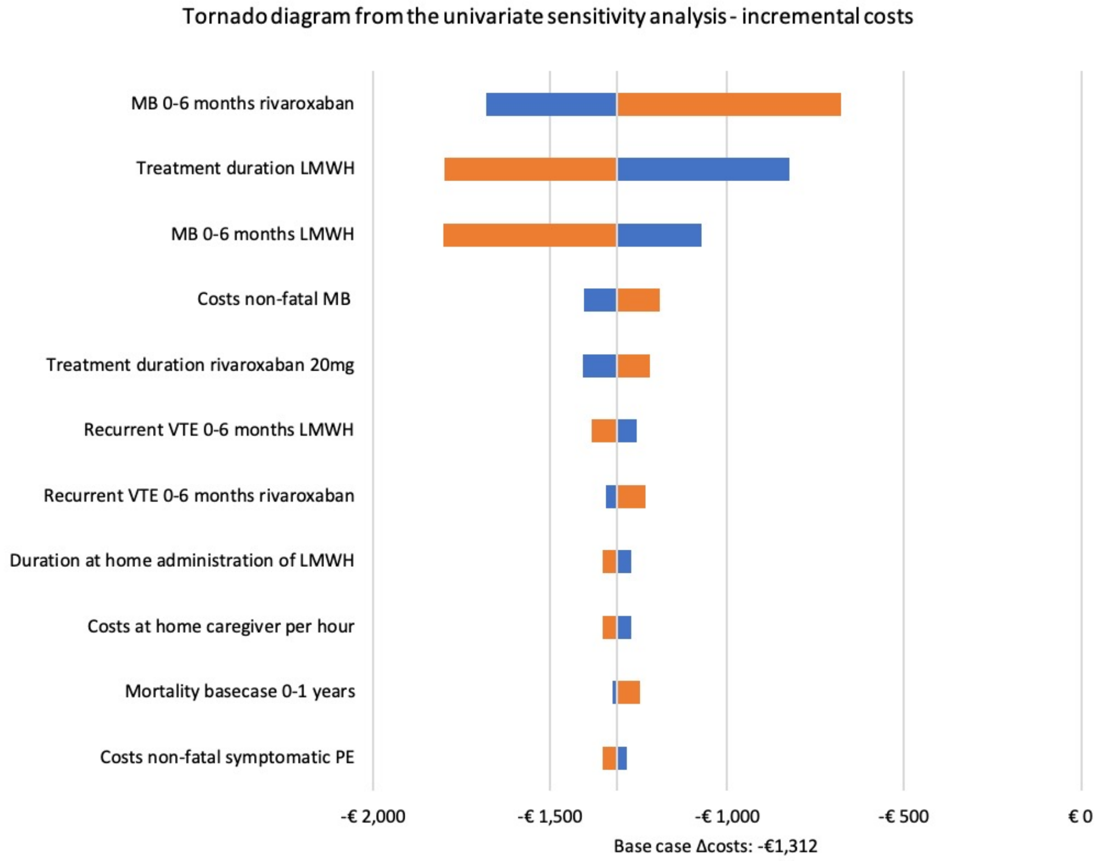

Figure S2. Tornado diagram from the univariate sensitivity analysis for scenario 1 showing the impact of parameters on the incremental costs. *Abbreviations: MB, major bleeding; PE, pulmonary embolism; VTE, venous thromboembolism*

only

Supplementary data file – Figure S3

Manuscript title: Cost-effectiveness analysis and budget impact of rivaroxaban in cancer patients at risk of recurrent venous thromboembolism

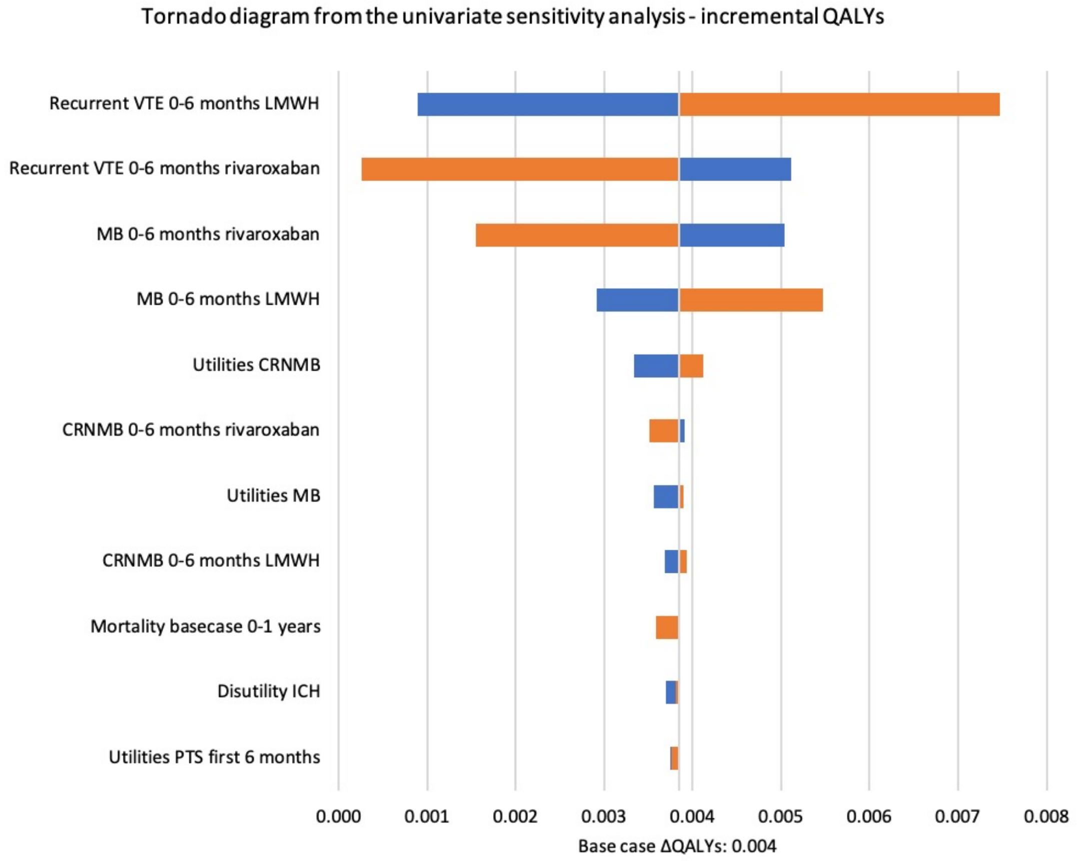

Figure S3. Tornado diagram from the univariate sensitivity analysis for scenario 1 showing the impact of parameters on the incremental QALYs. *Abbreviations: CRNMB, clinically relevant non-major bleeding; ICH, intracranial haemorrhage; MB, major bleeding; PE, pulmonary embolism; VTE, venous thromboembolism*

For peer review only - http://bmjopen.bmj.com/site/about/guidelines.xhtml

CHEERS checklist—Items to include when reporting economic evaluations of health interventions

Section/item	Item No	Recommendation	Reported on page No/ line No
**Title and abstract**			
Title	1	Identify the study as an economic evaluation or use more specific terms such as "cost-effectiveness analysis", and describe the interventions compared.	Page 1, line 9-10
Abstract	2	Provide a structured summary of objectives, perspective, setting, methods (including study design and inputs), results (including base case and uncertainty analyses), and conclusions.	Page 1, line 34-64
**Introduction**			
Background and objectives	3	Provide an explicit statement of the broader context for the study.	Page 4, line 91-120
		Present the study question and its relevance for health policy or practice decisions.	Page 4, line 121-126
**Methods**			
Target population and subgroups	4	Describe characteristics of the base case population and subgroups analysed, including why they were chosen.	Page 5, line 149-151<br>Page 5, line 179
Setting and location	5	State relevant aspects of the system(s) in which the decision(s) need(s) to be made.	Page 4, line 130-140
Study perspective	6	Describe the perspective of the study and relate this to the costs being evaluated.	Page 5, line 135-137
Comparators	7	Describe the interventions or strategies being compared and state why they were chosen.	Page 4, line 130-132
Time horizon	8	State the time horizon(s) over which costs and consequences are being evaluated and say why appropriate.	Page 5, line 152-154
Discount rate	9	Report the choice of discount rate(s) used for costs and outcomes and say why appropriate.	Page 8, line 241<br>Page 9, line 264
Choice of health outcomes	10	Describe what outcomes were used as the measure(s) of benefit in the evaluation and their relevance for the type of analysis performed.	Page 4, line 133-140
Measurement of effectiveness	11a	*Single study-based estimates:* Describe fully the design features of the single effectiveness study and why the single study was a sufficient source of clinical effectiveness data.	
	11b	*Synthesis-based estimates*: Describe fully the methods used for identification of included studies and synthesis of clinical effectiveness data.	Page 6, line 184-210
Measurement and valuation of preference based outcomes	12	If applicable, describe the population and methods used to elicit preferences for outcomes.	
Estimating resources and costs	13a	*Single study-based economic evaluation:* Describe approaches used to estimate resource use associated with the alternative interventions. Describe primary or secondary research methods for valuing each resource item in terms of its unit cost. Describe any adjustments made to approximate to opportunity costs.	
	13b	*Model-based economic evaluation:* Describe approaches and data sources used to estimate	Page 8, line 213-253

For peer review only

Section/item	Item No	Recommendation	Reported on page No/ line No
		resource use associated with model health states. Describe primary or secondary research methods for valuing each resource item in terms of its unit cost. Describe any adjustments made to approximate to opportunity costs.	
Currency, price date, and conversion	14	Report the dates of the estimated resource quantities and unit costs. Describe methods for adjusting estimated unit costs to the year of reported costs if necessary. Describe methods for converting costs into a common currency base and the exchange rate.	Page 8, line 246
Choice of model	15	Describe and give reasons for the specific type of decision-analytical model used. Providing a figure to show model structure is strongly recommended.	Page 5, line 143-176
Assumptions	16	Describe all structural or other assumptions underpinning the decision-analytical model.	Page 5, line 162-166<br>Page 8, line 230-240
Analytical methods	17	Describe all analytical methods supporting the evaluation. This could include methods for dealing with skewed, missing, or censored data; extrapolation methods; methods for pooling data; approaches to validate or make adjustments (such as half cycle corrections) to a model; and methods for handling population heterogeneity and uncertainty.	Page 7, line 190-201<br>Page 10, line 284-301
**Results**			
Study parameters	18	Report the values, ranges, references, and, if used, probability distributions for all parameters. Report reasons or sources for distributions used to represent uncertainty where appropriate. Providing a table to show the input values is strongly recommended.	Page 6, line 201-210<br>Page 8, line 241-253<br>Page 9, line 264-271<br>Page 10, line 276-278
Incremental costs and outcomes	19	For each intervention, report mean values for the main categories of estimated costs and outcomes of interest, as well as mean differences between the comparator groups. If applicable, report incremental cost-effectiveness ratios.	Page 11, line 320-323<br>Page 14, line 393-398
Characterising uncertainty	20a	*Single study-based economic evaluation:* Describe the effects of sampling uncertainty for the estimated incremental cost and incremental effectiveness parameters, together with the impact of methodological assumptions (such as discount rate, study perspective).	
	20b	*Model-based economic evaluation:* Describe the effects on the results of uncertainty for all input parameters, and uncertainty related to the structure of the model and assumptions.	Page 13, line 354-389
Characterising heterogeneity	21	If applicable, report differences in costs, outcomes, or cost-effectiveness that can be explained by variations between subgroups of patients with different baseline characteristics or other observed variability in effects that are not reducible by more information.	Page 12, line 311-333
**Discussion**			
Study findings, limitations, generalisability, and current knowledge	22	Summarise key study findings and describe how they support the conclusions reached. Discuss limitations and the generalisability of the findings and how the findings fit with current knowledge.	Page 11, line 323-351
**Other**			

For peer review only

Section/item	Item No	Recommendation	Reported on page No/ line No
Source of funding	23	Describe how the study was funded and the role of the funder in the identification, design, conduct, and reporting of the analysis. Describe other non-monetary sources of support.	Page 3, line 79
Conflicts of interest	24	Describe any potential for conflict of interest of study contributors in accordance with journal policy. In the absence of a journal policy, we recommend authors comply with International Committee of Medical Journal Editors recommendations.	Page 3, line 81-84

For consistency, the CHEERS statement checklist format is based on the format of the CONSORT statement checklist

For peer review only

BMJ Open

Cost-effectiveness analysis and budget impact of rivaroxaban compared with dalteparin in cancer patients at risk of recurrent venous thromboembolism

Journal:	*BMJ Open*
Manuscript ID	bmjopen-2020-039057.R2
Article Type:	Original research
Date Submitted by the Author:	13-Oct-2020
Complete List of Authors:	de Jong, Lisa; University of Groningen, Unit of Pharmacotherapy, -Epidemiology and -Economics<br>van der Velden, Annette; Martini Hospital, Department of Internal Medicine<br>Hulst, Marinus; Martini Hospital, Department of Clinical Pharmacy and Toxicology; University Medical Centre Groningen, Department of Health Sciences<br>Postma, Maarten; University Medical Centre Groningen, Department of Health Sciences; University of Groningen, Department of Economics, Econometrics & Finance
**Primary Subject Heading**:	Health economics
Secondary Subject Heading:	Cardiovascular medicine, Health economics
Keywords:	Anticoagulation < HAEMATOLOGY, ONCOLOGY, Thromboembolism < CARDIOLOGY, HEALTH ECONOMICS

SCHOLARONE™
Manuscripts

I, the Submitting Author has the right to grant and does grant on behalf of all authors of the Work (as defined in the below author licence), an exclusive licence and/or a non-exclusive licence for contributions from authors who are: i) UK Crown employees; ii) where BMJ has agreed a CC-BY licence shall apply, and/or iii) in accordance with the terms applicable for US Federal Government officers or employees acting as part of their official duties; on a worldwide, perpetual, irrevocable, royalty-free basis to BMJ Publishing Group Ltd ("BMJ") its licensees and where the relevant Journal is co-owned by BMJ to the co-owners of the Journal, to publish the Work in this journal and any other BMJ products and to exploit all rights, as set out in our licence.

The Submitting Author accepts and understands that any supply made under these terms is made by BMJ to the Submitting Author unless you are acting as an employee on behalf of your employer or a postgraduate student of an affiliated institution which is paying any applicable article publishing charge ("APC") for Open Access articles. Where the Submitting Author wishes to make the Work available on an Open Access basis (and intends to pay the relevant APC), the terms of reuse of such Open Access shall be governed by a Creative Commons licence – details of these licences and which Creative Commons licence will apply to this Work are set out in our licence referred to above.

Other than as permitted in any relevant BMJ Author's Self Archiving Policies, I confirm this Work has not been accepted for publication elsewhere, is not being considered for publication elsewhere and does not duplicate material already published. I confirm all authors consent to publication of this Work and authorise the granting of this licence.

For peer review only

Cost-effectiveness analysis and budget impact of rivaroxaban compared with dalteparin in cancer patients at risk of recurrent venous thromboembolism

Short title: Economic evaluation of rivaroxaban in cancer patients

Authors:

Lisa A. de Jong[1], Annette W.G. van der Velden[2], Marinus van Hulst[3,4]*, Maarten J. Postma[4,5]*

Affiliations:

[1] Unit of PharmacoTherapy, -Epidemiology & -Economics, University of Groningen, Groningen Research Institute of Pharmacy (GRIP), Antonius Deusinglaan 1, Groningen, the Netherlands

[2] Department of Internal Medicine, Martini Hospital, Van Swietenplein 1, Groningen, The Netherlands

[3] Department of Clinical Pharmacy and Toxicology, Martini Hospital, Van Swietenplein 1, Groningen, The Netherlands

[4] Department of Health Sciences, University of Groningen, University Medical Center Groningen, Hanzeplein 1, Groningen, The Netherlands

[5] Department of Economics, Econometrics & Finance, University of Groningen, Faculty of Economics & Business, Nettelbosje 2, Groningen, The Netherlands

*Shared last author

Corresponding author:

Lisa A de Jong

E-mail: l.a.de.jong@rug.nl

Phone: +31630048956

ORCID ID: 0000-0001-8814-0670

Author contributions:

LA de Jong contributed to the design, interpretation of the data, modelling, drafting the manuscript and revisions. M van Hulst, MJ Postma and AWG van der Velden contributed to the design, interpretation of the data, validation of the model and drafting the manuscript.

For peer review only - http://bmjopen.bmj.com/site/about/guidelines.xhtml

Abstract

Objectives: In the SELECT-D trial, rivaroxaban showed relatively low venous thromboembolism (VTE) recurrence but higher bleeding compared with dalteparin in cancer patients. We aim to calculate the cost-effectiveness and budget impact of rivaroxaban compared with dalteparin in cancer patients at risk of recurrent VTE.

Setting: We built a Markov model to calculate the cost-effectiveness from a societal perspective over a five-year time horizon for the Dutch healthcare setting.

Participants: A hypothetical cohort of 1,000 cancer patients with VTE entered the model with baseline characteristics based on the SELECT-D trial.

Intervention: Six months treatment with rivaroxaban (15 mg twice daily for first three weeks followed by 20 mg once daily) was compared with six months treatment with dalteparin (200 IU/kg daily during month one followed by 150 IU/kg daily).

Primary and secondary outcome measures: The primary outcome of the cost-effectiveness analysis was the incremental cost-effectiveness ratio (ICER). The robustness of the model was evaluated in probabilistic and univariate sensitivity analyses. A budget impact analysis was performed to calculate the total annual financial consequences for a societal perspective in the Netherlands.

Results: In the base case and all scenarios, rivaroxaban were cost-saving while also slightly improving the patient's health, resulting in economically dominant ICERs. In the probabilistic sensitivity analysis, 77.8% and 98.7% of the simulations showed rivaroxaban to be cost-saving and more effective for a five-year and six-month time horizon, respectively. Rivaroxaban can save up to €11,326,763 (confidence interval: €5,164,254–€17,363,231) in approximately 8,000 cancer patients with VTE per year compared with dalteparin based on a one-year time horizon.

Conclusions: Treatment with rivaroxaban is economically dominant over dalteparin in cancer patients at risk for recurrent VTE in the Netherlands. The use of rivaroxaban instead of dalteparin can save over ten million euros per year, primarily driven by the difference in drug costs.

Strengths and limitations of this study

- This analysis used sophisticated pharmacoeconomic modelling methods to conduct cost-effectiveness and budget impact analyses, presenting the economic impact on a patient as well as on a population level.
- Our model is based on timely, robust data from the important SELECT-D trial.
- Various additional scenarios were used to analyse the effect of different assumptions and clinical situations.
- We assumed a six-month treatment duration for all patients, while in clinical practice the treatment duration may vary between patients.
- Due to lack of data, the productivity losses were not taken into account.

Funding statement: This work was supported by Bayer Pharma Netherlands. The sponsor was involved with the start of the project, but they were not involved in the identification of data, design, conduct, and reporting of the analysis. Award/grant number: not applicable.

Competing interests: LA De Jong, M van Hulst and AWG van der Velden declare that they have no competing interest with relation to subject. Postma MJ has received research grants from various pharmaceutical companies, including but not limiting to Bayer, Pfizer, Bristol-Myers Squibb, GSK, Roche and Novartis.

For peer review only - http://bmjopen.bmj.com/site/about/guidelines.xhtml

Introduction

Venous thromboembolism (VTE), comprising both pulmonary embolism (PE) and deep vein thrombosis (DVT), is a major challenge in patients with cancer [1]. In addition to the characteristics of the cancer itself, cancer therapy (chemotherapy and cancer surgery) has effects on the patient's coagulation system and therefore increases the risk of VTE and bleeding [2,3]. VTE in cancer patients can cause unnecessary hospitalizations, interruption or postponement of cancer treatment, and increased mortality, leading to decreased quality of life and increased costs.

VTE is treated with anticoagulation therapy, and this is continued as prophylaxis for recurrence over a longer period because of the high risk of recurrence during the first months after the initial VTE [4]. Vitamin K antagonists (VKAs) or direct oral anticoagulants (DOACs) are indicated for the treatment and prevention of VTE in the general population [5]. DOACs, are a relatively new class of anticoagulants. Apixaban, dabigatran, edoxaban and rivaroxaban are the four DOACs that are currently registered for the prevention of recurrent VTE in Europe. DOACs have a more beneficial efficacy/safety ratio, do not require routine measurements of the INR, and show fewer food-drug and drug-drug interactions compared with VKAs [6,7].

The guidelines recommend against the use of VKAs in cancer patients because of potential drug interactions, liver dysfunction, and malnutrition, all of which lead to fluctuations of the international normalized ratio (INR) and could result in negative patient outcomes [8–11]. Moreover, trials in cancer patients with VTE have shown that LMWH is more effective in the prevention of recurrent VTE compared with VKA, without increasing bleeding risk [12–14]. Therefore, the guidelines recommend at least 6 months of therapeutic treatment with a daily subcutaneous injection of low molecular weight heparin (LMWH, e.g., dalteparin) in cancer patients [8–11]. However, recently, DOACs rivaroxaban and edoxaban were also added as treatment options for the prevention of recurrent VTE in cancer patients. This recommendation was based on the results from the SELECT-D and HOKUSAI VTE Cancer trials [15,16].

The SELECT-D is a multicenter, randomized, clinical pilot trial in the UK; it is a head-to-head comparison of rivaroxaban and dalteparin in 406 patients with active cancer who had experienced a symptomatic PE, incidental PE, or symptomatic DVT [15]. Incidental PEs are non-symptomatic PEs that are incidentally found during tumour imaging. The trial researchers found that rivaroxaban reduces the recurrence of VTE (six month cumulative VTE recurrence rate: 4% versus 11%) at the cost of an increased risk of bleeding (six month cumulative major bleeding [MB] rate: 6% versus 4%; six month cumulative clinically relevant non-major bleeding [CRNMB] rate: 13% versus 4%) compared with dalteparin. These results were comparable to those of a large retrospective study by Streiff et al. [17].

Based on the results of these studies and the fact that DOACs can be orally administered (unlike the subcutaneously injected LMWHs), a greater utilisation of DOACs for VTE in cancer patients might be expected. Since the introduction of DOACs there has been an ongoing discussion about the economic impact of these drugs. To help guide this discussion and inform decision making in this area, we designed and developed an economic model based on the SELECT-D trial to evaluate the cost-effectiveness and budget impact of rivaroxaban compared with dalteparin in cancer patients at risk of recurrent VTE in the Netherlands.

For peer review only - http://bmjopen.bmj.com/site/about/guidelines.xhtml

Methods

The economic model comparing rivaroxaban with dalteparin was designed based on the SELECT-D trial [15], since this study presented the most comprehensive results reflecting recurrent VTE and bleeding complications per event type (symptomatic PE, incidental PE, and DVT) or severity (MB and CRNMB). The primary outcome of the cost-effectiveness analysis is the incremental cost-effectiveness ratio (ICER); this is calculated by dividing the incremental costs by the incremental health effects, expressed in quality adjusted life-years (QALYs). In accordance with Dutch costing guidelines for economic evaluations in healthcare, the ICER was calculated from a societal perspective, which incorporates direct as well as indirect costs both inside and outside the healthcare sector[18]. We performed sensitivity and scenario analyses to test the robustness of the model. Additionally, we conducted a budget impact analysis to reflect the annual financial consequences of the use of rivaroxaban in cancer patients at risk of recurrent VTE in the Netherlands. The analysis was carried out early 2019. The analyses were conducted based on publicly available information which is presented and referenced in the article and Supporting Information files, and did therefore not require any patient consent forms or approval from an ethical review board.

Model outline

We developed a decision-tree-based Markov model using Microsoft Excel 2016 to calculate the ICER. Figure 1 shows a schematic representation of the model, with the disease course being represented by separate health states. A hypothetical cohort of 1,000 cancer patients with VTE entered the model with incidental PE, symptomatic PE, or DVT, represented by the 'index VTE' health state. According to the guidelines, patients with incidental PE should be treated identically to those with symptomatic PE [8,10]. Patient characteristics were based on the SELECT-D trial protocol (Table 1) [15]. The SELECT-D population is representative for the Dutch cancer population, based on age, tumour type, and gender distribution [19]. Patients move through various health states in the model during the follow-up time of five years. Five years was used because overall survival was assumed to be low after five years since the majority (58%) of the SELECT-D trial population had metastatic cancer [15]. We included the following health states in our model (see legend of Figure 1 for abbreviations): 'recurrent incidental PE', 'recurrent symptomatic PE', 'fatal recurrent VTE', 'recurrent DVT', 'ICH', 'non-ICH MB', 'fatal MB', 'CRNMB', 'death by any cause', and 'no event'. Patients were assumed to remain in these states for one cycle, after which they moved back to the 'index VTE' state or the chronic, debilitating 'post-ICH' state, in which they remained until death without being at risk for any further complications. The cycle length was one month. Markov tunnel states (one-month post-VTE, two months post-VTE, …, 60 months post VTE) were used to implement time-dependency. These temporary states can only be visited once, which allows time-dependent future transitions, costs, and health-related quality of life dependent on how long the patient has gone without a recurrent VTE event [20]. The chronic complications post-thrombotic syndrome (PTS) and chronic thromboembolic pulmonary hypertension (CTEPH) were modelled in the background. This means that PTS or CTEPH could occur at any time in the model, regardless of the health state the patient is in. Costs and health effects of these events were taken into account. However, only the severe cases of PTS were modelled, since the costs of minor PTS are considered negligible. For these chronic complications we also used tunnel states since the risks of PTS and CTEPH were also time-dependent.

For peer review only - http://bmjopen.bmj.com/site/about/guidelines.xhtml

Figure 1. Model outline. All patients enter the model in the 'Index VTE' state and move to other states upon the occurrence of one of the following events: recurrent incidental PE, recurrent symptomatic PE, fatal recurrent VTE, recurrent DVT, ICH, non-ICH MB, fatal MB, CRNMB, or death by any cause. The triangles represent the health state a patient will enter after an event. The blue squares are permanent states, in which a patient will remain until death while not being at risk for other events. The red squares represent a transient state: the patient will re-enter the model in the 'Index VTE' state.
Abbreviations: CRNMB, clinically relevant non-major bleeding; DVT, deep vein thrombosis; ICH, intracranial haemorrhage; MB, major bleeding; PE, pulmonary embolism; VTE, venous thromboembolism

Table 1. Patient characteristics of the hypothetical cohort of 1,000 cancer patients at risk of recurrent VTE.

Unit	Value	Reference
Age (years)	67	[15]
Proportion male	53%	[15]
BMI (kg/m^2)	25.6	[15]
Type of cancer		
Early or locally advanced cancer	39%	[15]
Metastatic cancer	58%	[15]
Haematologic malignancy	2%	[15]
Distribution of PE and DVT		
% index VTE that is symptomatic PE	20%	[15]
% index VTE that is incidental PE	53%	[15]
% index VTE that is DVT	27%	[15]

Abbreviations: BMI, body mass index; DVT, deep vein thrombosis; PE, pulmonary embolism; VTE, venous thromboembolism

Transition probabilities

Transition probabilities were used to calculate the number of patients in each health state per one-month cycle. Table S1 summarizes all event rates presented in six-month risks. The event rates were translated into monthly transition probabilities with the following formula:

$$P = 1 - \exp\{-rt\}$$

Where P is the transition probability, r is the event rate, and t is the cycle length (one month) [20].

Event rates of recurrent VTE, MB, and CRNMB in the first six months of treatment were based on the SELECT-D trial [15]. If patients did not experience a recurrent event during this period, anticoagulation treatment was discontinued. Recurrent VTE rates after treatment discontinuation were based on a retrospective study in active-cancer patients experiencing a VTE [4]. Upon the occurrence of a non-fatal recurrent VTE, patients were assigned to another six months treatment, with corresponding event rates. Bleeding risks after treatment discontinuation were based on the outcomes of the cancer population of the HOKUSAI VTE Cancer trial (which followed patients after edoxaban discontinuation for an additional six months) because these data are not reported for the SELECT-D trial [16]. The HOKUSAI VTE Cancer trial was also used to determine the distribution of ICH, non-ICH, and fatal bleeding. The distributions among the different types of VTE (incidental PE, symptomatic PE, DVT, and fatal PE) and MB (ICH, non-ICH, fatal MB) were calculated based on the total number of events in both arms (rivaroxaban and dalteparin) together and assumed it to be treatment-independent, since the

For peer review only - http://bmjopen.bmj.com/site/about/guidelines.xhtml

total number of events in the trials was low. The distributions of the types of VTE event were based on the number of recurrent VTE events observed in the SELECT-D trial in the lower extremities and pulmonary embolisms— other locations of VTE events (brachial, subclavian, jugular, renal plus inferior vena cava, or the extrahepatic vein) were excluded [15]. Mortality rates (death by any cause) were based on Dutch cancer mortality data from the Netherlands Cancer Registry [21]. In the sensitivity analysis, all transition probabilities were varied over beta distributions. For percentages of the type of recurrent VTE and MB, a Dirichlet distribution was used in the sensitivity analysis. As recommended by the Dutch guidelines for economic evaluation of healthcare, the distributions were based on Briggs et al., who described the use of distributions around model input parameters (e.g., distributions limited to positive values (costs) or even confined between 0-1 (probabilities)) [18,20].

Costs

All cost parameters are standardised to 2019 Euros, and summarised in Table S2. Event-related healthcare costs were based on a previous Dutch cost-effectiveness study for rivaroxaban in the general VTE population [22]. Costs of fatal recurrent VTE were assumed to be similar to those of non-fatal symptomatic PE. We assumed no event-related healthcare costs for patients with incidental PE, because these embolisms were found incidentally and did therefore not require physician visits. However, since patients with incidental PE should be treated identically to those with symptomatic PE, we did take medication costs into account. Costs for ICH and CTEPH consisted of acute care costs during the first month after diagnosis, followed by long-term care costs until the patient moved to the 'death' state. Costs of a fatal MB were assumed to be equal to those of non-fatal non-ICH MB.

Drug costs were retrieved from the national medication costs database [23]. For rivaroxaban these costs were based on 15 mg twice daily for three weeks followed by 20 mg once daily. Drug costs of dalteparin were based on 200 IU/kg daily during month one followed by 150 IU/kg daily in months two to six [15,24]. Based on an average body mass index of 25.6 from the SELECT-D trial and an average height of 1.72 m for the Dutch population, we calculated that the average weight was between 69 and 82 kg, which corresponds with a dose of 15,000 IU daily during month one followed by 12,500 IU daily in months two to six [15,25]. Rivaroxaban users were assumed to require an annual check-up of their renal function [6]. We included one-time costs for an injection instruction by a home-caregiver. Administration costs were only accounted to patients with early or locally advanced cancer (39%), since patients with metastatic cancer or haematologic malignancies often already have home care or an informal caregiver who can administer the dalteparin injection. Similarly, informal care costs were only taken into account for this same subgroup.

Based on a previously published report on informal care in the Netherlands, we made a distinction between intensive (26 hours per week) and non-intensive (8 hours per week) informal care [26]. This was multiplied by the average duration and tariff for informal care, obtained from the Dutch cost manual [27]. To prevent double counting, we did not include informal care costs for the chronic complications. Travel costs were taken into account for renal monitoring visits and upon the occurrence of a DVT or CRNMB. Costs related to forgone leisure activity were not taken into account since there are no data available on the impact of a VTE or bleeding on leisure losses in cancer patients. Moreover, the starting age of the population in the model was 67 years (which is the Dutch retirement age) based on the average age of the SELECT-D trial and the fact that the majority (58%) of the patients in the SELECT-D trial had metastatic cancer may indicate a low employment rate.

For peer review only - http://bmjopen.bmj.com/site/about/guidelines.xhtml

Costs were discounted at an annual rate of 4% [18]. In the sensitivity analysis, the costs were varied with gamma distributions corresponding to the 95% confidence interval (CI) [18,20], as indicated in Table S2.

Utilities

Utility scores, used to calculate the QALYs, were derived from a sub-analysis from the CATCH study assessing the EQ-5D scores associated with VTE and recurrent VTE in cancer patients (Table S3) [28]. The CATCH study assessed the effectiveness of six months of treatment with tinzaparin versus warfarin for the treatment of acute venous thromboembolism in patients with active cancer. It was chosen because it aligns well with our population and events of interest. Utility decrements for CTEPH were based on a study assessing EQ-5D VAS scores in CTEPH patients up to 5 years after their initial diagnosis [29]. Utility decrements for ICH and long-term PTS (>6 months after diagnosis) were obtained from a previous cost-effectiveness study [30]. QALYs related to fatal events, such as death due to any cause, fatal PE and fatal MB, were assumed to be 0. QALYs were discounted at 1.5% per annum according to Dutch guidelines [18]. In the sensitivity analyses, utility scores were varied over their 95% CI with a beta distribution [18,20].

Sensitivity analysis

Sensitivity analyses were conducted to check the robustness of the model results to uncertainty and known variations in key input parameters. In the probabilistic sensitivity analysis, all input parameters were varied simultaneously over their 95% CI. If the 95% CI was unavailable and calculating the 95% CI based on the number of events was not possible, the 95% CI was calculated based on a 25% standard error. The ICER was calculated with 2,000 iterations and plotted in a cost-effectiveness plane. A univariate sensitivity analysis was conducted to show the influence of an individual parameter on the ICER. The 10 most influencing parameters were presented in a tornado diagram.

Scenario analysis

We conducted several scenario analyses to show the effect on the outcomes of different (clinical) situations (Table 2).

Table 2. Overview of the scenario analyses.

Scenario	Description	Details
Base case	5-year time horizon from societal perspective	-
1	6-month time horizon from societal perspective	The follow-up period of the SELECT-D trial was six months; therefore, outcomes beyond six months had to be extrapolated based on other publications.
2	Base case analysis from healthcare payer's perspective	In the Netherlands, guidelines advise to calculate the ICER from a societal perspective, while in countries such as the UK or Belgium, the healthcare payer's perspective is preferred. To make results comparable to other countries we also calculated the base case ICER from a healthcare payer's perspective, by excluding the indirect costs.

For peer review only - http://bmjopen.bmj.com/site/about/guidelines.xhtml

3	Base case analysis with dalteparin dose of 12,500 IU	The costs of dalteparin vary with the patient's weight. For the base case analysis, we assumed an average weight between 69 and 82 kg. In scenarios 3 and 4 we calculated the base case ICER with the costs of dalteparin based on weight categories of 57–68 kg (12,500 IE daily during month one followed by 10,000 IE daily in month two to six) and 83–98 kg (18,000 IE daily during month one followed by 15,000 IE daily in month two to six), respectively.
4	Base case analysis with dalteparin dose of 18,000 IU	
5	Scenario 1 with treatment duration based on Streiff et al. [17]	This scenario was similar to scenario 1, except for the treatment period which was based on a study of Streiff et al., who—comparable to SELECT-D—compared rivaroxaban to LMWH for the prevention of recurrent VTE in cancer patients [17]. They found an average treatment duration of one month and three months for LMWH and rivaroxaban, respectively.
6	Base case analysis using drug-specific distributions for the types of VTE and MB	Due to low numbers of VTE and MB events observed in the SELECT-D trial [15] and HOKUSAI VTE Cancer [16] trials, respectively, we calculated the distribution of the types of VTE and MB in the base case analysis based on the total number of events and assumed it to be equal for both drugs. In this scenario we assess the effect of this assumption on the cost-effectiveness results by using the drug-specific distributions of the types of VTE and MB based on the results of the SELECT-D and HOKUSAI VTE Cancer trials [15,16].

Abbreviations: IU, international units; MB, major bleeding; VTE, venous thromboembolism.

For peer review only

Budget impact

A budget impact analysis was conducted to estimate the total annual financial consequences of the implementation of rivaroxaban for the treatment and prevention of VTE in cancer patients within the Dutch healthcare setting. The budget impact was calculated from a societal perspective using the costs calculations from the cost-effectiveness model with a one-year time horizon. We extracted from the model the costs (event-related, treatment, and indirect costs) per patient with a cut-off point of one year for rivaroxaban and dalteparin. The difference in cost per patient was multiplied by the annual number of cancer patients with VTE in the Netherlands. The incidence of VTE in cancer patients and the total number of Dutch cancer patients were used to calculate the yearly number of cancer patients with VTE. The Netherlands Cancer Registry estimated a total of 579,781 cancer patients in 2017 [31]. The incidence of VTE in cancer patients was 13.9 per 1,000 person-years, based on a cohort study of linked UK databases [32]. Based on these numbers we calculated a total of approximately 8,000 cancer patients with VTE per year in the Netherlands. The outcome of the budget impact analysis was presented as the total budget impact per year, including a subdivision of the costs per type (event-related costs, treatment costs and indirect costs) and corresponding 95% CIs derived from PSA.

Results

Cost-effectiveness analysis

Table 3 represents the deterministic results of the base case and scenario analyses. In each scenario, rivaroxaban was economically dominant–meaning that it simultaneously confers better clinical and quality-of-life outcomes at less cost–compared with dalteparin. As such, a numerical ICER is not presented because it has no meaning. Despite the fact that every scenario shows an improvement in the patient's health, the difference in QALYs was very low (incremental QALYs of 0.012 over 5 years' time horizon, which equals 4.4 quality-adjusted life days, in the base case analysis). In the base case analysis, rivaroxaban saved €1,376 per patient compared with dalteparin. The scenario calculating the

For peer review only - http://bmjopen.bmj.com/site/about/guidelines.xhtml

cost-effectiveness over a six-month time horizon resulted in cost-savings of €1,312 per patient (scenario 1). There was increased cost-savings compared with the societal perspective when calculated from a healthcare payer’s perspective (scenario 2). In scenarios 3 and 4 we assessed the effect of variations in the patient’s weight (and thus dalteparin dosing) on the ICER. Compared with the base case analysis, there was decreased cost-savings with a lower dalteparin dose and increased cost-savings with a higher dalteparin dose, both still resulting in dominant ICERs. When comparing three months of rivaroxaban treatment to one month of dalteparin treatment, we found incremental QALYs of 0.016 and cost-savings of €702 per patient (scenario 5). We assessed the effect of using drug-specific distributions of the types of VTE and MB, resulting in cost-savings of €1,815 and incremental QALYs of 0.037 (scenario 6).

The number of events and the corresponding average costs per patient in the base case analysis and scenario 4 (base case analysis with a time horizon of 6 months) are presented in Table 4. Rivaroxaban is associated with a lower number of recurrent VTE events, preventing on average €131 and €108 in costs per patient over five years and over six months, respectively. On the other hand, rivaroxaban causes more bleeding events, especially in the treatment period. ICH and non-ICH MB have the highest incremental event costs per patient. Treatment costs are higher for dalteparin compared with rivaroxaban, with incremental costs of €1,721 and €1,468 in the five-year and the six-month time horizon, respectively. The differences in indirect costs for rivaroxaban compared with dalteparin were €19 and -€2 for the five-year and the six-month time horizon, respectively.

For peer review only

Table 3. Deterministic results per patient of the base case and scenario analyses in a cohort of 1,000 cancer patients (2019, Euros).

	Costs	QALYs	Δ Costs	Δ QALYs	ICER
**Base case analysis** - 5-year time horizon from societal perspective					
Rivaroxaban	€3,139	2.459	-€1,476	0.012	Rivaroxaban dominant
Dalteparin	€4,615	2.448			
**Scenario 1** – 6-month time horizon from societal perspective					
Rivaroxaban	€1,361	0.304	-€1,312	0.004	Rivaroxaban dominant
Dalteparin	€2,673	0.300			
**Scenario 2** – base case analysis from healthcare payer’s perspective					
Rivaroxaban	€2,942	2.459	-€1,496	0.012	Rivaroxaban dominant
Dalteparin	€4,438	2.448			
**Scenario 3** – base case analysis with dalteparin dose of 12,500 IU					
Rivaroxaban	€3,139	2.459	-€1,079	0.012	Rivaroxaban dominant
Dalteparin	€4,218	2.448			
**Scenario 4** – base case analysis with dalteparin dose of 18,000 IU					
Rivaroxaban	€3,139	2.459	-€1,898	0.012	Rivaroxaban dominant
Dalteparin	€5,037	2.448			
**Scenario 5** – scenario 1 with treatment duration based on Streiff et al. [17]					
Rivaroxaban	€1,299	0.289	-€702	0.016	Rivaroxaban dominant
Dalteparin	€2,001	0.273			
**Scenario 6** – base case analysis using drug-specific distributions for the types of VTE and MB					
Rivaroxaban	€3,065	2.463	-€1,815	0.037	Rivaroxaban dominant
Dalteparin	€4,880	2.425			

Abbreviations: ICER, incremental cost-effectiveness ratio; IU, international units; MB, major bleeding; QALY, quality adjusted life-years; VTE, venous thromboembolism.

For peer review only - http://bmjopen.bmj.com/site/about/guidelines.xhtml

Table 4. Number of events and costs per event per patient in a cohort of 1,000 cancer patients (2019, Euros).

**Base case (5-year time horizon)**						
	**Rivaroxaban**		**Dalteparin**		**Incremental**	
	Number of events	Costs per patient	Number of events	Costs per patient	Number of events	Costs per patient
**Event costs**						
Recurrent VTE	191	€311.85	275	€442.92	-84	-€131
Non-fatal symptomatic recurrent PE	33	€168.36	48	€239.13	-15	-€71
Non-fatal incidental recurrent PE	58	-	84	-	-26	
Non-fatal recurrent DVT	83	€59.31	120	€84.23	-37	-€25
Fatal recurrent VTE	17	€84.18	24	€119.56	-7	-€35
ICH	11	€550.70	9	€438.40	2	€112
Non-ICH MB	98	€1,106.87	79	€902.47	19	€204
Fatal MB	5	€51.48	4	€41.98	1	€10
CRNMB	197	€56.28	92	€26.93	105	€29
PTS	61	€92.72	61	€92.37	0	€0
CTEPH	20	€223.79	20	€222.83	0	€1
**Total event costs**		€2,705.54		€2,610.83		€95
**Treatment costs**		€548.83		€2,270.33		-€1,721
**Indirect costs**		€196.31		€177.08		€19
**Scenario 1 (6-month time horizon)**						
	**Rivaroxaban**		**Dalteparin**		**Incremental**	
	Number of events	Costs per patient	Number of events	Costs per patient	Number of events	Costs per patient
**Event costs**						
Recurrent VTE	38	€58.95	109	€166.96	-70	-€108
Non-fatal symptomatic recurrent PE	7	€31.82	19	€90.14	-12	-€58
Non-fatal incidental recurrent PE	12	-	33	-	-21	-
Non-fatal recurrent DVT	17	€11.21	47	€31.75	-31	-€21
Fatal recurrent VTE	3	€15.91	9	€45.07	-6	-€29
ICH	6	€142.82	4	€94.25	2	€49
Non-ICH MB	50	€539.38	33	€355.95	17	€183
Fatal MB	2	€25.09	2	€16.56	1	€9
CRNMB	130	€35.99	38	€10.62	91	€25
PTS	14	€20.59	14	€20.56	0	€0
CTEPH	3	€21.96	3	€21.93	0	€0
**Total event costs**		€903.72		€2,639.25		-€1,736
**Treatment costs**		€479.40		€1,947.45		-€1,468
**Indirect costs**		€36.50		€38.39		-€2

Abbreviations: CRNMB, clinically relevant non-major bleeding; CTEPH, chronic thromboembolic pulmonary hypertension; DVT, deep vein thrombosis; ICH, intracranial haemorrhage; MB, major bleeding; PE, pulmonary embolism; PTS, post thrombotic syndrome; VTE, venous thromboembolism

In the probabilistic sensitivity analysis, we assessed the robustness of the model over a five-year time horizon (base case) and a six-month time horizon (scenario 1). The results are presented in cost-effectiveness planes in Figure 2 and Figure S1. In the base case analysis, rivaroxaban was in the majority (77.8%) of the 2,000 iterations cost-saving and more effective compared with dalteparin. In 22.2% of the iterations rivaroxaban was cost-saving but less effective compared with dalteparin. In scenario 1, rivaroxaban was in almost all (98.7%) the iterations cost-saving and more effective compared with dalteparin.

The influence of the individual input parameters on the base case incremental costs and QALYs are analysed in the univariate sensitivity analysis. The tornado diagrams (Figure 3 and Figure 4) present

For peer review only - http://bmjopen.bmj.com/site/about/guidelines.xhtml

the 10 input parameters with the highest impact in the base case analysis. The risk of MB for both rivaroxaban and dalteparin, treatment duration of dalteparin, and recurrent VTE risks during the first six months after a VTE had the highest influence on the incremental costs. Similarly, the risk of MB and recurrent VTE in the first six months for rivaroxaban and dalteparin showed the highest influence on the incremental QALYs. Similar results were found in the univariate sensitivity analysis of scenario 1 (Figure S2 and Figure S3).

Figure 2. Probabilistic sensitivity analysis of the base case with five-year time horizon (base case analysis). *The red mark represents the deterministic incremental cost-effectiveness ratio. Abbreviation: QALY, quality adjusted life-year*

Figure 3. Tornado diagram from the univariate sensitivity analysis for the base case analysis showing the impact of parameters on the incremental costs. *Abbreviations: ICH, intracranial haemorrhage; MB, major bleeding; PE, pulmonary embolism; VTE, venous thromboembolism*

Figure 4. Tornado diagram from the univariate sensitivity analysis for the base case analysis showing the impact of parameters on the incremental QALYs. *Abbreviations: CRNMB, clinically relevant non-major bleeding; ICH, intracranial haemorrhage; MB, major bleeding; VTE, venous thromboembolism*

Budget impact

The results of the budget impact analysis are presented in Table 5. The replacement of LMWHs (including dalteparin) with rivaroxaban can lead to cost-savings of a maximum of €11,326,763 (€5,164,254–€17,363,231) over approximately 8,000 cancer patients with VTE based on a one-year time horizon. A reduction in treatment costs can lead to savings of up to €12.6 million. Event-related costs and indirect costs slightly increase by €1,234,467 (€-2,103,366–€5,231,955) and €2,101 (€-173,830–€184,677), respectively, when LMWHs are replaced by rivaroxaban.

Table 5. Budget impact (95% CI) over one-year time horizon in the Netherlands.

Event-related costs	€1,234,467 (€-2,103,366–€5,231,955)
Treatment costs	€-12,559,130 (€-17,327,405–€-8,149,498)
Indirect costs	€-2,101 (€-173,830–€184,677)
**Budget impact**	**€-11,326,763 (€-17,363,231–€-5,164,254)**

Abbreviation: CI, confidence interval

Discussion

Thrombosis treatment is a challenge in cancer patients. According to the guidelines, LMWHs and DOACs edoxaban and rivaroxaban are the preferred treatment for the prevention of recurrent VTE in cancer patients [8–11]. We have assessed the cost-effectiveness and budget impact of rivaroxaban in cancer patients at risk of recurrent VTE based on the SELECT-D trial [15]. We conclude that, in the Netherlands, rivaroxaban is a cost-saving treatment option with a small health benefit per patient over

For peer review only - http://bmjopen.bmj.com/site/about/guidelines.xhtml

five years compared with dalteparin. Comprehensive sensitivity analyses confirm that results generated by our model are robust.

The cost-savings associated with rivaroxaban were mainly driven by the difference in treatment costs. It should be noted that this is specifically the case for the Netherlands, and may differ in other countries. The VTE recurrence and MB risks also showed to have a high influence on the incremental costs and QALYs in the univariate sensitivity analysis. The SELECT-D trial showed a relatively low VTE recurrence but higher bleeding (especially CRNMB) compared with dalteparin. This cost-effectiveness model allowed to address the question if the reduction in VTE recurrence outweighs the increase in bleeding events.

A total of 84 VTE-related events were prevented over five years, leading to an average cost-saving of €131 per patient. This is line with findings from a recent study that assessed the VTE-related healthcare costs in cancer patients, which found that rivaroxaban treated patients had a significantly lower total VTE-related costs relative to patients treated with LMWH [33]. Although the cost difference between the rivaroxaban and dalteparin cohorts was even greater with $12,004 per patient per year.

On the other hand, MB events were more frequent with rivaroxaban compared with dalteparin (11 ICH and 98 non-ICH versus 9 ICH and 79 non-ICH, respectively). MB events are very burdensome and frequently severely disabling, leading to high acute and long-term direct and indirect costs. In line with the findings from the SELECT-D trial, CRNMB events were much more frequent with rivaroxaban compared with dalteparin (197 and 92, respectively). Although the difference between rivaroxaban and dalteparin in CRNMB (105 events over 5-year time horizon) is greater than for MB (20 events over 5-year time horizon), the influence on the incremental costs and QALYs were lower because CRNMB is relatively less burdensome.

The indirect costs were higher for rivaroxaban than for dalteparin in the base case scenario. This can be explained by the increased number of MB events with rivaroxaban compared with dalteparin. Moreover, there were no data available on leisure activity losses caused by the occurrence of a VTE event in patients who are already burdened with cancer. Therefore, the indirect costs might have been underestimated, possibly leading to lower cost-savings. The indirect costs account for €196 to €177 per rivaroxaban and dalteparin patient, respectively, over five years—approximately 4-6% of the total cost—however, they do not have a major influence on the differences between the two drugs (€19 and -€2 for the five-year and 6-month time horizon, respectively). This suggests that, although the indirect costs might have been underestimated, rivaroxaban is still likely to be cost-saving compared with dalteparin.

As mentioned, the main driver of the cost-savings is the difference in treatment costs. In the cost-effectiveness analysis, we estimated that more than €1,700 per patient over a five-year period can be saved on treatment costs, compared with dalteparin. Moreover, in the scenario analysis we varied the price of dalteparin based on weight. Although the lowest dose (12,500 IU daily during month one followed by 10,000 IU in months two to six based on weight class 57–68 kg) had a lower price, €8.06 versus €9.93, the ICER remained cost-saving. Rivaroxaban users were assumed to require an annual check-up of their renal function. However, cancer patients (especially those with metastatic cancer) are at higher risk for renal impairment and may be tested much more frequently [34]. This may have caused an overestimation of the costs of rivaroxaban, and therefore reduced the cost-savings estimate of rivaroxaban compared with dalteparin.

In the budget impact analysis, we calculated that rivaroxaban replacing LMWH (including dalteparin) leads to cost-savings of €11,326,763 within one year over a total of 8,000 cancer patients. This is the absolute maximum, since it is not possible to treat each patient with rivaroxaban from a

For peer review only - http://bmjopen.bmj.com/site/about/guidelines.xhtml

clinical perspective. In practice, the market share of rivaroxaban will be lower—despite the fact that there are three other DOACs that could be prescribed—because there are some clinical considerations that should be taken into account. Firstly, although DOACs have far fewer drug interactions than VKAs, it should be noted that rivaroxaban is metabolized by CYP3A4 enzymes [1]. Cancer patients, especially those with haematological cancer, are at high risk for opportunistic and fungal infections, for which they are often treated with CYP3A4 inhibitors or inducers [35]. For this reason, prescription of rivaroxaban for the prevention of recurrent VTE in cancer patients must be done carefully [1]. This interaction does not play a role in LWMH treatment.

Secondly, the balance between the risk of thrombosis and the risk of bleeding should always be a consideration in the prescription of anticoagulants. For example, DOACs are not advised in patients with GI tumours, due to a higher risk of GI bleeding [8–11]. Some prediction scores for primary prevention have been developed to predict thrombosis risk in cancer patients, since thrombosis prophylaxis is most effective in patients with an increased VTE risk. Unfortunately, for cancer these scores have still not been shown to reliably identify patients with the highest risk [36]. Predictive scores for bleeding, such as the HAS-BLED score used for atrial fibrillation patients, are also needed.

A third consideration is the oral administration of rivaroxaban. Although it is less burdensome than the LMWH injections, oral administration can be problematic in patients with anorexia and vomiting, which is often seen as a side effect in cancer therapy [15]. Moreover, low food intake might influence the metabolism of rivaroxaban resulting in lower bioavailability [37]. Lastly, adherence is always a point of discussion, but since adherence to current guidelines is often low [36], we feel that adherence to rivaroxaban might be relatively high than LMWHs due to the more patient-friendly administration.

Our analysis is not without limitations. It should also be noted that 58% of the patients included in the SELECT-D trial had metastatic cancer, and thus results and conclusions pertain mostly to severely ill patients. Also, the majority (53%) of the initial VTE events were incidental PE, related to computed tomography imaging for tumour status [15]. Additionally, as with all cost-effectiveness models some assumptions were required due to lack of data.

We assumed that patients were treated with anticoagulation over six months, which is in line with the guidelines [8–11]. Previous studies have shown that adherence to these guidelines is poor [36]. As seen in the study by Streiff et al [17], in practice, treatment with LMWH is often not six months, presumably due to the fact that LMWH injections are burdensome, there are concerns about the bleeding risk, and the complexity of the treatment of cancer patients [36]. However, this recommended treatment period was also not achieved in many patients treated with rivaroxaban, which resulted in an average duration of three months. We conducted a scenario analysis (scenario 5) to assess this difference in treatment duration (one month of LMWH versus three months of rivaroxaban). These results favoured rivaroxaban, because the incremental QALYs increased while still being cost-saving. On the other hand, there are also some clinical situations in which the treatment period might be longer than six months: for example, in patients with a recurrent VTE event, patients with an active malignancy, or patients receiving cancer treatment for their malignancy beyond six months. Moreover, in the Netherlands anticoagulation is often continued after six months of initial treatment in case the cancer is still active. Unfortunately, we were unable to assess the effect of continued anticoagulation treatment due to lack of data. However, since rivaroxaban is associated with cost-saving results during the first six months, it is to be expected that during a longer treatment period the cost-savings and health gains will accrue even more compared with dalteparin.

For peer review only - http://bmjopen.bmj.com/site/about/guidelines.xhtml

In the univariate sensitivity analysis, we have shown that the risk of MB and VTE for both rivaroxaban and dalteparin have a high influence on the incremental costs and QALYs. In the SELECT-D trial [15], the incidence of symptomatic and fatal PE events was relatively higher in patients treated with rivaroxaban. However, due to low numbers of VTE observed in the SELECT-D trial [15], we calculated the distribution of the type of VTE based on the total number of events and assumed it to be equal for both drugs. This may have led to an overestimation of the effect of rivaroxaban compared with dalteparin, since symptomatic and fatal PE events have a higher impact on the costs and the patient's health compared to DVT and incidental PE. On the other hand, we used this same approach to calculate the distributions of the types of MB from the HOKUSAI VTE Cancer trial [16], in which the patients treated with dalteparin had relatively more severe MB events compared with the NOAC edoxaban (ICH: 17.6% versus 6.1%, respectively). This results in an underestimation of the number of MBs in dalteparin-treated patients. We assessed the effect of using drug-specific distributions of the type of VTE and MB in scenario six, showing an increase in incremental cost-savings and QALYs compared to the base case analysis. Therefore, we conclude that our approach of using equal distributions of the types of VTE and MB for rivaroxaban and dalteparin is conservative.

This study focuses on the secondary prevention of VTE, based on the results of the SELECT-D and, partially, the HOKUSAI VTE Cancer trials. However, recently, apixaban was also assessed in cancer patients at risk of recurrent VTE and found to be non-inferior compared to dalteparin [38,39]. Moreover, the AVERT and CASSINI trials have shown that apixaban and rivaroxaban are also effective as a primary prophylaxis of VTE in cancer patients compared with a placebo [40–42]. Based on these two studies, clinicians may consider DOAC prophylaxis in some of their cancer patients [42]. Therefore, future research is needed to assess if DOACs are also cost-effective for the primary prevention of VTE.

Conclusion

Treatment with rivaroxaban is dominant (cost-saving while slightly improving the patient's health and quality of life) over dalteparin in cancer patients at risk for recurrent VTE in the Netherlands. The use of rivaroxaban instead of LMWH (including dalteparin) can save more than eleven million euros per year, which is primarily driven by the difference in treatment costs. Since treatment with rivaroxaban is economically dominant compared with dalteparin and its oral administration is more convenient than daily subcutaneous injection, it is logical that certain cancer patients can benefit from DOAC treatment and provide savings to the healthcare system.

References

1. Gerotziafas GT, Mahé I, Elalamy I. Therapeutics and Clinical Risk Management Dovepress New orally active anticoagulant agents for the prevention and treatment of venous thromboembolism in cancer patients. Ther Clin Risk Manag. 2014;10:423–36.
2. Falanga A, Marchetti M, Vignoli A. Coagulation and cancer: Biological and clinical aspects. J Thromb Haemost. 2013;11(2):223–33.
3. Lechner D, Weltermann A. Chemotherapy-induced thrombosis: a role for microparticles and tissue factor? Semin Thromb Hemost. 2008;34(2):199–203.
4. Cohen AT, Katholing A, Rietbrock S, Bamber L, Martinez C. Epidemiology of first and recurrent venous thromboembolism in patients with active cancer. Thromb Haemost. 2017;117(01):57–

For peer review only - http://bmjopen.bmj.com/site/about/guidelines.xhtml

65.

5. Minister of Health W and S. Letter to the parlement - reimbursement rivaroxaban VTE (Herbeoordeling uitbreiding nadere voorwaarden rivaroxaban (Xarelto®) bij VTE. 2015;

6. Steffel J, Verhamme P, Potpara TS, Albaladejo P, Antz M, Desteghe L, et al. The 2018 European Heart Rhythm Association Practical Guide on the use of non-vitamin K antagonist oral anticoagulants in patients with atrial fibrillation. Eur Heart J. 2018;

7. Mandala M, Falanga A, Roila F, ESMO Guidelines Working Group. Management of venous thromboembolism (VTE) in cancer patients: ESMO Clinical Practice Guidelines. Ann Oncol. 2011;22(Supplement 6):vi85–92.

8. Key NS, Khorana AA, Kuderer NM, Bohlke K, Lee AYY, Arcelus JI, et al. Venous thromboembolism prophylaxis and treatment in patients with cancer: ASCO clinical practice guideline update. J Clin Oncol. 2020;38(5):496–520.

9. Farge D, Frere C, Connors JM, Ay C, Khorana AA, Munoz A, et al. 2019 International Clinical Practice Guidelines for the Treatment and Prophylaxis of Venous Thromboembolism in Patients With Cancer. Lancet Oncol. 2019;20(10):e566–81.

10. Konstantinides S V., Meyer G, Bueno H, Galié N, Gibbs JSR, Ageno W, et al. 2019 ESC Guidelines for the diagnosis and management of acute pulmonary embolism developed in collaboration with the European respiratory society (ERS). Eur Heart J. 2020;41(4):543–603.

11. Streiff MB, Holmstrom B, Angelini D, Ashrani A, Bockenstedt PL, Chesney C, et al. NCCN Guidelines® insights cancer-associated venous thromboembolic disease, version 2.2018 featured updates to the NCCN guidelines. JNCCN J Natl Compr Cancer Netw. 2018;16(11):1289–303.

12. Hull RD, Pineo GF, Brant RF, Mah AF, Burke N, Dear R, et al. Long-term low-molecular-weight heparin versus usual care in proximal-vein thrombosis patients with cancer. Am J Med. 2006 Dec;119(12):1062–72.

13. Lee AYY, Levine MN, Baker RI, Bowden C, Kakkar AK, Prins M, et al. Low-Molecular-Weight Heparin versus a Coumarin for the Prevention of Recurrent Venous Thromboembolism in Patients with Cancer. N Engl J Med. 2003;349(2):146–53.

14. Meyer G, Marjanovic Z, Valcke J, Lorcerie B, Gruel Y, Solal-Celigny P, et al. Comparison of low-molecular-weight heparin and warfarin for the secondary prevention of venous thromboembolism in patients with cancer: a randomized controlled study. Arch Intern Med. 162(15):1729–35.

15. Young AM, Marshall A, Thirlwall J, Chapman O, Lokare A, Hill C, et al. Comparison of an Oral Factor Xa Inhibitor With Low Molecular Weight Heparin in Patients With Cancer With Venous Thromboembolism: Results of a Randomized Trial (SELECT-D). J Clin Oncol. 2018;36(20):2017–23.

16. Raskob GE, van Es N, Verhamme P, Carrier M, Di Nisio M, Garcia D, et al. Edoxaban for the Treatment of Cancer-Associated Venous Thromboembolism. N Engl J Med. 2018;378(7):615–24.

17. Streiff MB, Milentijevic D, McCrae K, Yannicelli D, Fortier J, Nelson WW, et al. Effectiveness and safety of anticoagulants for the treatment of venous thromboembolism in patients with cancer. Am J Hematol. 2018;93(5):664–71.

18. Dutch Institute National Health Care (Zorginstituut Nederland). Richtlijn voor het uitvoeren van economische evaluaties in de gezondheidzorg (Protocol for the execution of economic evaluation in healthcare). 29-02-2016. 2016;(november):120.

19. National Institute of Public Health and the Environment (RIVM). Cancer numbers and context: current situation [Internet]. volksgezondheidenzorg.info. 2017. p. 1.

20. Briggs A, Claxton K, Sculpher M. Decision modelling for Health Economic Evaluation. 3rd ed. New York: Oxford University Press; 2011. 57–58 p.

21. The Netherlands Cancer Registry. Survival statistics - all tumours 1961-2015 [Internet].

22. Heisen M, Treur MJ, Heemstra HE, Giesen EBW, Postma MJ. Cost-effectiveness analysis of rivaroxaban for treatment and secondary prevention of venous thromboembolism in the

For peer review only

For peer review only - http://bmjopen.bmj.com/site/about/guidelines.xhtml

Netherlands. J Med Econ. 2017 Aug 3;20(8):813–24.
23. Medicijnkosten.nl. The National Health Care Institute (ZIN) [Internet].
24. Summary of Product Characteristics Fragmin. 2015;
25. Dutch Statistics. Length and weight of Dutch population [Internet]. 2017.
26. de Klerk M, de Boer A, Plaisier I, Schyns P, Kooiker S. Informele hulp: wie doet er wat? - Rapport - SCP [Internet]. 2015-35. 2015.
27. Hakkaart-van Roijen L, van der Linden N, Bouwmans C, Kanters T, Swan Tan S. Kostenhandleiding: Methodologie van kostenonderzoek en referentieprijzen voor economische evaluaties in de gezondheidszorg. Zorginstituut Ned. 2016;1–120.
28. Lloyd AJ, Dewilde S, Noble S, Reimer E, Lee AYY. What Impact Does Venous Thromboembolism and Bleeding Have on Cancer Patients' Quality of Life? Value Heal. 2018;21(4):449–55.
29. Roman A, Barbera JA, Castillo MJ, Muñoz R, Escribano P. Health-related quality of life in a national cohort of patients with pulmonary arterial hypertension or chronic thromboembolic pulmonary hypertension. Arch Bronconeumol. 2013;49(5):181–8.
30. Stevanović J, de Jong LA, Kappelhoff BS, Dvortsin EP, Voorhaar M, Postma MJ. Dabigatran for the Treatment and Secondary Prevention of Venous Thromboembolism; A Cost-Effectiveness Analysis for the Netherlands. PLoS One. 2016;11(10):e0163550.
31. The Netherlands Cancer Registry. Prevalence statistics - all tumours [Internet].
32. Walker AJ, Card TR, West J, Crooks C, Grainge MJ. Incidence of venous thromboembolism in patients with cancer - a cohort study using linked United Kingdom databases. Eur J Cancer. 2013 Apr;49(6):1404–13.
33. Streiff M, Milentijevic D, McCrae KR, Laliberté F, Lejeune D, Lefebvre P, et al. Healthcare resource utilization and costs associated with venous thromboembolism in cancer patients treated with anticoagulants. J Med Econ. 2019;22(11):1134–40.
34. Aapro M, Launay-Vacher V. Importance of monitoring renal function in patients with cancer. Cancer Treat Rev. 2012;38(3):235–40.
35. Vedham V, Divi RL, Starks VL, Verma M. Multiple Infections and Cancer: Implications in Epidemiology. Technol Cancer Res Treat. 2014;13(2):177–94.
36. Mahé I, Chidiac J, Helfer H, Noble S. Factors influencing adherence to clinical guidelines in the management of cancer-associated thrombosis. J Thromb Haemost. 2016;14(11):2107–13.
37. Committee for Medicinal Products for Human Use (CHMP). Summary of product characteristics - rivaroxaban 20 mg [Internet].
38. Agnelli G, Becattini C, Meyer G, Muñoz A, Huisman M V., Connors JM, et al. Apixaban for the treatment of venous thromboembolism associated with cancer. N Engl J Med. 2020;382(17):1599–607.
39. McBane RD, Wysokinski WE, Le-Rademacher JG, Zemla T, Ashrani A, Tafur A, et al. Apixaban and dalteparin in active malignancy-associated venous thromboembolism: The ADAM VTE trial. J Thromb Haemost. 2020;18(2):411–21.
40. Khorana AA, Soff GA, Kakkar AK, Vadhan-Raj S, Riess H, Wun T, et al. Rivaroxaban for Thromboprophylaxis in High-Risk Ambulatory Patients with Cancer. N Engl J Med. 2019 Feb;380(8):720–8.
41. Carrier M, Abou-Nassar K, Mallick R, Tagalakis V, Shivakumar S, Schattner A, et al. Apixaban to Prevent Venous Thromboembolism in Patients with Cancer. N Engl J Med. 2019;380(8):711–9.
42. Agnelli G. Direct Oral Anticoagulants for Thromboprophylaxis in Ambulatory Patients with Cancer. N Engl J Med. 2019;380(8):781–3.

Author contributions

Lisa A. de Jong built the economic model, performed the analyses, and contributed to the design of the work, interpretation of the results, writing of the manuscript.

For peer review only

For peer review only - http://bmjopen.bmj.com/site/about/guidelines.xhtml

Annette W.G. van der Velden contributed to the interpretation of the results, writing of the manuscript, and critical revision for important intellectual content.
Marinus van Hulst contributed to the design of the work, interpretation of the results, writing of the manuscript, and critical revision for important intellectual content.
Maarten J. Postma contributed to the design of the work, interpretation of the results, writing of the manuscript, and critical revision for important intellectual content.
All authors approved of the version to be published.

Checklist for the appropriate reporting statement: This manuscript was written in accordance with the CHEERS checklist for reporting economic evaluations of health interventions.

Data sharing statement: All relevant data are included in the manuscript. The analyses were conducted based on publicly available information which is presented and referenced in the manuscript.

Word count: 5,061 (excluding table and figure descriptions and references)

Patient consent and ethical approval: The analyses were conducted based on publicly available information which is presented and referenced in the article and Supporting Information files, and did therefore not require any patient consent forms or approval from an ethical review board.

Patient and public involvement statement: It was not appropriate to involve patients or the public in the design, or conduct, or reporting, or dissemination plans of our research, because this health economic analysis was based on publicly available data and solely concentrated on the analysis of the economics consequence of treating cancer patients with rivaroxaban instead of the current standard of care.

For peer review only - http://bmjopen.bmj.com/site/about/guidelines.xhtml

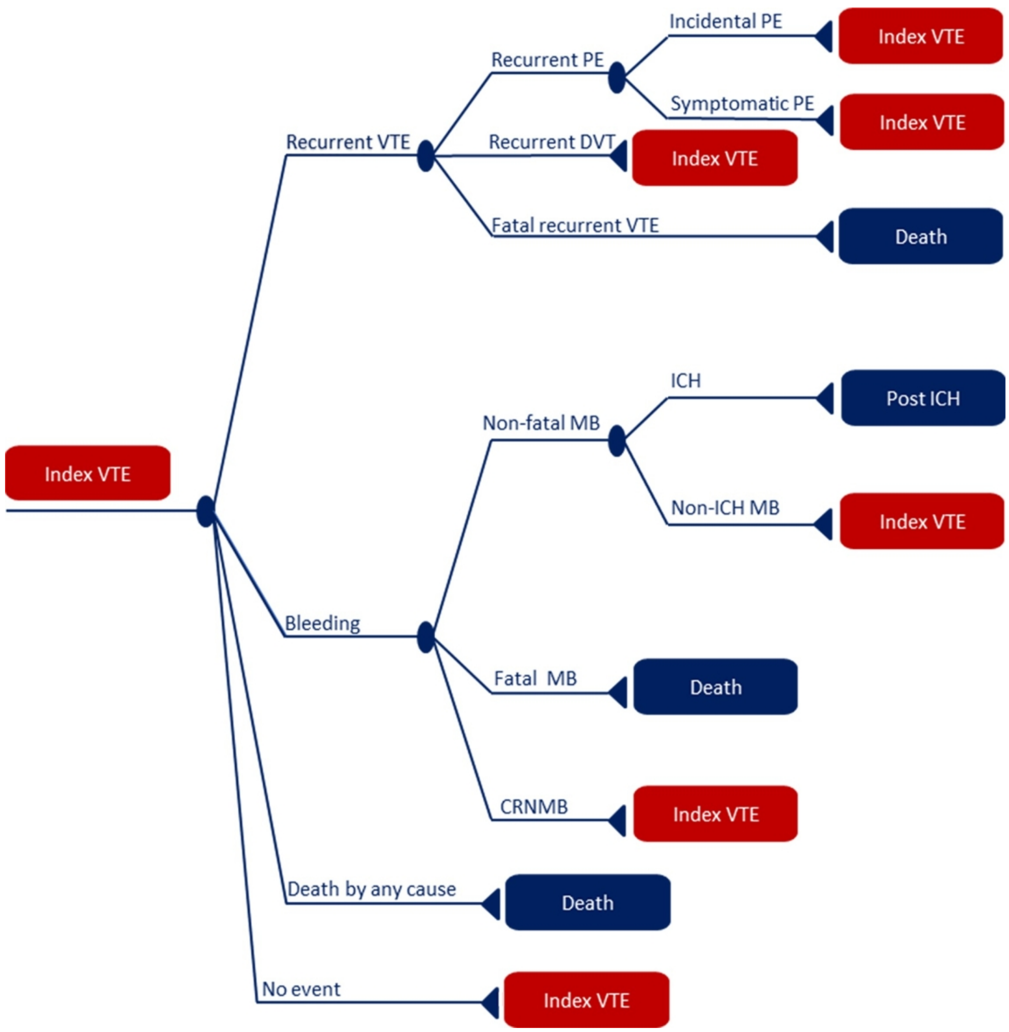

Figure 1. Model outline. All patients enter the model in the 'Index VTE' state and move to other states upon the occurrence of one of the following events: recurrent incidental PE, recurrent symptomatic PE, fatal recurrent VTE, recurrent DVT, ICH, non-ICH MB, fatal MB, CRNMB, or death by any cause. The triangles represent the health state a patient will enter after an event. The blue squares are permanent states, in which a patient will remain until death while not being at risk for other events. The red squares represent a transient state: the patient will re-enter the model in the 'Index VTE' state.
Abbreviations: CRNMB, clinically relevant non-major bleeding; DVT, deep vein thrombosis; ICH, intracranial haemorrhage; MB, major bleeding; PE, pulmonary embolism; VTE, venous thromboembolism

74x75mm (600 x 600 DPI)

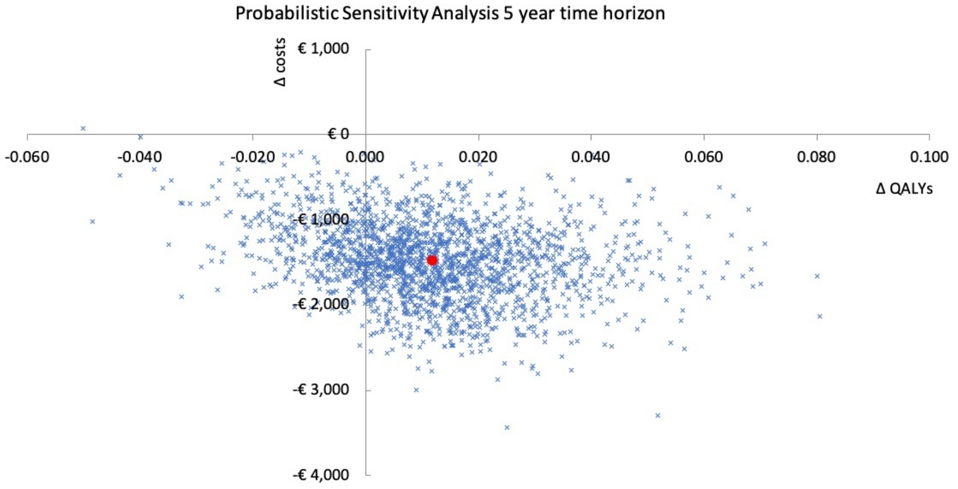

Figure 2. Probabilistic sensitivity analysis of the base case with five-year time horizon (base case analysis). The red mark represents the deterministic incremental cost-effectiveness ratio. Abbreviation: QALY, quality adjusted life-year

206x112mm (144 x 144 DPI)

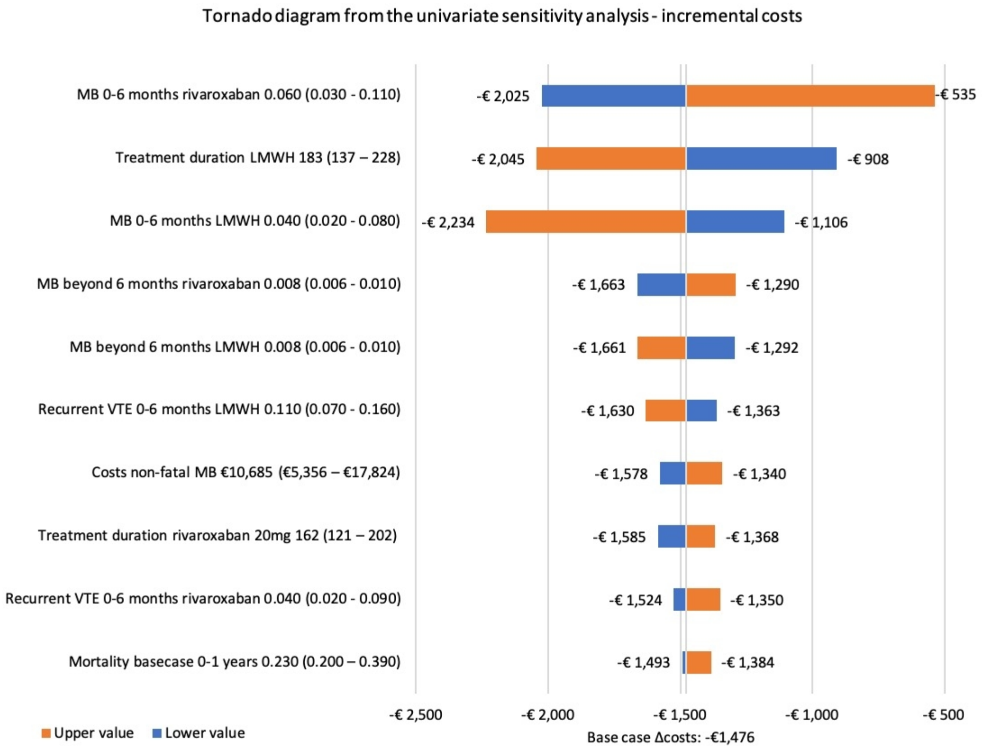

Figure 3. Tornado diagram from the univariate sensitivity analysis for the base case analysis showing the impact of parameters on the incremental costs. Abbreviations: ICH, intracranial haemorrhage; MB, major bleeding; PE, pulmonary embolism; VTE, venous thromboembolism

195x149mm (144 x 144 DPI)

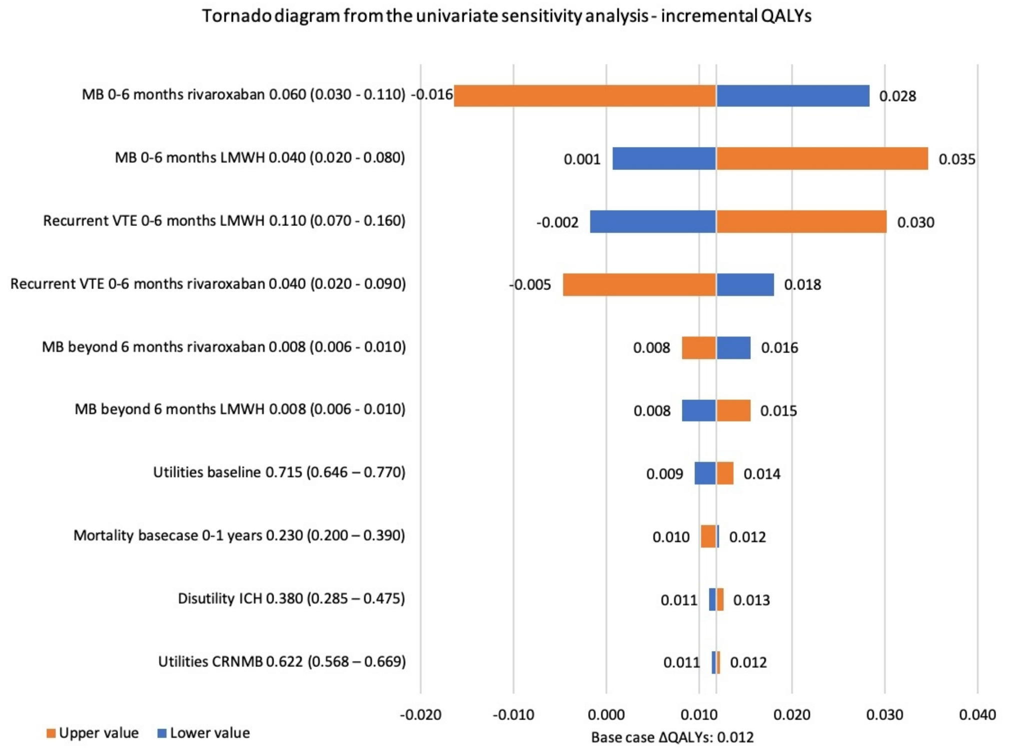

Figure 4. Tornado diagram from the univariate sensitivity analysis for the base case analysis showing the impact of parameters on the incremental QALYs. Abbreviations: CRNMB, clinically relevant non-major bleeding; ICH, intracranial haemorrhage; MB, major bleeding; VTE, venous thromboembolism

195x149mm (144 x 144 DPI)

Supplementary data file – Table S1

Manuscript title: Cost-effectiveness analysis and budget impact of rivaroxaban in cancer patients at risk of recurrent venous thromboembolism

Table S1. Transition probabilities used in the cost-effectiveness model

	Rivaroxaban (95% CI)	LMWH (95% CI)	Distribution	Reference
**Recurrent VTE**				
0–6 months	0.040 (0.020 – 0.090)	0.110 (0.070 – 0.160)	Beta	[1]
6–12 months	0.040 (0.031 – 0.050)		Beta	[2]
1–2 years	0.034 (0.027 – 0.042)		Beta	[2]
2–3 years	0.021 (0.014 – 0.029)		Beta	[2]
3–4 years	0.016 (0.009 – 0.026)		Beta	[2]
4–5 months	0.013 (0.006 – 0.024)		Beta	[2]
**Type of recurrent VTE**				
Symptomatic PE	17.4% (α = 4, β = 19)		Dirichlet	[1]
Incidental PE	30.4% (α = 7, β = 16)		Dirichlet	[1]
DVT	43.5% (α = 10, β = 13)		Dirichlet	[1]
Fatal PE	8.7% (α = 2, β = 21)		Dirichlet	[1]
**MB**				
0–6 months	0.060 (0.030 – 0.110)	0.040 (0.020 – 0.080)	Beta	[1]
Beyond 6 months treatment	0.008 (0.006 – 0.010)		Beta	[3]
**Type of MB**				
ICH	10% (α = 5, β = 45)		Dirichlet	[3]
Non-ICH MB	86% (α = 43, β = 7)		Dirichlet	[3]
Fatal MB	4% (α = 2, β = 48)		Dirichlet	[3]
**CRNMB**				
0–6 months	0.130 (0.090 – 0.190)	0.040 (0.020 – 0.090)	Beta	[1]
Beyond 6 months treatment	0.008 (0.006 – 0.010)		Beta	[3]
**PTS**				
0–6 months	0.015 (0.011 – 0.019)		Beta	[4]
6–12 months	0.012 (0.009 – 0.015)		Beta	[4]
12–18 months	0.008 (0.006 – 0.010)		Beta	[4]
18–24 months	0.025 (0.023 – 0.019)		Beta	[4]
24–30 months	0.011 (0.008 – 0.014)		Beta	[4]
30–36 months	0.006 (0.005 – 0.008)		Beta	[4]
3–4 years	0.001 (0.0008 – 0.0013)		Beta	[4]
4–5 years	0.001 (0.0008 – 0.0013)		Beta	[4]
**CTEPH (annual risk)**	0.0057 (0.0002 – 0.012)		Beta	[5]
**Mortality (annual risk)**				
0–1 years	0.230 (0.200 – 0.390)		Beta	[6]
1–2 years	0.104 (0.088 – 0.180)		Beta	[6]
2–3 years	0.058 (0.055 – 0.120)		Beta	[6]
3–4 years	0.046 (0.043 – 0.068)		Beta	[6]
4–5 years	0.032 (0.030 – 0.073)		Beta	[6]
**Relative risk of recurrent VTE, MB, and CRNMB for LMWH versus placebo, used in scenario 5**				
Recurrent VTE (any)	5.170		Fixed	[7]
MB	0.242		Fixed	[7]
CRNMB	1.000		Fixed	[7]

For peer review only

**Drug-specific distribution of the type of VTE, used in scenario 6**				
Symptomatic PE	28.6% (α = 2, β = 5)	12.5% (α = 2, β = 14)	Dirichlet	[1]
Incidental PE	14.3% (α = 1, β = 6)	37.5% (α = 6, β = 10)	Dirichlet	[1]
DVT	42.9% (α = 3, β = 4)	43.8% (α = 7, β = 9)	Dirichlet	[1]
Fatal PE	14.3% (α = 1, β = 6)	6.3% (α = 1, β = 15)	Dirichlet	[1]
**Drug-specific distribution of the type of MB, used in scenario 6**				
ICH	6.1% (α = 2, β = 31)	17.6% (α = 3, β = 14)	Dirichlet	[3]
Non-ICH MB	93.9% (α = 31, β = 2)	70.6% (α = 12, β = 5)	Dirichlet	[3]
Fatal MB	0% (α = 0, β = 33)	11.8% (α = 2, β = 15)	Dirichlet	[3]

Abbreviations: CI, confidence interval; CRNMB, clinically relevant non-major bleeding; CTEPH, chronic thromboembolic pulmonary hypertension; DVT, deep vein thrombosis; ICH, intracranial haemorrhage; LMWH, low-molecular weight heparin; MB, major bleeding; PE, pulmonary embolism; PTS, post-thrombotic syndrome; SE, standard error; VTE, venous thromboembolism

References

1. Young AM, Marshall A, Thirlwall J, Chapman O, Lokare A, Hill C, et al. Comparison of an Oral Factor Xa Inhibitor With Low Molecular Weight Heparin in Patients With Cancer With Venous Thromboembolism: Results of a Randomized Trial (SELECT-D). J Clin Oncol. 2018;36(20):2017–23.
2. Cohen AT, Katholing A, Rietbrock S, Bamber L, Martinez C. Epidemiology of first and recurrent venous thromboembolism in patients with active cancer. Thromb Haemost. 2017;117(01):57–65.
3. Raskob GE, van Es N, Verhamme P, Carrier M, Di Nisio M, Garcia D, et al. Edoxaban for the Treatment of Cancer-Associated Venous Thromboembolism. N Engl J Med. 2018;378(7):615–24.
4. Prandoni P, Villalta S, Bagatella P, Rossi L, Marchiori A, Piccioli A, et al. The clinical course of deep-vein thrombosis. Prospective long-term follow-up of 528 symptomatic patients. Haematologica. 1997;82(4):423–8.
5. Klok FA, van Kralingen KW, van Dijk APJ, Heyning FH, Vliegen HW, Huisman M V. Prospective cardiopulmonary screening program to detect chronic thromboembolic pulmonary hypertension in patients after acute pulmonary embolism. Haematologica. 2010;95(6):970–5.
6. The Netherlands Cancer Registry. Survival statistics - all tumours 1961-2015 [Internet].
7. Napolitano M, Saccullo G, Malato A, Sprini D, Ageno W, Imberti D, et al. Optimal duration of low molecular weight heparin for the treatment of cancer-related deep vein thrombosis: the Cancer-DACUS Study. J Clin Oncol. 2014;32(32):3607–12.

For peer review only

Supplementary data file – Table S2

Manuscript title: Cost-effectiveness analysis and budget impact of rivaroxaban in cancer patients at risk of recurrent venous thromboembolism

Table S2. Costs included in the cost-effectiveness model (Euros, 2019)

	Value (95% CI)	Distribution	Reference
**Event costs**			
Recurrent VTE			
Symptomatic PE	€4,717 (€2,364 – €7,868)	Gamma	[1]
Incidental PE	€0	Fixed	Assumption
DVT	€663 (€464 – €862)	Gamma	[1]
Fatal recurrent VTE [a]	€4,717 (€2,364 – €7,868)	Gamma	[1]
ICH acute care costs	€22,769 (€11,644 – €31,175)	Gamma	[2]
ICH long-term costs (monthly)	€637 (€319 – €1,063)	Gamma	[1]
Non-ICH MB	€10,685 (€5,356 – €17,824)	Gamma	[1]
Fatal MB	€10,685 (€5,356 – €17,824)	Gamma	[1]
CRNMB	€274 (€137 – €457)	Gamma	[1]
PTS	€1,431 (€717 – €2,387)	Gamma	[1]
CTEPH acute care costs	€7,843 (€3,931 – €16,433)	Gamma	[1]
CTEPH long-term costs (monthly)	€89 (€45 – €149)	Gamma	[1]
**Treatment costs**			
Drug cost (daily)			
LMWH [b]	€9.93	Fixed	[3]
Rivaroxaban 15 mg	€4.58	Fixed	[3]
Rivaroxaban 20 mg	€2.29	Fixed	[3]
Treatment duration (days)			
LMWH	183 (137 – 228)	Gamma	[4]
Rivaroxaban 15 mg	21 (16 – 26)	Gamma	[4]
Rivaroxaban 20 mg	162 (121 – 202)	Gamma	[4]
LMWH administration costs			
Costs for home caregiver (per hour)	€59.34 (€44.51 – €74.18)	Gamma	[5]
Duration of at home administration (hour)	0.25 (0.19 – 0.31)	Gamma	Assumption
Hospitalisation duration PE (days) [c]	6.6 (5.0 – 8.3)	Gamma	[6]
Renal monitoring [c]	€1.64 (€1.23 – €2.05)	Gamma	[7]
**Indirect costs**			
Travel costs			
Cost per km	€0.20 (€0.15 – €0.25)	Gamma	[8]
Distance to hospital (km)	7	Fixed	[8]
Distance to GP (km)	1.1	Fixed	[8]
Informal care costs			
PE	€1,515 (€1,136 – €1,894)	Gamma	[5,9]
DVT	€233 (€175 – €291)	Gamma	[5,9]
ICH (acute informal care costs)	€1,515 (€1,136 – €1,894)	Gamma	[5,9]
ICH (long-term informal care costs, monthly)	€626 (€470 – €783)	Gamma	[10]
Non-ICH MB	€758 (€568 – €947)	Gamma	[5,9]
CRNMB	€117 (€87 – €146)	Gamma	[5,9]

Abbreviations: CI, confidence interval; CRNMB, clinically relevant non-major bleeding; CTEPH, chronic thromboembolic pulmonary hypertension; DVT, deep vein thrombosis; GP, general practitioner; ICH, intracranial haemorrhage; LMWH, low-molecular weight heparin; MB, major bleeding; PE, pulmonary embolism; PTS, post-thrombotic syndrome; VTE, venous thromboembolism

[a] Assumed to be equal to the costs of non-fatal PE

For peer review only

b Based on an average weight between 69 and 82 kg.

c Based on DRG code 070419 and only taken into account for rivaroxaban treated patients

References

1. Heisen M, Treur MJ, Heemstra HE, Giesen EBW, Postma MJ. Cost-effectiveness analysis of rivaroxaban for treatment and secondary prevention of venous thromboembolism in the Netherlands. J Med Econ. 2017 Aug 3;20(8):813–24.
2. Baeten S a, van Exel NJ a, Dirks M, Koopmanschap M a, Dippel DW, Niessen LW. Lifetime health effects and medical costs of integrated stroke services - a non-randomized controlled cluster-trial based life table approach. Cost Eff Resour Alloc. 2010;8(1):21.
3. Medicijnkosten.nl. The National Health Care Institute (ZIN) [Internet].
4. Young AM, Marshall A, Thirlwall J, Chapman O, Lokare A, Hill C, et al. Comparison of an Oral Factor Xa Inhibitor With Low Molecular Weight Heparin in Patients With Cancer With Venous Thromboembolism: Results of a Randomized Trial (SELECT-D). J Clin Oncol. 2018;36(20):2017–23.
5. Hakkaart-van Roijen L, van der Linden N, Bouwmans C, Kanters T, Swan Tan S. Kostenhandleiding: Methodologie van kostenonderzoek en referentieprijzen voor economische evaluaties in de gezondheidszorg. Zorginstituut Ned. 2016;1–120.
6. van Bellen B, Bamber L, Correa de Carvalho F, Prins M, Wang M, Lensing AWA. Reduction in the length of stay with rivaroxaban as a single-drug regimen for the treatment of deep vein thrombosis and pulmonary embolism. Curr Med Res Opin. 2014;30(5):829–37.
7. Dutch Health Authorities. NZa zorgproductapplicatie. Declaration code: 070419. [Internet]. 2018.
8. Roijen LH, Linden N van der, Bouwmans C, Kanters T, Tan SS. Dutch manual for costing studies in health care. Diemen; 2015.
9. de Klerk M, de Boer A, Plaisier I, Schyns P, Kooiker S. Informele hulp: wie doet er wat? - Rapport - SCP [Internet]. 2015-35. 2015.
10. van den Berg B, Brouwer W, van Exel J, Koopmanschap M, van den Bos GAM, Rutten F. Economic valuation of informal care: lessons from the application of the opportunity costs and proxy good methods. Soc Sci Med. 2006;62(4):835–45.

For peer review only

Supplementary data file – Table S3

Manuscript title: Cost-effectiveness analysis and budget impact of rivaroxaban in cancer patients at risk of recurrent venous thromboembolism

Table S3. Utility values included in the cost-effectiveness model

	Value (95% CI)	Distribution	Reference
**Utilities**			
Index VTE			
0–1 month	0.565 (0.501 – 0.620)	Beta	[1]
1–2 months	0.655 (0.585 – 0.713)	Beta	[1]
2–3 months	0.674 (0.606 – 0.729)	Beta	[1]
3–4 months	0.698 (0.635 – 0.750)	Beta	[1]
4–5 months	0.707 (0.645 – 0.758)	Beta	[1]
Baseline utility 6 months after index VTE	0.715 (0.646 – 0.770)	Beta	[1]
Recurrent VTE			
DVT	0.605 (0.514 – 0.678)	Beta	[1]
Non-fatal symptomatic PE	0.621 (0.477 – 0.725)	Beta	[1]
Non-fatal incidental PE	0.664 (0.615 – 0.707)	Beta	[1]
Non-ICH MB	0.593 (0.461 – 0.693)	Beta	[1]
CRNMB	0.622 (0.568 – 0.669)	Beta	[1]
**Utility decrements**			
Recurrent VTE within first six months after index VTE			
DVT	0.040 (0.000 – 0.158)	Beta	[1]
Symptomatic PE	0.024 (0.000 – 0.195)	Beta	[1]
Incidental PE	0.189 (0.021 – 0.404)	Beta	[1]
ICH	0.380 (0.285 – 0.475)	Beta	[2]
Severe PTS (<6 months after diagnosis)	0.186 (0.090 – 0.280)	Beta	[1]
Severe PTS (>6 months after diagnosis)	0.070 (0.053 – 0.088)	Beta	[2]
CTEPH			
0-1 year	0.194 (0.071 – 0.303)	Beta	[3]
1–4 years	0.109 (0.000 – 0.244)	Beta	[3]
4–5 years	0.079 (0.000 – 0.277)	Beta	[3]

Abbreviations: CI, confidence interval; CRNMB, clinically relevant non-major bleeding; CTEPH, chronic thromboembolic pulmonary hypertension; DVT, deep vein thrombosis; ICH, intracranial haemorrhage; MB, major bleeding; PE, pulmonary embolism; PTS, post-thrombotic syndrome; VTE, venous thromboembolism

References

1. Lloyd AJ, Dewilde S, Noble S, Reimer E, Lee AYY. What Impact Does Venous Thromboembolism and Bleeding Have on Cancer Patients' Quality of Life? Value Heal. 2018;21(4):449–55.
2. Stevanović J, de Jong LA, Kappelhoff BS, Dvortsin EP, Voorhaar M, Postma MJ. Dabigatran for the Treatment and Secondary Prevention of Venous Thromboembolism; A Cost-Effectiveness Analysis for the Netherlands. PLoS One. 2016;11(10):e0163550.
3. Roman A, Barbera JA, Castillo MJ, Muñoz R, Escribano P. Health-related quality of life in a national cohort of patients with pulmonary arterial hypertension or chronic thromboembolic pulmonary hypertension. Arch Bronconeumol. 2013;49(5):181–8.

For peer review only

Supplementary data file – Figure S1

Manuscript title: Cost-effectiveness analysis and budget impact of rivaroxaban in cancer patients at risk of recurrent venous thromboembolism

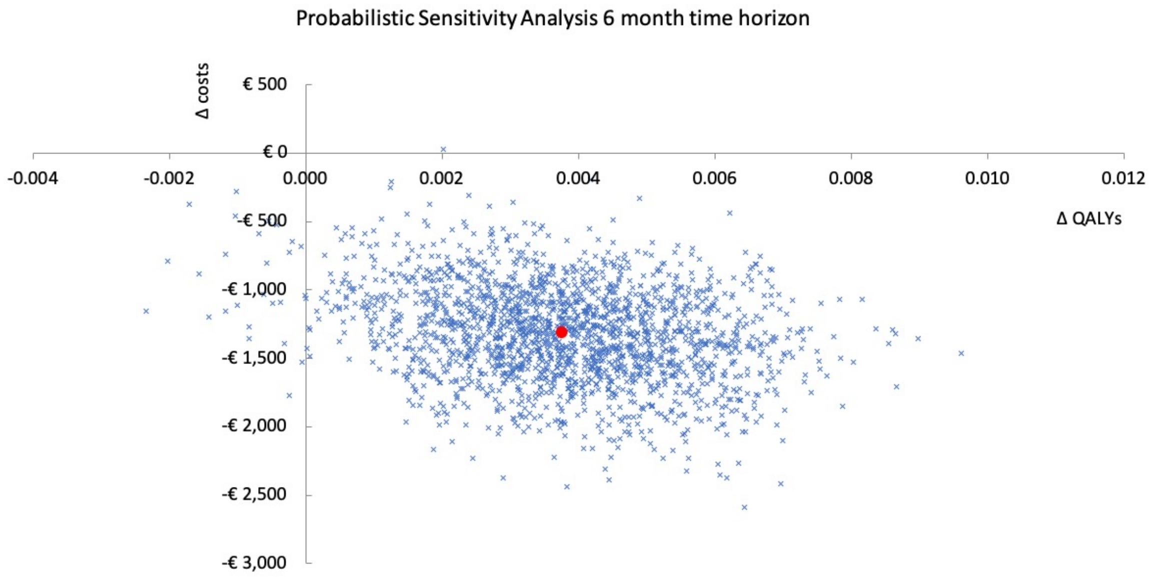

Figure S1. Probabilistic sensitivity analysis with six-month time horizon (scenario 1). *The red mark represents the deterministic incremental cost-effectiveness ratio. Abbreviation: QALY, quality adjusted life-year*

Supplementary data file – Figure S2

Manuscript title: Cost-effectiveness analysis and budget impact of rivaroxaban in cancer patients at risk of recurrent venous thromboembolism

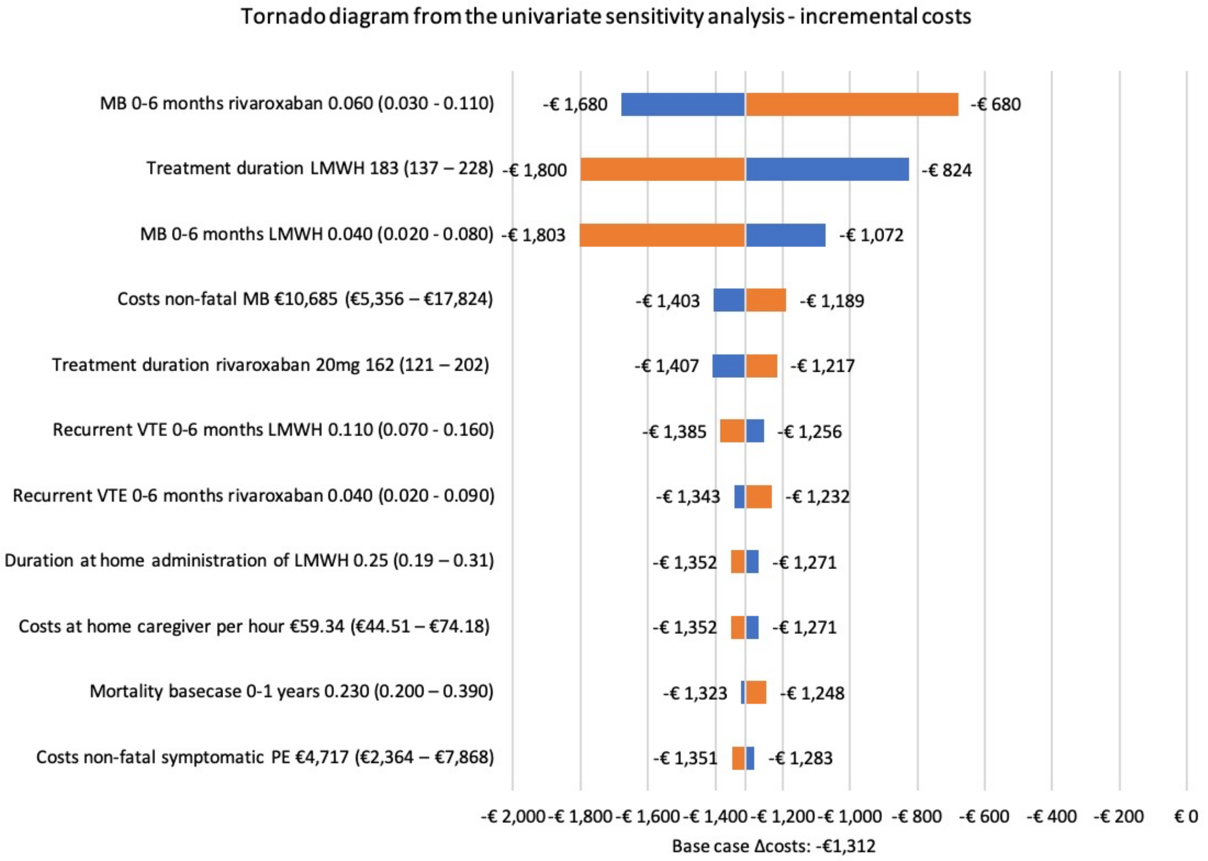

Figure S2. Tornado diagram from the univariate sensitivity analysis for scenario 1 showing the impact of parameters on the incremental costs. *Abbreviations: MB, major bleeding; PE, pulmonary embolism; VTE, venous thromboembolism*

For peer review only

Supplementary data file – Figure S3

Manuscript title: Cost-effectiveness analysis and budget impact of rivaroxaban in cancer patients at risk of recurrent venous thromboembolism

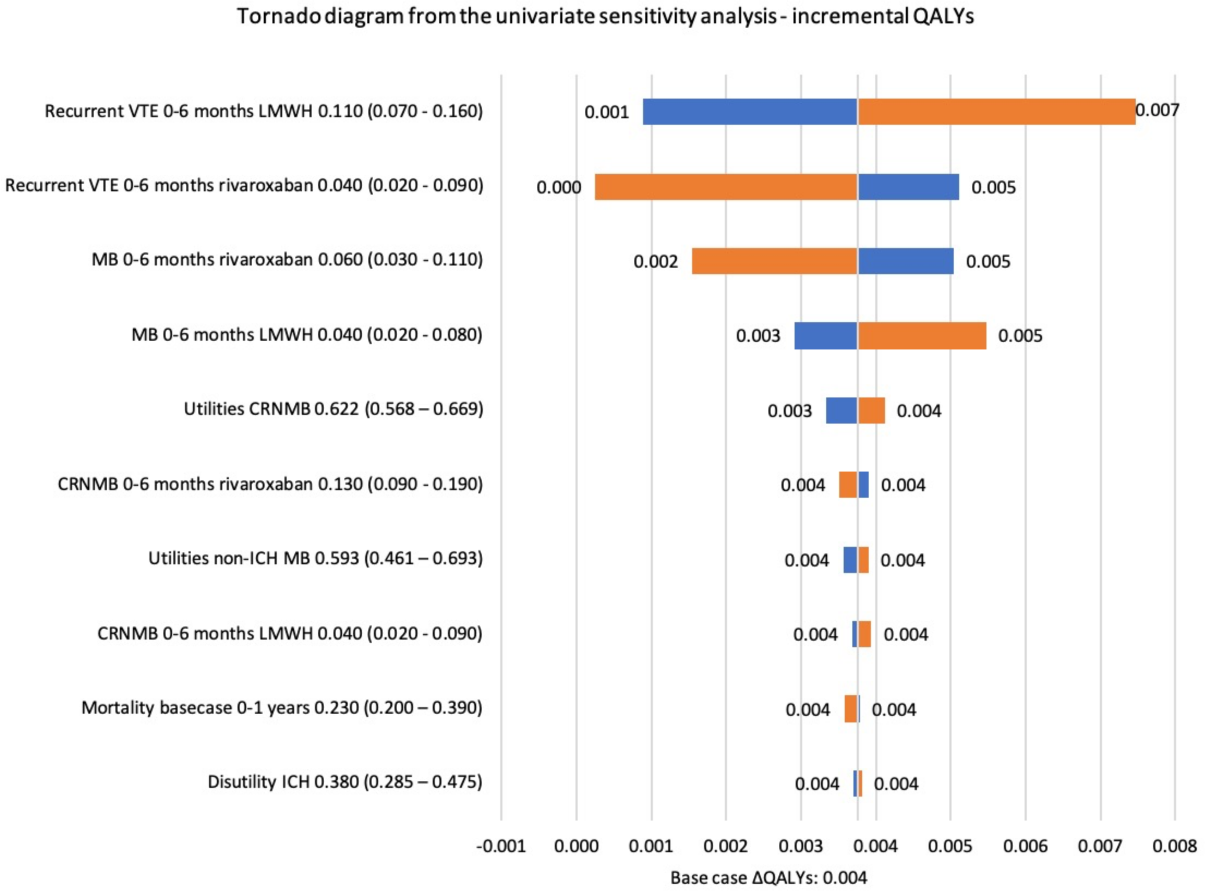

Figure S3. Tornado diagram from the univariate sensitivity analysis for scenario 1 showing the impact of parameters on the incremental QALYs. *Abbreviations: CRNMB, clinically relevant non-major bleeding; ICH, intracranial haemorrhage; MB, major bleeding; PE, pulmonary embolism; VTE, venous thromboembolism*

CHEERS checklist—Items to include when reporting economic evaluations of health interventions

Section/item	Item No	Recommendation	Reported on page No/ line No
**Title and abstract**			
Title	1	Identify the study as an economic evaluation or use more specific terms such as "cost-effectiveness analysis", and describe the interventions compared.	Page 1, line 9-10
Abstract	2	Provide a structured summary of objectives, perspective, setting, methods (including study design and inputs), results (including base case and uncertainty analyses), and conclusions.	Page 1, line 34-64
**Introduction**			
Background and objectives	3	Provide an explicit statement of the broader context for the study.	Page 4, line 91-120
		Present the study question and its relevance for health policy or practice decisions.	Page 4, line 121-126
**Methods**			
Target population and subgroups	4	Describe characteristics of the base case population and subgroups analysed, including why they were chosen.	Page 5, line 149-151<br>Page 5, line 179
Setting and location	5	State relevant aspects of the system(s) in which the decision(s) need(s) to be made.	Page 4, line 130-140
Study perspective	6	Describe the perspective of the study and relate this to the costs being evaluated.	Page 5, line 135-137
Comparators	7	Describe the interventions or strategies being compared and state why they were chosen.	Page 4, line 130-132
Time horizon	8	State the time horizon(s) over which costs and consequences are being evaluated and say why appropriate.	Page 5, line 152-154
Discount rate	9	Report the choice of discount rate(s) used for costs and outcomes and say why appropriate.	Page 8, line 241<br>Page 9, line 264
Choice of health outcomes	10	Describe what outcomes were used as the measure(s) of benefit in the evaluation and their relevance for the type of analysis performed.	Page 4, line 133-140
Measurement of effectiveness	11a	*Single study-based estimates:* Describe fully the design features of the single effectiveness study and why the single study was a sufficient source of clinical effectiveness data.	
	11b	*Synthesis-based estimates*: Describe fully the methods used for identification of included studies and synthesis of clinical effectiveness data.	Page 6, line 184-210
Measurement and valuation of preference based outcomes	12	If applicable, describe the population and methods used to elicit preferences for outcomes.	
Estimating resources and costs	13a	*Single study-based economic evaluation:* Describe approaches used to estimate resource use associated with the alternative interventions. Describe primary or secondary research methods for valuing each resource item in terms of its unit cost. Describe any adjustments made to approximate to opportunity costs.	
	13b	*Model-based economic evaluation:* Describe approaches and data sources used to estimate	Page 8, line 213-253

For peer review only

Section/item	Item No	Recommendation	Reported on page No/ line No
		resource use associated with model health states. Describe primary or secondary research methods for valuing each resource item in terms of its unit cost. Describe any adjustments made to approximate to opportunity costs.	
Currency, price date, and conversion	14	Report the dates of the estimated resource quantities and unit costs. Describe methods for adjusting estimated unit costs to the year of reported costs if necessary. Describe methods for converting costs into a common currency base and the exchange rate.	Page 8, line 246
Choice of model	15	Describe and give reasons for the specific type of decision-analytical model used. Providing a figure to show model structure is strongly recommended.	Page 5, line 143-176
Assumptions	16	Describe all structural or other assumptions underpinning the decision-analytical model.	Page 5, line 162-166<br>Page 8, line 230-240
Analytical methods	17	Describe all analytical methods supporting the evaluation. This could include methods for dealing with skewed, missing, or censored data; extrapolation methods; methods for pooling data; approaches to validate or make adjustments (such as half cycle corrections) to a model; and methods for handling population heterogeneity and uncertainty.	Page 7, line 190-201<br>Page 10, line 284-301
**Results**			
Study parameters	18	Report the values, ranges, references, and, if used, probability distributions for all parameters. Report reasons or sources for distributions used to represent uncertainty where appropriate. Providing a table to show the input values is strongly recommended.	Page 6, line 201-210<br>Page 8, line 241-253<br>Page 9, line 264-271<br>Page 10, line 276-278
Incremental costs and outcomes	19	For each intervention, report mean values for the main categories of estimated costs and outcomes of interest, as well as mean differences between the comparator groups. If applicable, report incremental cost-effectiveness ratios.	Page 11, line 320-323<br>Page 14, line 393-398
Characterising uncertainty	20a	*Single study-based economic evaluation:* Describe the effects of sampling uncertainty for the estimated incremental cost and incremental effectiveness parameters, together with the impact of methodological assumptions (such as discount rate, study perspective).	
	20b	*Model-based economic evaluation:* Describe the effects on the results of uncertainty for all input parameters, and uncertainty related to the structure of the model and assumptions.	Page 13, line 354-389
Characterising heterogeneity	21	If applicable, report differences in costs, outcomes, or cost-effectiveness that can be explained by variations between subgroups of patients with different baseline characteristics or other observed variability in effects that are not reducible by more information.	Page 12, line 311-333
**Discussion**			
Study findings, limitations, generalisability, and current knowledge	22	Summarise key study findings and describe how they support the conclusions reached. Discuss limitations and the generalisability of the findings and how the findings fit with current knowledge.	Page 11, line 323-351
**Other**			

For peer review only

Section/item	Item No	Recommendation	Reported on page No/ line No
Source of funding	23	Describe how the study was funded and the role of the funder in the identification, design, conduct, and reporting of the analysis. Describe other non-monetary sources of support.	Page 3, line 79
Conflicts of interest	24	Describe any potential for conflict of interest of study contributors in accordance with journal policy. In the absence of a journal policy, we recommend authors comply with International Committee of Medical Journal Editors recommendations.	Page 3, line 81-84

For consistency, the CHEERS statement checklist format is based on the format of the CONSORT statement checklist

For peer review only
